# Supplementary material for: Effect of a multicomponent quality improvement strategy on sustained achievement of diabetes care goals and macrovascular and microvascular complications in South Asia at 6.5 years follow-up: Post hoc analyses of the CARRS randomized clinical trial
Source: PLoS Med. 2024 Jun 3;21(6):e1004335. doi: 10.1371/journal.pmed.1004335 (PMC11198027; doi:10.1371/journal.pmed.1004335)
Supplement: S1 Protocol — (PDF) [file pmed.1004335.s002.pdf]

Developing and Testing Integrated, Multi-factorial Cardiovascular Disease Risk Reduction Strategies in South Asia (CARRS Translation Trial)

A multi-site, individually randomized, controlled translation trial of integrated and comprehensive care strategies to reduce CVD risk among 1,120 T2DM patients in South Asia

Steering Committee of Investigators:

Dorairaj Prabhakaran, MD, MSc, DM *
Public Health Foundation of India (PHFI)
New Delhi, India

Nikhil Tandon, MD, PhD
All India Institute of Medical Sciences (AIIMS)
New Delhi, India

Viswanathan Mohan, MD, FRCP, PhD, DSc, FNASc
Madras Diabetes Research Federation (MDRF)
Chennai, India

K. Srinath Reddy, MD, MSc, DM
Public Health Foundation of India (PHFI)
New Delhi, India

K.M. Venkat Narayan, MD, MSc, MBA, FRCP
Emory University
Atlanta, United States

Muhammad Masood Kadir, MBBS, FCPS, MPH
Aga Khan University,
Karachi, Pakistan

Mohammed K. Ali, MBChB, MSc, MScGH
Emory University
Atlanta, United States

* *Principal Investigator*

Supported by:
National Heart Lung and Blood Institute, National Institutes of Health, USA
Contract # HHSN268200900026C
&
Ovations Chronic Disease Initiative, UnitedHealth Group, USA

PROTOCOL Version 2.6: 21May2011

PROTOCOL

TABLE OF CONTENTS

Page

ABBREVIATIONS AND ACRONYMS 5

STEERING COMMITTEE OF INVESTIGATORS 7

PARTICIPATING CLINIC SITES........ **ERROR! BOOKMARK NOT DEFINED.**

SUMMARY 9

1. STUDY OBJECTIVES 11

1.1 Primary Objective 11

1.2 Ancillary Objective 12

2. BACKGROUND AND RATIONALE 12

2.1 Background 12

2.2 Study Rationale 12

2.2.1 Evidence Supporting Risk Factor Control in Reducing Adverse Cardiovascular Outcomes 13

2.2.2 Gaps in Implementation 14

2.2.3 Evidence for Quality Improvement Delivery Strategies 14

3. STUDY DESIGN 16

4. SELECTION AND ENROLLMENT OF PARTICIPANTS 18

4.1 Population and Sites 18

4.2 Inclusion Criteria 18

4.3 Exclusion Criteria 18

4.4 Study Enrollment Procedures 19

4.4.1 Methods of Recruitment 19

4.4.2 Informed Consent 20

4.4.3 Screening/Baseline Assessment 21

4.4.4 Randomization 21

5. STUDY INTERVENTIONS 22

5.1 Intervention Arm 22

5.1.1 Decision-Support Software 23

5.1.2 Care Coordinators 23

5.2 Control Arm 24

5.2.1 Research Officer 24

6. STUDY PROCEDURES 25

6.1 Schedule of Evaluations 26

PROTOCOL

6.2 Description of Evaluation Schedule 27
6.2.1 Screening (Pre-randomization) 28
6.2.2 Randomization and Baseline 29
6.2.3 Follow-up 12 monthly Visits 30
6.2.4 3 monthly and Intermediate Visits 31
6.2.5 Completion/Final Evaluation 32

7. SAFETY ASSESSMENTS 34

7.1 Specification of Safety Parameters 34

7.2 Adverse Events and Serious Adverse Events 34

7.3 Reporting Procedures 36

7.4 Safety Monitoring 36

8. INTERVENTION DISCONTINUATION 37

9. STATISTICAL CONSIDERATIONS 38

9.1 General Design Issues 38

9.2 Sample Size and Randomization 38

9.3 Interim Analyses and Stopping Rules 39

9.4 Outcomes 39
9.4.1 Primary outcome 40
9.4.2 Secondary outcomes 40

9.5 Data Analyses 43

9.5.1 Quantitative Analysis 43

9.5.2 Analysis for Cost-Effectiveness 43

9.5.3 Qualitative Analysis 46

10. DATA COLLECTION AND QUALITY ASSURANCE 47

10.1 Data Collection Forms 47

10.2 Data Management 47

10.3 Quality Assurance 47
10.3.1 Training 47
10.3.2 Quality Control Committee 48
10.3.3 Trial Documentation / Data Quality 48
10.3.4 Monitoring 48

11. PARTICIPANT RIGHTS AND CONFIDENTIALITY 50

11.1 Institutional Review Board (IRB) Review 50

11.2 Informed Consent Form 50

11.3 Participant Confidentiality 50

11.4 Study Discontinuation 50

12. STUDY ORGANIZATION 51

12.1 Overview 51

12.2 Clinical Sites 51

PROTOCOL

12.3 The Research Coordinating Center 51
12.4 NHLBI/Westat 51
12.5 Central Laboratory 52
12.6 The CARRS Trial Steering Committee, Committee of Investigators, Subcommittees, the Executive Committee, and the Advisory Committee 52
12.7 Data Safety Monitoring Board (DSMB) 53
12.8 Conflict of Interest Policy 53

13. PUBLICATION OF RESEARCH FINDINGS 54

14. FUNDING 54

15. ADMINISTRATIVE CLAUSES 54

16. REFERENCES 55

17. SUPPLEMENTS/APPENDICES 62

17.1 Appendix 1: Patient Information Sheet and Informed Consent Form 62
17.2 Appendix 2: Physician Interview Information Sheet and Consent Form 67
17.3 Appendix 3: Cardiovascular Disease (CVD) Risk Management Algorithm-Guidelines 69
17.4 Appendix 4: Subcommittee Responsibilities 72
17.5 Appendix 5: Case Report FORMS (CRFs) 73

1. Form A – Screening Part 1 v1.2-17JUL2010
2. Form B – Screening Part 2 v1.2-17JUL2010
3. Form C – Baseline_Randomization v1.3-29JUL2010
4. Form D – 3 monthly Visit_Intervention v1.2-17JUL2010
5. Form E – Follow-up 12 monthly_All v1.3-29JUL2010
6. Form F – Close-out_All v1.3-29JUL2010
7. Form G – Eye Exam v1.2-17JUL2010
8. Form I.c – Intermediate Visit_Control v1.2-17JUL2010
9. Form I.i – Intermediate Visit_Intervention v1.2-17JUL2010
10. Form K – Interview Guide_Physician v1.2-17JUL2010
11. Form Z – Intervention Management Plan v1.2-17JUL2010

FIGURES and TABLES

Figure 1: Allocation Scheme........ 16
Figure 2: Study Periods........ 17
Figure 3: Timeline of CARRS Translation Trial........ 17
Figure 4: Diagram of Care Received by Intervention and Control Arms........ 22
Table 1: Sample Size Calculations for CARRS Translation Trial 37
Table 2: Study Measures for CARRS Translation Trial 40
Table 3: Description of assessment questionnaires of secondary outcomes........ 42
Table 4: Quality Assurance Strategy for CARRS Translation Trial........ 49
Figure 5: Study Organization of CARRS Translation Trial........ 53
Figure 6: CARRS Trial Committee........ 54

PROTOCOL

ABBREVIATIONS AND ACRONYMS

ACE-i	Angiotensin Converting Enzyme (ACE) – Inhibitors
ACS	Acute Coronary Syndrome
AE	Adverse Event
ALT	Alanine Aminotransferase
ARB	Angiotensin II Receptor Blocker
BMI	Body Mass Index
BP	Blood Pressure
CABG	Coronary Artery Bypass Grafting
CARRS	Center for Cardio-metabolic Risk Reduction in South Asia
CC	Care Coordinator
CEA	Cost Effectiveness Analysis
CHD	Coronary Heart Disease
CRF	Case Report Form
CS	Clinic Site
CTA	Clinical Trial Agreement
CV	Cardiovascular
CVD	Cardiovascular Disease
DKA	Diabetic Keto-acidosis
DSMB	Data Safety Monitoring Board
DSS	Decision Support Software
DTSQ	Diabetes Treatment Satisfaction Questionnaire
EC	Ethics Committee
ECG	Electrocardiogram
EHR	Electronic Health Records
EQ5D	European Quality of Life 5 Dimensions
ER	Emergency Room
HbA1c	HemoglobinA1c
HDL	High-Density Lipoprotein
HIC	High-Income Countries
HUI-3	Health Utility Index Mark-3
ICER	Incremental Cost-Effectiveness Ratio
ICMR	Indian Council of Medical Research
ICUR	Incremental Cost-Utility Ratio
IEC	Institutional Ethics Committee
IRB	Institutional Review Board
IWRS	Interactive Web Response System
LAR	Legally Accepted Representative
LDL	Low-Density Lipoprotein
LMIC	Low and Middle Income Countries
MDRF	Madras Diabetes Research Foundation
MI	Myocardial Infarction
NHLBI	National Heart Lung and Blood Institute
OHRP	Office for Human Research Protection
PHFI	Public Health Foundation of India
PP&A	Publication, Presentation and Ancillary Studies
QALY	Quality-Adjusted Life Years
RCC	Research Coordinating Center
RCT	Randomized Control Trial
SAE	Serious Adverse Event
SBP	Systolic Blood Pressure
SC	Steering Committee
SDSCA	Summary of Diabetes Self-Care Activities
T2DM	Type 2 Diabetes Mellitus
WHO	World Health Organization

PROTOCOL

STUDY ORGANIZATON OVERVIEW

The *National Heart Lung and Blood Institute (NHLBI), USA* and the *UnitedHealth Chronic Disease Initiative*, *USA* have established 11 global health collaborating *Centers of Excellence* (COE) in June 2009 to enable research and training to prevent/control chronic cardiovascular and lung diseases in developing countries.[1] One of the COE is the *Centre for cArdiometabolic Risk Reduction in South Asia* (COE-CARRS), based at the Public Health Foundation of India (New Delhi) with the following partner institutions: All India Institute of Medical Sciences (New Delhi, India), Madras Diabetes Research Foundation (Chennai, India) and Aga Khan University (Karachi, Pakistan). The developed country partner is Emory University (Atlanta, USA).

This *CARRS Translation Trial* is one of the COE-CARRS studies sponsored by NHLBI and UnitedHealth Chronic Disease Initiative. The Research Coordinating Centre (RCC), based in New Delhi, India, will coordinate and manage the trial being conducted at the Clinical Sites (CS). See ***Section 12: STUDY ORGANIZATION*** for more details.

PROTOCOL

STEERING COMMITTEE OF INVESTIGATORS

**Principal Investigator:**	**Full mailing address:**
**(1) Name: Dorairaj Prabhakaran**<br>Qualifications: MD, DM, MSc (Health Research Methodology)<br>Designation: Professor of Chronic Disease Epidemiology, Public Health Foundation of India; Executive Director, Centre for Chronic Disease Control<br>Telephone: +91-11-26850117/118, Fax: +91-11-26850588<br>E-mail: dprabhakaran@ccl.cindia.org	Dr. D Prabhakaran<br>Centre for Chronic Disease Control<br>C-1/52, 2nd Floor,<br>Safdarjung Development Area,<br>New Delhi – 110 016, India
**Co- Investigator(s)** involved in the study:	**Full mailing address:**
**(2) NAME: Nikhil Tandon**<br>Qualifications: MD, PHD<br>Designation: Professor of Endocrinology and Metabolism, AIIMS<br>Telephone: +91-11-26593433, Fax: +91-11-26588663<br>E-mail: nikhil_tandon@hotmail.com	Dr. Nikhil Tandon<br>Department of Endocrinology & Metabolism,<br>All India Institute of Medical Sciences<br>Biotechnology Block, 3rd Floor, Rm #311,<br>Ansari Nagar, New Delhi - 110 029, India
**(3) NAME: V. Mohan**<br>Qualifications: MD, FRCP (UK), FRCP (Glasg), PhD, DSc, FNASc<br>Designation: Chairman & Chief of Diabetology, Dr.Mohan's Diabetes Specialties Centre; Director & President, Madras Diabetes Research Foundation, Chennai<br>Telephone: +91-44-43968888, Fax: +91-44-43968888<br>E-mail: drmohans@vsnl.net	Dr. V. Mohan<br>Madras Diabetes Research Foundation<br>6B, Conran Smith Road, Gopalapuram,<br>Chennai – 600 086, Tamil Nadu, India
**(4) NAME: K. S. Reddy**<br>Qualifications: MD, MSc, DM<br>Designation: President, Public Health Foundation of India<br>Telephone: +91-11-4604-6000/6034; Fax: +91-11-46056714<br>E-mail:ksrinath.reddy@phfi.org	Dr. K. S. Reddy<br>Public Health Foundation of India<br>PHD House, 2nd floor, 4/2, Sirifort Institutional Area, August Kranti Marg,<br>New Delhi - 110 016, India
**(5) NAME: Muhammad Masood Kadir**<br>Qualifications: MBBS, FCPS, MPH (USA)<br>Designation: Professor, Community Health Sciences Department, Aga Khan University<br>Telephone: +92-21-34930051, Ext 4823; Fax: +92-21-34934294<br>Email: masood.kadir@aku.edu	Dr. Muhammad Masood Kadir<br>Department of Community Health Sciences<br>Aga Khan University<br>Stadium Road<br>Karachi 74800, Pakistan
**(6) NAME: K.M. Venkat Narayan**<br>Qualifications: MD, MSc, MBA, FRCP<br>Designation: Ruth and O.C. Hubert Professor of Global Health and Epidemiology, Professor of Epidemiology, Professor of Medicine, Emory University<br>Telephone: +1-404-712-1021, Fax: +1-404-727-4590<br>E-mail: knaraya@emory.edu	Dr. K.M. Venkat Narayan<br>Emory University,<br>Rollins School of Public Health<br>1518 Clifton Road, Rm #730,<br>Atlanta, Georgia, 30322, USA
**(7) NAME: Mohammed K. Ali**<br>Qualifications: MBChB, MSc, MScGH<br>Designation: Assistant Professor, Hubert Department of Global Health, Emory University<br>Telephone: +1-404-727-9776, Fax: +1-404-727-3350<br>E-mail: mkali@emory.edu	Dr. Mohammed K. Ali<br>Emory University,<br>Rollins School of Public Health<br>1518 Clifton Road, Rm #734,<br>Atlanta, Georgia, 30322, USA

PROTOCOL

PARTICIPATING CLINIC SITES

**SITE Principal Investigator(s)** involved in the study:	**Full mailing address:**
**(1) NAME: Mala Dharmalingam**<br>Qualifications: MD, DM<br>Designation: Director, Bangalore Endocrinology & Diabetes Research Centre<br>Mobile: +91-98452-08163; Tel: +91-80-6596-5758; Fax: +91-80-4128-1998 E-mail: bedrc.endo@gmail.com	Dr. Mala Dharmalingam<br>Bangalore Endocrinology & Diabetes Research Centre<br>#35, 5th Cross, Malleswaram Circle,<br>Bangalore - 560 003, Karantaka, India
**(2) NAME: Ganapathi Bantwal**<br>Qualifications: MD, DM<br>Designation: Professor and Head of Department of Endocrinology, St. John's Medical College & Hospital<br>Mobile: +91-94480-67318; Tel: +91-2206-5649/2550-3261; Fax: 91-80-2563-5313; E-mail: mallyaganapathi@rediffmail.com	Dr. Ganapathi Bantwal<br>Department of Endocrinology,<br>St. John's Medical College & Hospital<br>Sarjapur Road, Koramangala,<br>Bangalore - 560 034, Karantaka, India
**(3) NAME: Rakesh Sahay**<br>Qualifications: MD, DNB, DM<br>Designation: Professor, Department of Endocrinology, Osmania General Hospital<br>Mobile: +91-98495-97507; Tel: +91-40-2465-1667; Fax: +91-40-2465-1667; E-mail: sahayrk@gmail.com	Dr. Rakesh Sahay<br>Department of Endocrinology,<br>Osmania General Hospital<br>2nd Floor, Golden Jubilee Block, Afzalgunj,<br>Hyderabad - 500 012, Andra Pradesh, India
**(4) NAME: Bipin Kumar Sethi**<br>Qualifications: MD, DM<br>Designation: Consultant Endocrinologist, Department of Endocrinology, CARE Hospital<br>Mobile: +91=98480-21482; Tel: +91-40-3041-8888; Fax: +91-40-6625-8792; E-mail: bipinkumarsethi@yahoo.co.uk	Dr. Bipin Kumar Sethi<br>Department of Endocrinology,<br>CARE Hospital<br>Road No 1, Banjara Hills,<br>Hyderabad - 500 034, Andra Pradesh, India
**(5) NAME: Unnikrishnan AG**<br>Qualifications: MD, DM, DNB, MNAMS<br>Designation: Professor, Department of Endocrinology & Diabetes,<br>Mobile: +91-98460-05343; Office: 91-484-220-5343; Fax: 91-484-280-2020; E-mail: unnikrishnanag@aims.amrita.edu	Dr. AG Unnikrishnan<br>Department of Endocrinology & Diabetes,<br>Amrita Institute of Medical Sciences,<br>AIMS Ponekkara P.O.,<br>Kochi - 682 041, Kerala, India
**(6) NAME: Premlata Varthakavi**<br>Qualifications: MD, Dip NB (Endo)<br>Designation: Professor and Head of Department of Endocrinology<br>Mobile: +91-92244-80560; Tel: 91-22-2308-1490; Fax: 91-22-2307-5243; E-mail: premavar@hotmail.com	Dr. Premlata Varthakavi<br>Department of Endocrinology,<br>TNM College & BYL Nair Ch. Hospital<br>Dr. A. L. Nair Road, Mumbai Central,<br>Mumbai - 400 008, Maharashtra, India
**(7) NAME: Vijay Viswanathan**<br>Qualifications: MD, PhD, FRCP, MNAMS<br>Designation: Managing Director, Diabetes Research Centre & MV Hospital for Diabetes<br>Mobile: +91-98400-55535; Tel: +91-44-2595-4913; Fax: +91-44-2595-4919;E-mail: dr_vijay@vsnl.com	Dr. Vijay Viswanathan<br>MV Hospital for Diabetes & Diabetes Research Centre<br>No 4, West Madha Church Street,<br>Royapuram,<br>Chennai - 600 013, Tamil Nadu, India
**(8) NAME: Abdul Jabbar**<br>Qualifications: MBBS, FRCP<br>Designation: *Professor* and Head, Section of Endocrinology and Diabetes<br>Mobile: +92-33330-46798; Tel: +92-21-493-0051; Fax: 92-21-3493-4294<br>E-mail: abdul.jabbar@aku.edu	Dr. Abdul Jabbar<br>Aga Khan University of Medical Sciences<br>Department of Medicine, Section of Endocrinology and Diabetes<br>Karachi 74800, Pakistan

PROTOCOL

SUMMARY

Study Title: Developing and Testing Integrated, Multi-factorial Cardiovascular Disease Risk Reduction Strategies in South Asia (CARRS Translation Trial)

Objectives: To test whether a clinic-based case management intervention to reduce cardiovascular disease (CVD) risk among Type 2 diabetes patients in South Asia, is more effective and sustainable compared to existing care. The intervention uses non-physician care coordinators to help patients improve their care and follow-up as well as a decision-support software to help the physicians care for their patients.

Rationale/Background: Cardiovascular diseases are currently the leading cause of death globally and Asian Indians will account for between 40-60% of the global CVD burden within the next 10-15 years. Risk factor control and preventive care are effective in reducing CVD events and mortality. The greatest gains in CVD prevention have been seen when early and target-driven interventions address multiple risk factors *together*. However, achieving control of even individual risk factors (blood glucose, blood pressure, or blood lipid targets) is poor, globally. Quality improvement schemes, like the proposed intervention, have shown promise in high-income countries, but are untested in South Asia; a region with a population at extraordinarily high CVD risk.

Participants & Design/Intervention: The study will involve a total of 1120 patients attending 8 established out-patient clinics in South Asia (140 patients at each clinic) over an average of 30 months. Since the study aims to reduce CVD risk factors like elevated blood glucose, blood pressure, and cholesterol; participants eligible for the study will be patients with Type 2 diabetes and poorly controlled blood glucose (Hemoglobin A1c [HbA1c] ≥ 8.0%) as well as either high blood pressure (systolic blood pressure [SBP] ≥ 140 mmHg) and/or high cholesterol (LDL-cholesterol ≥ 130 mg/dl).

Patients enrolled in the trial will be randomly assigned to either the control (existing care) or the intervention group. Half of the patients will be randomized to the intervention, which includes a decision-support software and non-physician care coordinator. The decision-support software follows evidence-based guidelines, stores patient health records, and provides patient management prompts (e.g. remind patients about self-care and appointments. A non-physician care coordinator facilitates follow-up and helps patients with their self-management of their CVD risks. The control group will receive the existing care at the clinical site. All study physicians will have equal access to the evidence-based management guidelines.

Outcomes: The study will compare the proportion of participants from the control and the intervention group that achieve the primary outcome, control of multiple risk factors: blood glucose AND either blood pressure or cholesterol, or both. Other outcomes that will be compared between the two groups include: control of the individual CVD risk factors, quality of care, patient health-related quality of life and treatment satisfaction, and sustainability assessments of cost-effectiveness and acceptability by patients and providers.

Timeline & Evaluations: The total trial duration is about 3.5 years, from mid-August 2010 to December 31, 2013. The participant enrollment period will range from 1 to 1.5 years, depending on the starting time for each clinical site.

Potential participants will be identified for enrollment per inclusion/exclusion criteria from clinical records or referrals from clinic physicians. Potential participants will be invited for two pre-randomization visits for informed consent (which covers screening and study) and screening. The screening includes a brief history; anthropometric, blood pressure and heart rate measurements; urine sample, and fasting venous blood sample for basic biochemical tests (glucose, HbA1c, lipid profile,

PROTOCOL

creatinine, potassium, sodium, and ALT). At the baseline/randomization visit, all consenting, eligible participants will undergo further assessments including detailed history and physical examination, questionnaires (self-management, quality of life, treatment satisfaction, cost of care), ECG, foot examination, and eye examination (visual acuity and dilated pupil fundoscopy). The participants' baseline evaluation will be documented in the software.

Each participant will be followed for an average of 30 months, with yearly study-related visits for both the intervention and control group to collect study data which will include an update in history, physical exam, lab investigations, and questionnaires on quality of life/treatment satisfaction/costs of care and acceptability of the intervention. Site physicians will also be interviewed (with informed consent) regarding acceptability of the intervention at baseline and annually. All the annual trial-related biochemical and complication screening investigations (ECG, eye, foot, and urine examinations) will be paid for by the study.

Outside the annual visits, the intervention group is encouraged to attend the clinic at least every 3 months (to follow-up on blood glucose, blood pressure and lipid control and self-care), which are facilitated by the non-physician care coordinator. The control group will continue with visits as per existing care. The care coordinator will collect data for the intervention group and a distinct individual (a local research assistant) at the site will collect the data for the control group, to avoid contamination between groups.

All study staff involved in the trial will be trained in human subjects protection as well as the procedures of the trial including proper informed consent, enrollment, and follow-up of participants.

Timeline: In late August 2010, Phase 1 of the trial will begin at 3 clinic sites, to assess intervention feasibility (recruitment and retention, continuity of care), establish procedures, and engage in team-building. The 3 clinical sites for Phase 1 (Vanguard Phase) will be selected based on the following criteria: 1) readiness of site to manage the trial, 2) history of clinical research experience, and 3) capacity to recruit participants according to the inclusion/exclusion criteria. Upon successful completion of this first phase within 6 months, the other 5 sites will join the study as Phase 2 of the trial.

Data Analysis: All data analysis will be conducted at the Research Coordinating Centre in New Delhi, by designated staff. Quantitative data analysis will be performed using SAS or STATA statistical analysis software packages, using the intention-to-treat principle. The following will be assessed: descriptive statistics, differences of baseline and close-out evaluations between and within the intervention and the control groups.

Cost-effectiveness will be calculated by assessing the incremental cost-effectiveness ratio (ICER): the numerator represents intervention versus control group *net costs* (only patient care costs, not investigator time and planning efforts, plus out-of-research costs to the clinic and patients), and the denominator represents the net "effectiveness" of outcomes, including the primary outcome (multiple risk factor control) and improved life measures, calculated from the quality of life questionnaires.

Qualitative data analysis will be conducted using MAXqda software. Key themes will be identified from the transcribed interviews, and the themes will be compared to identify specific issues relevant to the intervention and control groups as well as specific sub-groups.

PROTOCOL

1. STUDY OBJECTIVES

AIM: To test the effectiveness and sustainability of an intensive and comprehensive health care intervention to reduce cardiovascular disease (CVD) risk in South Asia, using a randomized controlled trial, in 1,120 patients with Type 2 diabetes (T2DM) who are at moderate-to-high risk for CVD (with HbA1c ≥ 8.0% and at least one of comorbid hypertension [SBP ≥ 140 mmHg] or dyslipidemia [LDL-cholesterol ≥ 130 mg/dl]).

The intervention includes decision-support software and non-physician care coordinators to target multi-factorial CVD risk control of blood glucose, blood pressure, and lipids, and achieve recommended processes of care. The participants will be randomized to the intervention or to standard care.

1.1 Primary Objective

To test the effectiveness of a CVD risk reduction intervention using clinical decision-support software and non-physician care coordinators, compared to the control group of standard care, in Type 2 diabetes patients attending established out-patient clinics in South Asia. The primary outcome to be examined is the between-group difference in proportions achieving multiple risk factor control targets (glycemic control and either control of blood pressure or blood lipids, or both).

Hypothesis: Compared to the control group, the intervention group will, on average, demonstrate a *relative* 40% greater proportion of participants (absolute 28% vs. 20%) achieving multiple CVD risk factor control targets (at least two targets including HbA1c < 7.0% AND at least one of BP < 130/80 mmHg OR LDL-cholesterol < 100 mg/dl[1]).

The effectiveness of the intervention will also be assessed by comparing the intervention and the control group regarding the following secondary outcomes:

1) Single risk factor control targets, as demonstrated by:
   a. at least an absolute 10% point greater proportion of participants in the intervention group achieving glycemic control (HbA1c < 7.0%);
   b. at least an absolute 10% point greater proportion of participants in the intervention group achieving blood pressure control (systolic BP < 130 and diastolic BP < 80 mmHg); and
   c. at least an absolute 10% point greater proportion of participants in the intervention group achieving lipid control (LDL-cholesterol < 100 mg/dl [<70 mg/dl for individuals with history of CVD event]);

2) Quality of care measures, as demonstrated by patient and provider adherence to:
   a. currently advocated CVD risk management guidelines (i.e. proportion of patients prescribed and/or using lipid- or BP-lowering medication, where indicated; proportion of patients smoking who have stopped; proportion of patients who were given lifestyle modification advice and/or adhering to dietary and physical activity targets) and
   b. evidence-based processes of care (i.e. prescription and/or use of low-dose aspirin and/or RAS-modifiers; and annual eye, foot, and urine examinations); and

PROTOCOL

[1] NOTE: The target LDL-cholesterol < 100 mg/dl is for those with NO history of CVD event, but for an individual with a history of CVD event, a target LDL-cholesterol < 70 mg/dl is advised. (ADA 2009, (85))

3) Patient-related outcomes of:
   a. health-related quality of life; and
   b. treatment satisfaction.

1.2 Ancillary Objective

1) To evaluate the sustainability of the intervention using the following methods:
   a. Cost-effectiveness of the intervention by assessing the incremental costs and effectiveness of the intervention versus standard care for the clinic facility.
   b. Assessments of acceptability of the intervention from the provider perspective through interviews and the patient perspective in the final questionnaire; and follow-up of participants who dropped out of the program to explore reasons for discontinuing involvement

2. BACKGROUND AND RATIONALE

2.1 Background

It is estimated that coronary heart disease (CHD), cerebrovascular disease, and diabetes together account for 30% of global mortality and 80% of these deaths occur in low- and middle-income countries (LMIC).[2-4] Diabetes commonly co-exists with obesity, hypertension,[5] and lipid abnormalities (elevated triglycerides, low HDL-cholesterol, and abnormal LDL-cholesterol sub-fractions) and is a central feature accelerating athero-thrombotic CVD, while also the leading cause of adult-onset blindness, non-traumatic amputations and kidney failure worldwide. The addition of these inter-related risk factors and co-morbidities results in a multiplicative, rather than additive, amplification of risk.[6]

In people of South Asian origin, diabetes, cardio-metabolic risk factors[7, 8] and events[9, 10] occur at younger ages and lower body mass indices (BMI) when compared to other ethnic groups,[10-23] and are rapidly increasing with socioeconomic and nutrition transitions.[4, 24-26] The South Asia region includes three of the top ten countries in the world in terms of total diabetes subjects (India, Pakistan, and Bangladesh)[27] and is the region with the highest number of diabetes-related deaths currently.[28] Asian Indians, as a group, are projected to account for between 40-60% of the global CVD burden within the next 10-15 years.[29] Furthermore, 35% of CVD-related deaths in India occur in those between 35-64 years of age as compared to only 12% in the U.S.[30]

2.2 Study Rationale

Robust evidence has demonstrated the efficacy of risk factor control and preventive processes of care in reducing CVD events and mortality. Since CVD risk factors do not occur in isolation, the greatest gains in CVD prevention have been seen when earlier, intensive, target-driven, multi-factorial interventions have been applied *together*. However, implementation of evidence-based recommendations and achieving control of even individual risk factors (Hemoglobin A1c, blood pressure, or blood lipid targets) is woefully sub-optimal, globally. In other words, the translation of proven interventions from controlled environments to delivery in real-life settings remains a major challenge. This translation trial tests an intensive, multi-factorial CVD risk reduction intervention in South Asia using integrated, low-cost management strategies of clinical decision-support for providers and individualized coordinated care for patients.

PROTOCOL

2.2.1 **Evidence Supporting Risk Factor Control in Reducing Adverse Cardiovascular Outcomes**

Unequivocal evidence has demonstrated that individual risk factor control, modification of lifestyle choices [31, 32 33-36] and application of proven agents [e.g., aspirin [37, 38] and renin-angiotensin system (RAS) modifiers [39-42]] are associated with absolute and relative risk reductions (RRR) of disabling, and often fatal, complications. Statin use for lipid control reduces LDL-cholesterol by 27-40% with subsequent decreases in CVD events and mortality by approximately a third [43, 44] in persons with and without diabetes or previous acute coronary syndromes (ACS).[45] This benefit extends to subgroups of elderly or those with already controlled LDL-cholesterol fractions (<2.6 mmol/l or 100 mg/dl).[45, 46]

Several large randomised trials, sub-studies [38, 47-51] and meta-analyses [52, 53] have shown benefits of blood pressure (BP) lowering in reducing chronic kidney disease (CKD), [42, 54] and remarkable reductions in CVD events (25%)[46, 55, 56] and mortality (51%).[38] The supplementary cardio- and reno-protective effects (anti-atherosclerotic, reducing arterial stiffness, and improving endothelial function)[39, 50, 57-60] of RAS-modifiers are motivating more extensive application of these agents, especially in people with diabetes.

Long-term antiplatelet therapy for *primary prevention* of CVD events and mortality in *moderate-risk patients* is under further study due to the risk of extracranial and gastrointestinal bleeding.(60) However, strong evidence still exists for the efficacy of low-dose aspirin and/or clipidogrel therapy to reduce CVD events and mortality in *high-risk patients*, which includes individuals with diabetes and for *secondary prevention*.[37, 61-64]

Intensively targeting smoking cessation in high-risk patients is beneficial. Such intensively- managed patients have a 3.5 times greater likelihood of maintaining abstinence from smoking (33% vs. 9%; p<0.0001) compared to standard care, and have a RRR of 44% (95% CI, 16-63%; p=0.007) and 77% (95% CI, 27-93%; p=0.014) in hospitalization and all-cause mortality, respectively.[65, 66]

Although the large multi-center studies [67-72] have not shown a direct benefit of glycemic control on CVD endpoints, they have reinforced the importance of earlier,[67, 73, 74] as well as more comprehensive, multi-factorial risk management [72, 75-78] in reducing the negative effects of prolonged metabolic disturbance in high-risk subjects. Recently-published long-term follow-up studies of large randomized controlled trials (RCT)[74, 79] have demonstrated a delayed effect of previous glycemic control on macrovascular outcomes, a "metabolic memory" of sorts. Furthermore, glycemic control has proven benefits for reducing the incidence of microvascular complications.[47, 80]

Corollaries of this body of evidence, therefore, are that risk reduction is more marked, and hence of particular benefit, in subjects at high-risk for CVD, such as those with diabetes.[5] Secondly, intensive and multi-faceted risk factor management (i.e., intensively applying interventions with independent benefit *together*)[5, 81, 82] provides remarkable decreases in CVD morbidity and mortality, in those with and without existing CVD.[46, 83-85] The Steno-II study investigated such an approach in type 2 diabetes mellitus (T2DM) patients with microalbuminuria and demonstrated declines in metabolic parameters (HbA_1c, BP, lipids) and improvements in use of aspirin and angiotensin-converting enzyme inhibitors (ACE-i), which translated into sizeable gains in prevention of CVD (53% reduction) over 7.8 years [83] and lower CVD mortality (59% reduction) over 13.3 years of follow-up.[84] However, the larger trials also caution that therapies should be individualized, whereby proven efficacious doses and regimens are considered and balanced against contraindications and serious adverse effects.

PROTOCOL

2.2.2 Gaps in Implementation

Advocacy for managing all risks and achieving optimal targets in CVD reduction, and robust evidence from large trials, have been assimilated into commonly available clinical practice guidelines.[86] However, implementation of evidence-based interventions is far from optimal, globally.[87-91]

The few reports from India [DEDICOM[92] and DiabCare Asia[93] surveys)] suggest a similar pattern of poor quality of chronic diseases care [92-94] where glycemic, lipid, and BP targets are not achieved in almost half the subjects surveyed, and only 17.5% of patients are using aspirin. The World Health Organization (WHO) Prevention of Recurrences of Myocardial Infarction and Stroke (PREMISE)[94] study investigated processes of care in clinic-based ACS patients in ten LMIC. In India, Pakistan, and Sri Lanka, although the use of aspirin ranged from 62.7-96.1%, beta-blocker use (8.7-60.5%), ACE-i use (5.3-45.1%) and statin use (2.3-38.4%) were far from encouraging in these patients, with the lowest percentages reported in Sri Lanka. Statin use, for example was primarily influenced by presence of hypercholesterolaemia or previous revascularization, but not co-existing high blood sugar and high BP. These results are concerning as over half of these patients with prior CHD and strokes were under age 60, while 47% had two or more CVD risk factors. The CREATE Registry [95] in India reported similar patterns of prescription and uptake of evidence-based preventative medications like ACE-i, statins and beta-blockers in ACS patients, especially among the poorer patients (who therefore suffered higher mortality rates). Poor clinical practices, poor adherence, and likely poor clinic accessibility help explain the remarkable proportion (54%)[96] of diabetes patients that develop severe late-stage complications.

The quality of chronic care delivery is rooted in the complex interactions among providers, patients, intricacies of the disease itself, and the system of care.[97, 98] In South Asia, characteristic disparities, the mix of public-private health care, low levels of awareness, and the asymptomatic, chronic nature of non-communicable risk factors and diseases perpetuate delays in diagnosis, inertia to seek care, and effective self-management of risks. At the provider- and systems-level, lack of decision support strategies limit risk stratification, implementation of evidence-based guidelines, intensification and follow-up of treatment regimens, and coordination of various aspects of care in a structured manner. These are all factors that will be considered in designing an appropriate multi-faceted intervention to overcome barriers at different levels.

2.2.3 Evidence for Quality Improvement Delivery Strategies

Several organizational strategies, singly or in combination, have been shown to improve processes of care,[99-101] intermediate risk factors in CMD management,[102-105] and/or patient satisfaction.[104, 106-109] Interventions have included: use of non-physician health workers as "care coordinators";[102-105, 110-117] implementation of electronic registries for identification, risk stratification, tracking and reminder notification;[118, 119] web-based self-management assistance;[120, 121] aggressive implementation of evidence-based guidelines; audit and feedback to physicians;[122, 123] provider incentives;[124] structured care;[125] use of care teams;[107] group visits; and reducing financial or other barriers to accessing care.[111] Quality improvement principles have also been applied to tertiary prevention of ACS in India, where benefits were shown.[126, 127] The best outcomes, however, have been shown when multiple approaches, applied *together*,[125, 128, 129] and *customized* for individuals,[130-132] have been utilized for follow-up and coordination of patient care by interdisciplinary teams. Multi-faceted care has therefore been advocated for out-patient, clinic, and community-based cardio-metabolic care.[133]

In particular, the use of care coordinators is a feasible option to build local capacity through low cost training and adaptation of skills in resource-poor settings.[134] Non-physician care coordinators, working as intermediary liaisons between patients and providers, are able to triage, put algorithm-derived management

PROTOCOL

plans into practice, encourage effective self-monitoring and adherence to therapy, and coordinate and monitor patient follow-up and investigations, thereby facilitating care in fragmented health systems. Trials evaluating this model have demonstrated at least modest, achievable improvements in processes of care[99, 100, 105, 106, 108, 110, 114, 116] and sometimes corresponding improvements in intermediate biochemical outcomes.[104, 107, 112-114, 116] In addition, patients have reported greater satisfaction. Cost-effectiveness analyses suggest that additional costs of disease case management are counter-balanced by the *potential* to reduce emergency-room visits, hospitalization, and disease complications.[112, 113, 135, 136]

When considering implementation of innovative organizational changes for *less developed countries*, the literature supporting multi-factorial, multi-faceted, integrated cardio-metabolic risk management interventions is limited in that most studies:

- have been confined to high-income countries (HIC) with very few studies conducted in resource-constrained areas such as South Asia, a region host to a population at unusually high risk;
- have examined small, regionally endogenous sample populations (< 300 subjects) at single sites, and have only followed patients for an average of 1-2 years;
- have investigated specific processes of care and/or single risk factor control as surrogate endpoints, and almost none have tested comprehensive multi-factorial risk management; and
- have not built in plans during design and implementation to evaluate cost-effectiveness, and to consider issues pertinent to scaling up and sustainability, such as low-cost interventions.

Even the single developed country study[135] in the U.S. (North Carolina) which reports 53% reduction in CVD event rate over 6 years of follow up in 1,185 patients, while demonstrating 50% less emergency room (ER) visits and 46.5% lower CVD-related costs, can only attest to pre- and post-intervention analysis with no control group. The opportunity therefore exists to test the sustained effectiveness of targeted, multi-faceted cardio-metabolic risk reduction strategies, as mentioned above, with a more robust randomized controlled trial. Also, patient case management interventions that can be added to existing systems of care without requiring a complete overhaul may be beneficial for low-resource settings.

Given the elevated risk in the South Asian population, combined with the unregulated and disjointed mix of health care providers, we anticipate that the intervention strategies proposed (use of non-physician care coordinators and computerized decision-support system) will yield realizable benefits and may be achieved at low cost.

PROTOCOL

3. STUDY DESIGN

The study is a multi-site, individually randomized, controlled parallel group translation trial testing a comprehensive, multi-factorial CVD risk reduction intervention (of clinical decision-support software and care coordinators) in 1,120 Type 2 diabetes (T2DM) patients attending 8 established out-patient clinics in South Asia, for a mean follow-up of 30 months. The intervention group will be compared to a control group receiving standard care. Outcomes will report between-group differences:

- The primary outcome is a sustained relative difference in those achieving multiple risk factor targets (glycemic and BP or lipid control, or both) in the intervention group, compared to the control group.
- Secondary outcomes include between-group percentage point differences in achieving individual risk factor targets for glycemia, lipid, and BP management; quality of care measures (adherence to CVD risk factor management guidelines and evidence-based preventative and therapeutic processes of care); patient health-related quality of life and treatment satisfaction; and sustainability assessments of cost-effectiveness and acceptability by patients and providers.

A total of 1,120 patients with T2DM at moderate-to-high risk for CVD (with Hemoglobin A1c [HbA1c] ≥ 8.0% and at least one of comorbid hypertension [SBP ≥ 140 mmHg] or dyslipidemia [LDL-cholesterol ≥ 130 mg/dl]) will be enrolled in the trial; 140 at each of the 8 out-patient, urban clinics in South Asia. The 140 eligible, consenting participants at each site will be individually randomized to standard care (n=70) OR to the intervention (n=70) which targets glucose, blood pressure and lipid control, and processes of care (aspirin use; ACE-inhibitor use; smoking cessation advice; lifestyle modification advice; and regular eye, foot, ECG and urine exams). The allocation scheme is shown in **Figure 1**.

Figure 1: Allocation Scheme

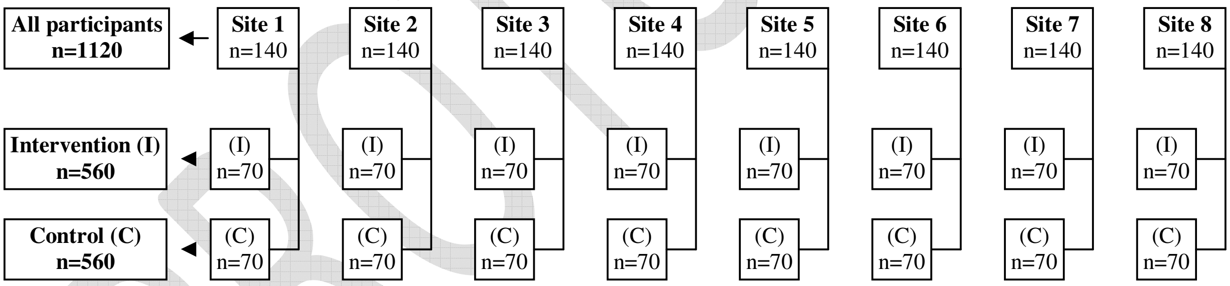

The intervention strategies follow current internationally accepted evidence-based CVD risk management algorithm-guidelines for individuals with diabetes, and have been adapted with the help of experienced physicians to increase relevance to the South Asian population.

The intervention utilizes two major health care management strategies: (1) a web-enhanced decision-support software to: store patient health records; provide automated decision-support prompts for patient management; and remind providers and participants of the guideline-recommended care processes; and (2) a non-physician care coordinator to: facilitate coordinated care according to the CVD risk factor management guidelines; provide individualized follow-up according to baseline risk and compliance; empower and encourage patients to achieve management targets (i.e. smoking cessation, lifestyle modification, treatment adherence); and coordinate guidance from a multi-disciplinary team (composed of a physician, dietician, social worker, educator, etc.). The control group will receive the existing standard care existing at the clinical site. All study physicians will have equal access to the evidence-based management guidelines.

Potential participants will be identified for enrollment per inclusion/exclusion criteria from clinical records or encounters and contacted to participate in the study. Potential participants will be invited for two pre-randomization visits for informed consent and screening (brief history; anthropometric, blood pressure and

PROTOCOL

heart rate measurements; and fasting venous blood sample for glucose, HbA1c, lipid profile, creatinine, potassium, sodium, and ALT). At the baseline/randomization visit, all consenting, eligible participants will undergo further assessments including detailed history and physical examination, questionnaires (self-management, quality of life, treatment satisfaction, cost of care), and preventive exams including urine analysis (albumin:creatinine ratio), ECG, and foot and eye examinations.

Patient involvement in the trial will span approximately 3.5 years. The participant enrollment period will range from 1 to 1.5 years from the start of the overall study, depending on the trial starting time for each clinical site. Each participant will be followed for an average of 30 months, with follow-up study visits every 12 months for all patients, and regular 3-monthly visits for the intervention group, with a provision to collect all other intermediate visits by all participants. The study periods are shown in **Figure 2**.

The trial will be conducted in two phases. In mid-August 2010, Phase 1 of the trial will begin at 3 clinic sites to assess intervention feasibility (recruitment and retention, continuity of care), establish procedures, and engage in team-building. The 3 clinical sites for Phase 1 (Vanguard Phase) will be selected based on the following criteria: 1) readiness of site to manage the trial, 2) history of clinical research experience, and 3) capacity to recruit participants according to the inclusion/exclusion criteria. Upon successful completion of this first phase within 5-6 months, the other 5 sites will join the study as Phase 2 of the trial. The timeline is shown in **Figure 3**.

Figure 2: Study Periods

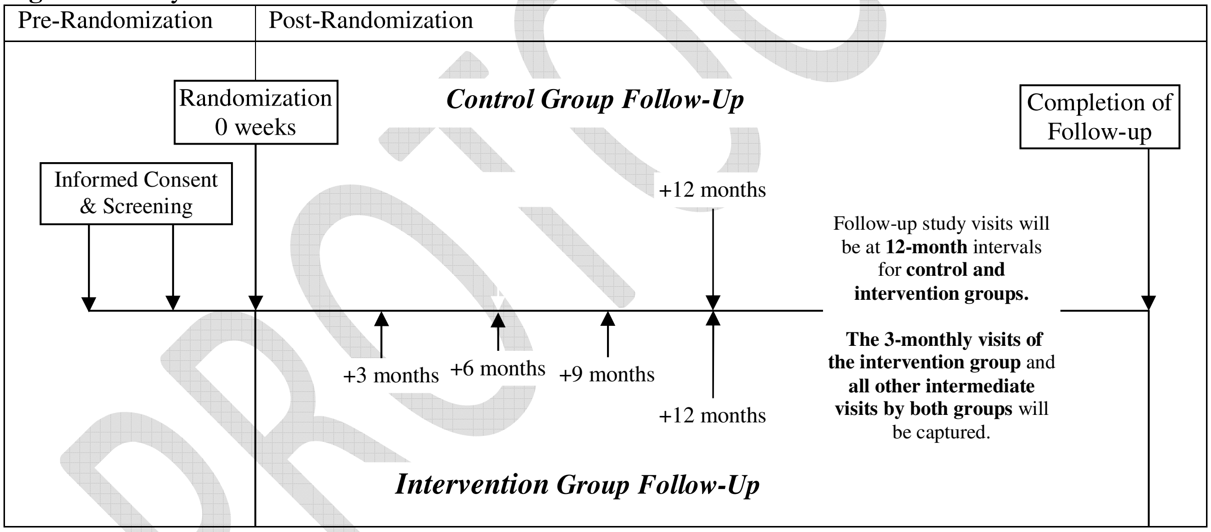

Figure 3: Timeline of CARRS Translation Trial (DSS = decision-support software; CC= care coordinator)

	Jan-July 2010	**Enrolment:** **Phase 1: ~1.5 years** (Aug 2010-Dec 2011) **Phase 2: 1 year** (Jan-Dec 2011)		**Follow-up:** 2 year minimum (Jan 2012-Dec 2013)		Jan 2014-June 2014
**Activities**	**-7 mo**	**Year 1** Aug2010-June2011	**Year 2** July 2011-June 2012	**Year 3** July 2012-June 2013	**Year 4** July 2013-June 2014	**Extension**
**PILOT- DSS**						
**PILOT- DSS&CC**						
**PHASE 1 (Vanguard)** (3 sites, n=420)						
**PHASE 2:** additional 5 sites, n=700 → *full 8 sites, n=1120*						
**Monitoring, Data Analysis & Reporting**						
Obtain support for trial Extension (follow-up for events/mortality)						

4. SELECTION AND ENROLLMENT OF PARTICIPANTS

4.1 Population and Sites

Given the heightened risk of athero-thrombotic CVD events [137-140] and mortality [6, 141-144] among people with T2DM, and the high background prevalence of T2DM [8, 12, 17, 25, 145] and deficiencies in implementation of known interventions [92-95] in South Asia, *T2DM patients in South Asia represent an ideal high-risk study population* for an intervention to reduce CVD risk.

Early recognition, intensive therapy[67, 146], and patient empowerment for effective self-management, combined with chronic, regular preventative attention are key facets of cardio-metabolic risk management.[83, 147-149] In particular, the treatment target recommendations of the American Diabetes Association[86] (which is endorsed in South Asia) for risk factors in diabetes patients include: HbA1c ≤ 7.0 %; LDL < 100 mg/dl (LDL<70 mg/dl for those with history of CVD event); HDL > 40 mg/dl (males) and HDL > 50 mg/dl (females); TG < 150 mg/dl; and BP < 130/80 mmHg. The *complexity of care required for T2DM patients make them prototypical candidates to investigate* methods of comprehensive management.[150-153] T2DM patients require sustained, comprehensive and intensive therapy to prevent costly morbidity and reduced life expectancy.[143]

Eight diabetes clinics in South Asia have been selected (see *Participating Clinic Sites*, pg. 7) through the networks available to our principal investigators. The lead physicians of these clinics are considered "Site Principal Investigators" and were participants in the CARRS Trial first investigators' meeting to engage their involvement/ownership in the trial, discuss trial procedures and coordination, and build team-spirit. Of these clinics, one will serve as the site for piloting the decision-support software, and afterwards, piloting the full intervention. Once piloting is complete, three sites will be selected to participate in Phase 1 (Vanguard Phase) of the trial to assess feasibility (recruitment and retention; continuity of care), to establish procedures, and evaluate and refine multi-site trial coordination. Upon successful completion of this first phase in six months, an additional five sites will join the study (Phase 2).

4.2 Inclusion Criteria

Eligibility criteria for entry into the study include all the following:

1. Age 35 years and older
2. Confirmed diagnosis of diabetes based on documented evidence from oral glucose tolerance test or two venous fasting blood sugar levels or known diabetes patient on medication or insulin (1999 WHO criteria, [154])
3. Poor glycemic control (as evidenced by $HbA_1c \geq 8.0\%$) <u>AND</u> one or both of: dyslipidemia (LDL ≥ 130 mg/dl) or systolic hypertension (SBP ≥ 140 mmHg), irrespective of lipid- or BP-lowering medication use, respectively
4. *Receiving diabetes care in the same clinic for at least 3 months OR even earlier if in the investigator's assessment the patient is likely to follow-up regularly as required by the protocol.*
5. Willingness to consent to randomization

.

4.3 Exclusion Criteria

Individuals will be excluded from participation if **<u>any</u>** of the following are present during screening:

1. Known type 1 diabetes mellitus
2. Diabetes secondary to chronic pancreatitis

3. Pregnant OR trying to become pregnant OR of child-bearing potential and not actively practicing birth control (including natural methods)
4. Evidence of pre-existing well-controlled blood glucose, blood pressure or LDL-cholesterol (as evidenced by HbA1c < 7.0%, SBP < 130 mmHg, LDL-cholesterol < 100 mg/dl [LDL-cholesterol < 70 mg/dl with history of CVD event]) obtained from screening within a period not exceeding 28 days (4 weeks) prior to randomization
5. Documented cardiovascular event (coronary revascularization, stroke, MI, unstable angina) in past 12 months
6. Current symptomatic CHF or NYHA Class 3 or 4 effort intolerance
7. Documented non-diabetic kidney disease OR pre-existing end-stage renal disease (on renal replacement therapy [dialysis or transplant])
8. Transaminase >3 times upper limit of normal OR active liver disease within past 2 years
9. Malignancy or life-threatening disease with death probable in 4 years
10. Any current medication (e.g. long-term steroids, protease inhibitors) that, in the opinion of the site investigator, would interfere with participant's diabetic status and follow-up
11. Any condition or circumstance that is unrelated to diabetes progression, that in the opinion of the site investigator would interfere with the participant's diabetic status and follow-up: including (but not limited to) other endocrinopathy [adrenal, pituitary], TB patient on treatment, psychiatric illness or cognitive impairment, alcohol or drug abuse, history of organ transplant, BMI $\geq 45\ kg/m^2$
12. On an investigational drug in the last 3 months
13. Currently participating in a clinical trial
14. No fixed address or contact details
15. Plans to move in the next 3 years
16. A member of the participant's household is currently in the trial
17. Inability or unwillingness of individual or legal guardian/representative to give written informed consent

4.4 Study Enrollment Procedures

4.4.1 Methods of Recruitment

The recruitment goal is 140 participants at each of the 8 clinic sites for a total sample size of 1,120 participants.

Researchers at each clinic site will identify participants qualified for the trial from clinical records based on the specified inclusion and exclusion criteria (above, Sections 4.2-4.3). Medical record searches or reviews of existing databases can be done initially by setting up the searches using the characteristics that match the inclusion/exclusion criteria. Additional "hand searches" may be necessary using the remaining inclusion/exclusion criteria not already part of the existing database, but that may be ascertained through examining the patient's existing clinical record. It is likely that all or most all of the inclusion/exclusion criteria will be available in most medical records.

Potential participants will be contacted by telephone or approached in-person at the clinic to determine their interest in the study. They will be briefly told about the study, and if interested, they will be invited to the clinic site for 2 pre-randomization visits, for informed consent and screening. If the consenting participant meets eligibility criteria after screening, he/she will be enrolled in the trial (scheduled for complete baseline testing, undergo randomization, and continue with follow-up).

PROTOCOL

4.4.2 Informed Consent

At the first pre-randomization visit, the Site Investigator/Co-Investigator will obtain written, informed consent from all participants who are eligible to participate in the trial. A single participant information sheet and consent form will be used for all the procedures done as part of the screening, baseline testing, and follow-up. We anticipate that most participants will be literate in their local language and/or English; translated forms will be made available in the local languages of all Clinic Sites. See ***Appendix 1*** for the combined **participant information sheet and informed consent** form.

The information sheet and consent form will be given to the potential participant and consent details will be explained, including: purpose of the trial, screening and study procedures, benefits and risks, confidentiality terms, and rights of the participant, and trial contact information. The Site Investigator/Co-Investigator obtaining consent will ask questions to the participant about his/her understanding of what was explained to ensure that the participant correctly understands the PIS-consent information. The individual will be given the opportunity to review the document and any questions or concerns will be addressed. Participation is purely voluntary with no coercion and no material compensation. All participants will be assured that they have the right to voluntarily withdraw from the study at any time, without any repercussions by way of this action affecting their future medical care.

If the individual agrees to participate in the trial, two copies of a signed consent form will be made, with the signatures of the participant and the site investigator. For those who cannot read, the details of the study will be explained to the participant and informed consent will be obtained by the participant's thumb-print and proxy signature by a legally acceptable representative (LAR). In the absence of a LAR, a literate third party (non-study staff) may act as witness. One copy of the consent form will be given to the participant to keep and another copy will be kept in locked storage at each Clinical Site.

These consent procedures will be reviewed and approved by each clinical site's local ethics committee as well as the institutional review boards (IRB) of the participating CARRS institutions. The Data Management and Quality Control Subcommittee will ensure proper collection and storage of the consent forms.

Please see the footnote below for the consent process for physicians being interviewed for evaluation of the trial's ancillary objective: to evaluate the intervention's sustainability and acceptability.[2]

---

[2] **Informed consent for physician interviews:** Signed informed consent will also be obtained from study physicians who will be interviewed at baseline and at annual follow-up about their views on diabetes management and the intervention, to evaluate the secondary objective of trial sustainability and acceptability. The consent form will cover the first and any subsequent interviews. See **Appendix 2** for the combined **physician interview information sheet and consent form**. Trained study staff will recruit the physicians by selective sampling: by contacting (via email/phone/in-person) those physicians known to be involved in the trial. Not more than 3 physicians will be interviewed at each site for each scheduled interview time period (baseline and 3 annual follow-up periods). We anticipate all the physicians to know English; the English information sheet and consent form will be sent to the physician via email to be reviewed.

Before conducting the interview, the trained interviewer will conduct a formal review of the information sheet-consent form with the physician including the purpose of the study, interview procedures, benefits and risks, confidentiality terms, and rights of the participant, and study contact information; any questions will be answered. Participation by the physician in the interview is purely voluntary with no coercion and no material compensation. The physicians will be assured that they have the right to voluntarily withdraw from the interview, without any repercussions by way of this action affecting their involvement in the trial or future practice. Once the physician agrees to participate, the interviewer will obtain the participating physician's signature on two copies of the consent form (one for the physician and the other for local storage). The interviewer will also sign the consent form.

PROTOCOL

4.4.3 **Screening/Baseline Assessment**

After informed consent has been obtained at the first pre-randomization visit, the consented individual will undergo initial screening which will include basic medical history (initial inclusion/exclusion criteria) and blood pressure measurement.

At the end of the first pre-randomization visit, the consented participant will be scheduled for a second pre-randomization visit within approximately 1 week for screening laboratory tests. The participant will be asked to present after fasting for at least 8 hours. A venous blood sample (10 ml) will be taken for blood glucose level, Hemoglobin A1c, lipid profile, blood biochemistries (creatinine, sodium, potassium, ALT) and a urine sample (albumin:creatinine ratio) to determine if the participant meets eligibility criteria.

A screening log will be kept by each Clinical Site to document any reasons for ineligibility and for non-participation of eligible candidates.

Either on the 2nd pre-randomization visit (if lab results are available) or on the 3rd visit, those participants who meet the eligibility criteria will be enrolled in the trial and continue with a further baseline assessment (including a thorough history and physical exam, anthropometric measurements, health-related quality of life and cost of care questionnaires, and preventive screenings) and randomization.

4.4.4 **Randomization**

Restricted randomization of variable blocks will be used for the trial, stratified by the 8 clinic sites. The block allocation sequence for each site (n=140) will be computer-generated centrally (at the Research Coordinating Centre, RCC, in New Delhi), allocating 70 participants to the intervention and 70 participants to standard care. The allocation sequences for each of the 8 sites will be kept in sealed envelopes, stored in a locked space at the RCC at PHFI in New Delhi accessible only to members of the RCC handling trial allocation.

The randomization will be provided centrally using an interactive web response system (IWRS). Once a consented, eligible participant has been confirmed at the Clinic Site (CS), a member of the CS study team will go to the Trial website on the internet and supply the inclusion/exclusion information and will receive a randomization allocation immediately. If the IWRS is not functioning for any reason (malfunction, electricity blackout, etc.), the CS should call the RCC in Delhi for the allocation, which the RCC member overseeing the trial randomization will provide over the phone and by email/fax to the CS study team. The CS study team will be required to send an email/fax confirmation to the RCC of the participant's allocation. Participants will be told at the randomization visit which arm of the trial they have been allocated to.

Concealment of intervention versus standard care allocation to the participants is not possible, due to the transparency of the type of care the patients will receive.

PROTOCOL

5. <u>STUDY INTERVENTIONS</u>

Each Clinic Site will have eligible participants individually randomized to either the intervention or control group. The *intervention group* will receive multi-factorial CVD risk factor control through comprehensive diabetes care supported by a care coordinator and decision-support software. The *control group* will receive usual care provided at the Clinic Site. Physicians treating both groups will be provided with evidence-based management guidelines to ensure fair access to current recommended clinical practices. Refer to **Figure 4** for a summary of the trial groups.

Figure 4: Diagram of Care Received by Intervention and Control Arms

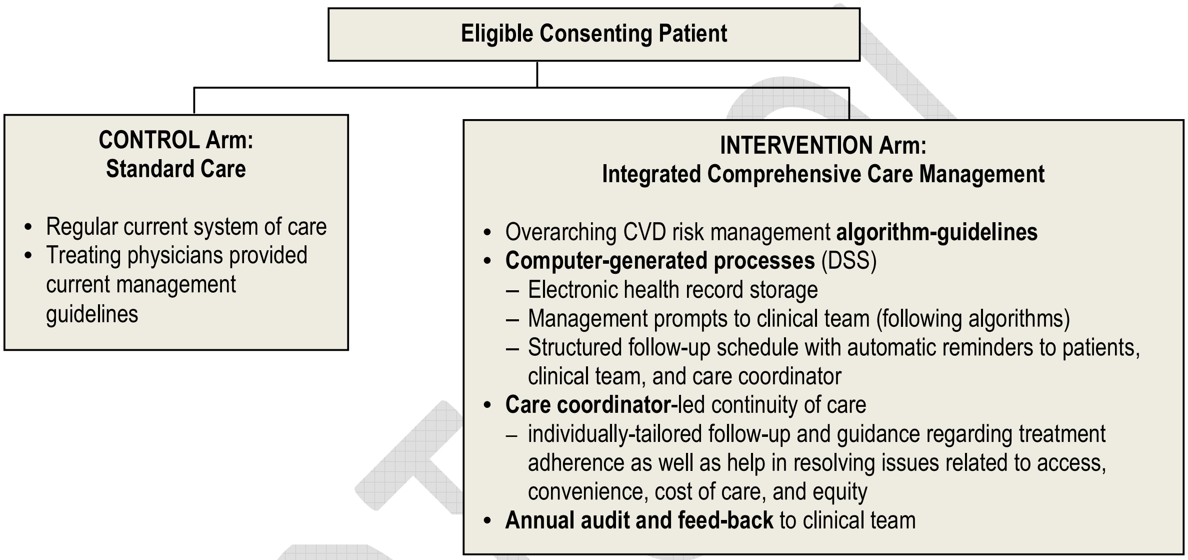

5.1 Intervention Arm

Participants randomized to the intervention group will receive targeted diabetes management through 2 main strategies: 1) a <u>decision-support software</u> (DSS) based on <u>CVD risk management algorithm-guidelines</u> (described below) and 2) support from a <u>non-physician care coordinator</u> who manages the DSS and ensure patient follow-up according to the guidelines.

The <u>CVD risk management algorithm-guidelines</u> (***Appendix 3***) for individuals with diabetes serve as the basis of the intervention's strategies. The evidence-based guidelines provide the clinical team with the up-to-date instruction for intensive CVD risk factor control, targeting regulation of glucose, blood pressure, and lipid control, smoking cessation, and processes of care (aspirin use; ACE-inhibitor use; regular eye, foot, and urine testing). The guidelines follow an algorithm-format and provide progressive recommendations from simple lifestyle management to intensified treatment regimens and referral, according to the participant's status.

The algorithm-guidelines have been developed by a multi-disciplinary team, led by members of the steering committee. The guidelines follow current internationally-accepted evidence, with adaptations to increase relevance to the South Asian population. Feedback from the site investigators has been incorporated into the guidelines.

5.1.1 Decision-Support Software

A simple decision-support software (DSS) will be setup at all clinic sites. The DSS is currently being designed by collaborating programmers in the United States who have a background in creating diabetes management tools.

The web-enhanced DSS will:
(a) store all electronic health records *(EHR component)* and patient habits, integrating all laboratory and consultation reports in one easily accessible location;
(b) provide automated decision-support prompts of guideline-recommended processes of care (e.g. treatment plan, laboratory tests, screenings) tailored to the participant's compliance and CVD risk level; and
(c) send out automatic reminders and alerts to patients, the clinical team, and care coordinator to ensure adherence to the guidelines/ care plan.
(d) serve as a trial data collection and management portal of participant progress and their clinical activities in terms of prescriptions, intensification of therapy, advice given, etc.

The DSS will be managed by the **care coordinator** (see below, *Section 5.1.2*), who will be responsible for enforcing its recommendations at each Clinic Site (CS). Overall, the DSS will organize case management and will assist the care coordinator in following-up with patient care.

5.1.2 Care Coordinators

Recruitment: One non-physician care coordinator will be designated for each Clinic Site for the duration of the trial to facilitate care and follow-up of <u>intervention group</u> patients. Each care coordinator will be recruited locally by the Site Investigator. The study will provide a competitive salary for the care coordinators.

The criteria for care coordinator recruitment are as follows:

1. Registered nurse, social worker, or dietician OR minimum of 12th standard pass and 2 years of health service experience
2. Strong inter-personal, motivational, and organizational skills
3. Willingness to stay for the full duration of the trial (3-3.5 years depending on starting time of the site).
4. Basic computer application knowledge

Responsibilities: Each care coordinator will be managing all 70 participants randomized to the intervention. The work of the care coordinator will be a combination of interactions with the clinical team and personalized follow-up care with participants. The algorithm-based CVD risk management guidelines via the DSS recommendations will be enforced by the care coordinator, complementing the role of physicians. The care coordinator will NOT be allowed to make prescriptive changes for a participant.

Below are the expected functions of the care coordinator:

- Fully manage the DSS, from data-entry of participant information/progress to communication of DSS management prompts to the clinical team;
- Assist the physician to devise a patient-tailored follow-up plan of participants based on their compliance and motivation and other CVD risk factors. Patients who are not compliant or at high-risk will require more intensive follow-up (e.g. home-visits and monitoring; motivation to improve lifestyle, treatment adherence etc.) while more compliant participants might require less intensive follow-up (group visits at the clinic with information sessions).

PROTOCOL

- Facilitate follow-up, referral, treatment, and investigation appointments including reminders to enforce participant's care plan (achieving target BP, lipid, and glucose control; use of aspirin and RAS-modifier therapies; lifestyle modification and smoking cessation; screenings);
- Encourage and motivate participants to better self-manage risk factors (by providing the appropriate guidance and tools), promote better lifestyle choices (physical activity, diet, smoking cessation) and treatment adherence;
- Prompt earlier attention of patient's needs/progress to the treating physician (e.g. eliciting a prescription without waiting for the patient's next clinic appointment);
- Arrange regular meetings with the clinical team to review patient progress, address patient needs, and other trial issues;
- Resolve issues of access, convenience, and cost of care, and equity; and
- Manage the intervention group's trial data collection, documentation and communication with the Research Coordinating Centre (RCC)

Training of care coordinators will occur in two stages prior to the start of the trial. A standardized instruction course for the full group of hired care coordinators will be held at a selected training center in India, followed by individual training at each Clinic Site to contextualize duties in setting and with the clinical team.

The work of the care coordinators will be routinely monitored by the RCC to document their progress and quality of care delivered by the physician- care coordinator team. Routine feedback will be provided to care coordinators to improve areas of weakness by the Site Investigator. Formal evaluation of care coordinators will occur at selected testing time points during the trial (See *Section 10.3 Quality Assurance*).

To ensure proper execution of the intervention, **annual audit and feedback** will be provided by the RCC to all the Clinical Site teams regarding their site's progress and areas for improvement (See *Section 10.3 Quality Assurance*).

PROTOCOL

5.2 Control Arm

Participants randomized to the control arm will receive the existing standard care and treatment for their diabetes that is provided routinely at each Clinic Site. As mentioned above, the physicians treating the control arm will also be provided with the CVD risk management guidelines to ensure fair access to current recommended clinical practices. The control participants will have no contact with study staff, other than during study follow-up visits.

Either a clinic staff or a hired research assistant will serve as the **research officer** to assist with participant randomization and manage the control group's study-related visits and data collection – separate from the duties of the care coordinator with the intervention group.

5.2.1 **Research Officer**

Recruitment: The part-time position of research officer will be recruited locally by the Site Investigator. A monetary compensation will have to be arranged within the budget allotted to each Clinic Site for the trial.

The criteria for the research officer are as follows:
1. Minimum of 12th standard pass
2. Willingness to stay for the full duration of the trial (3-3.5 years depending on starting time of the site)
3. Basic computer application knowledge

Responsibilities: The research officer at each clinic will be recording data collected on the 70 participants randomized to the control group into the electronic health record system.

The following are the expected functions of the research officer:

- Assist with baseline assessment and randomization of participants.
- Collect study-visit and any non-study visit data of the control group participants using paper Case Report forms (CRFs). Data collection during non-study visits will be done AFTER the physician sees the participant (uni-directional flow of information – he/she will NOT feedback anything additional about the participant to the physician and vice-versa)
- Will have access to only the electronic health records aspect of the DSS (only the EHR, no decision-support) for his/her CONTROL arm patients only for data entry on a regular basis (once every 2 weeks)
- Must not advise participants or clinical staff member in any way
- Must NOT follow-up with participants
- Must NOT arrange appointments or laboratory tests for participants other than the required annual study-visit
- Must NOT substitute for care coordinator when he/she is on leave

Like the care coordinator, the research officer will be trained on site prior to the trial and during the site orientation with the clinic team.

Like the care coordinator, the work of the research officer will be routinely monitored by the RCC to ensure proper data collection. The Site Investigator is also responsible for overseeing the Research Officers' performance. Formal evaluation of the research officers will occur at selected testing time points during the trial (See *Section 10.3 Quality Assurance*).

Detailed instruction of the trial interventions described above will be contained in the *Manual of Procedures*.

6. <u>STUDY PROCEDURES</u>

Please see the next page.

PROTOCOL

6.1 Schedule of Evaluations (green-highlighted columns are study data-collection visits)

Forms: A - Screening Part 1; B - Screening Part 2; C –Baseline_Randomization; D –3 monthly Visit_Intervention; E – Follow-up 12-monthly_All; F – Close-out_All; G – Eye Exam; I.c – Intermediate Visit_Control; I.i -Intermediate Visit_Intervention; Z –Intervention Management Plan; K - Interview Guide_Physician

PHASE	**Screening Part 1**	**Screening Part 2**	**Baseline/ Randomization**	**3-monthly Visits (Intervention Group)**	**Intermediate Visits**		**Follow-up 12-monthly**	**Close-Out**
**FORM**	A	B	C + G, K^	Intervention: D, Z	Intervention: I.i, Z	Control: I.c	E + G (Intervention: Z), K^	F + G (Intervention: Z), K^
MONTH	Max -4 weeks	-4 to 0 weeks	0	3, 6, 9, 15, 18, 21, 27, 30, 33, 39	Other than 3-monthly	As occurs (after visit-records)	12, 24, 36	(42/last visit)
Signed informed consent	X							
Assess Eligibility	X	X	X					
Enrollment/Randomization			X					
Demographics			X					
Medical history	X (basic)		X (full)	X (interval)	X (interval)		X (interval)	X (interval)
Family history			X					
Social History (lifestyle/habits)			X				X	X
Self-care (SDSCA)			X	X (partial)	X (partial)		X	X
Quality of Life, short (EQ5D)			X				X	X
Diabetes Treatment Satisfaction (DTSQ)			X				X	X
General Quality of Life (HUI-3)			X				X	X
Costs of Care (records, questionnaire)			X				X	X
^QA/Acceptability: physician interviews			Subsample				Follow-up subsample	Follow-up subsample
Blood pressure (BP) and heart rate	X (BP only)	X		X	X		X	X
Height		X					X	X
Weight, BMI (calculated), Waist Circumference		X		X	X		X	X
General Physical Examination			X				X	X
Eye Examination			X				X	X
Foot Examination			X				X	X
ECG			X				X	X
Albumin:creatinine ratio (urine analysis)			X				X	X
*Serum Creatinine		X					X	X
*Serum Sodium, Potassium and ALT		X						
Venous fasting blood glucose (FBG)		X		X	X		X	X
Hemoglobin A1c		X		X			X	X
Lipids (TC, HDL, LDL [calculated], TG)		X					X	X
Glucose lowering, blood pressure lowering, and cholesterol lowering medications			X	X	X		X	X
Other concomitant medications			X	X	X		X	X
Visit Summary & Management			X	X	X	X	X	X
AE/ SAE Intake (ongoing) – Form X			X	X	X	X	X	X

*Follow-up serum potassium and creatinine levels if high and started on ACE-i; follow-up ALT if started on statin. ^The interviews of physician sub-samples (Form K) will be administered by RCC staff.

6.2 Description of Evaluation Schedule

The first 2 visits are for participant screening. The 3rd visit is for final screening, further baseline assessments and randomization.

Guideline-recommended follow-up for all participants is on at least a 3-monthly basis and more frequently if the participant has poorly controlled diabetes. The intervention and control groups will have annual follow-up study visits to capture outcomes and complications. All other intermediate visits by the participants will be documented.

For all randomized participants, the average post-randomization follow-up period will be 30 months (2.5 years). All follow-up 12 monthly visits must occur within ± 2 weeks of the scheduled follow-up visit date. If the visit happens outside this window, the Site Investigator should proceed with the visit but indicate a note in the file stating 'protocol violation.'

The study staff at each Clinical Site involved in executing the study procedures includes:

- **Site investigator/co-investigator/sub-investigator:**
    - Oversee all trial-related procedures (recruitment, screening, enrollment, randomization, and follow-up of participants)

- **Study physician (includes Site Investigator/co-investigator/sub-investigator):**
    - Serves as **Screening officer**: Perform screening evaluations, baseline history and physical examination and conducts randomization of participant into trial.
    - Assist in intervention and control group study visits (details below)
    - If seeing an intervention participant, work as a team with the care coordinator to manage the participant's care

- **Care coordinator:**
    - Perform recruitment of participants, but no interaction during screening evaluations
    - Coordinate with site-investigator/co-investigator and study physician(s) to complete all follow-up visit requirements; ensure complete data collection for intervention group
    - Data collection for control group on paper CRFs
    - Enter intervention group's data into the eCRFs of the DSS
    - Supply Intervention Management Plan (Form Z) to study physician to finalize care plan at each visit
    - Other details below

- **Research officer:**
    - Perform recruitment of participants, but no interaction during screening evaluations
    - Data collection for control group on paper CRFs, minimal interaction during follow-up to prevent any change in the standard care
    - Enter control group's data into the eCRFs of the DSS
    - Other details below

*All **case report FORMS** are provided in alphabetical order in the final appendix (**Appendix 5**) and included as separate documents in the electronic version. Detailed instructions of evaluation schedule are found in the MOP.*

PROTOCOL

6.2.1 Screening (Pre-randomization)

- **Screening Part 1 (maximum -4 weeks): all potential participants**
  Form: **Form A - Screening Part 1**
  Participant Log

  Potentially eligible patients will visit the Clinic Site for the first pre-randomization visit for informed consent by the **Site investigator/co-investigator** (*Section 4.4.2* for details) and Part 1 of screening by **screening officer**, which includes:
  - Contact information
  - Basic medical history through General Inclusion/Exclusion Criteria
  - Blood pressure measurement
  - Assess eligibility - report in Participant Log

  The patients who do not meet eligibility criteria for the trial will be notified of their status. The patients who are still eligible for the study after this 1st pre-randomization visit will be asked to present fasting (8 hours) for screening laboratory tests (2nd pre-randomization visit) and Screening Part 2 (see below).

- **Screening Part 2 [laboratory tests& assessment] (-4 to 0 weeks): all potential participants**
  Form: **Form B - Screening Part 2**
  Participant Log

  Within 2 week after Screening Part 1(Form A), the participant will present to the clinic in a fasting state for the 2nd pre-randomization visit for screening laboratory tests, which include:
  - Urine sample for urine albumin:creatinine ratio
  - Blood sample for measurement of Hemoglobin A1c and blood chemistries (serum creatinine, sodium, potassium, and ALT) to determine an existing medical condition in the study exclusion criteria and for medication effects
  - Fasting venous blood sample for glucose and lipids (triglycerides, total cholesterol, HDL cholesterol, and LDL [calculated])

  Either on the same 2nd pre-randomization visit for if lab results are available the same day *OR* on a 3rd visit within 4 weeks after Screening Part 1, the **screening officer** will complete Form B (Screening Part 2) with the participant, which includes:
  - Blood pressure and heart rate
  - Height, weight, BMI [calculated], waist circumference
  - Review of lab test results
  - Assess eligibility - report in Participant Log

  Those potential participants who do not meet eligibility criteria will be notified of their status at the clinic. Those individuals still meeting the inclusion/exclusion criteria will continue with the baseline evaluation and randomization (see Section 6.2.2).

PROTOCOL

6.2.2 Baseline and Randomization: ALL eligible participants

Forms: **Form C – Baseline_Randomization; Form G- Eye Exam**
Form K– Interview Guide_Physician
Participant Log

After Screening Parts 1 and 2 have been completed, the eligible patient will undergo the baseline evaluation and randomization (maximum of 4 weeks after the first screening visit) by **screening officer**. This can occur the same day when Screening Part 2 is completed.

For **all participants**, the **screening officer** will:

- Complete **Form C – Baseline_Randomization (Part 1)** which includes
    - Medical, family history and medications
    - Make any necessary changes in management
    - Complication screenings: ECG, foot exam, eye exam via **Form G- Eye Exam** (to be completed by qualified ophthalmologist)
- **Randomize** the eligible participants using the interactive web response system (IWSRS). See MOP for instructions. Report enrollment and allocation in Participant Log.

For **control arm** (standard care) participants, the **research officer** will:

- Complete **Form C – Baseline_Randomization (Part 2)** which includes: demographics, social/lifestyle habits, and questionnaires (self-care [SDSCA], quality of life [EQ5D and HUI-3], diabetes treatment satisfaction [DTSQ], and frequency/costs of care).
- Advise the participant that his/her diabetic care will remain unchanged at present; he/she will be expected to see the usual physician at the frequency he/she advises. He/she must be available to return once a year for a study-related visit.
- Enroll the patient for the trial in the DSS, and complete the eCRFs for Forms B and C.

For **intervention arm** participants, the **care coordinator** will:

- Complete **Form C – Baseline_Randomization (Part 2)** which includes: demographics, social/lifestyle habits and questionnaires (self-care [SDSCA], quality of life [EQ5D and HUI-3], diabetes treatment satisfaction [DTSQ], and frequency/costs of care).
- Advise the participant that their diabetic care will be intensified and he/she will have regular follow-up from the care coordinator and be asked to visit the clinic at least every 3 months. Enroll the patient for the trial in the DSS, and complete the eCRFs for Forms B and C.
- The care coordinator will facilitate the participant's adherence to appointments and self-management using the DSS-generated reminders and phone calls.

Qualitative Assessment: A maximum of 3 study physicians (mainly Site Investigator/ Co-Investigators) at each clinic site will be consented and interviewed at the site initiation visit by RCC monitor to have an initial understanding of their practices and views on the intervention. These interviews of not more than 30 minutes will be conducted following written guides **(Form K – Interview guide_Physician)**. See *Section 9.6.3 Qualitative Data Analysis* for more detail.

PROTOCOL

6.2.3 Follow-up 12 monthly Visits (+12, +24, and +36 months): ALL participants

Forms: **Form E – Follow-up 12 monthly_All; Eye Exam Form**
Form Z – Intervention Management Plan
Form K – Interview Guide_Physician

All participants (intervention and control) will present for the 12-monthly follow-up visits. All participants will have to obtain all investigations: venous FBG, HbA1c, lipid profile, serum creatinine and other blood biochemistries (serum sodium, potassium, ALT) if necessary, and complication screenings (urine albumin:creatinine ratio, eye exam, and ECG) prior to the visit. All participants will be required to present the lab results at the visit, if the Clinic Site does not have a system in place to obtain the laboratory results independently.

- The control arm will follow the usual process for setting up laboratory appointments.
- For the intervention arm, one week prior to the visit, the care coordinator will ensure that the intervention participants have obtained the laboratory tests for the visit.

Prior to the physician assessment, the **care coordinator/research officer** will obtain the following data from the participants for **Form E (Part 1)**:

- Interval history, hypoglycemic events
- Measures: Blood pressure and heart rate, height, weight, BMI [calculated], waist circumference
- Investigations: values from lab reports; Reports of ECG and Eye Exam **(Form G)**
- Social/lifestyle habits and questionnaires: self-care [SDSCA], quality of life [EQ5D and HUI-3], diabetes treatment satisfaction [DTSQ], and frequency/costs of care

For the **control arm:**

- After the **research officer** completes **Form E (Part 1)**, the study physician will see the participant and complete **Form E (Part 2)** (Complications Screenings, Medical history, Adverse and Serious Adverse Events, Physical Exam, and medications update) and fix the next visit date.
- The research officer will input **Form E** in the DSS.

For the **intervention arm:**

- The **care coordinator** will input the specified data from **Form E (Part 1)** into the DSS and print out an **Intervention Management Plan (Form Z)**. The care coordinator will update the patient's medications in Form Z, and provide Form Z to the physician during consultation.
- The **study physician** will complete **Form E (Part 2)** and review **Form Z** and accept/reject the management prompts, update medications and the patient management plan; fix next visit date and review the plan with the care coordinator.
- The care coordinator will update the patient; address any barriers to care and motivate him/her to achieve risk factor management goals and confirm the next appointment dates. The care coordinator will input the updated Form Z and rest of Form E in the DSS.
- The care coordinator will facilitate the participant's adherence to appointments and self-management using the DSS-generated reminders and phone calls.

Qualitative Assessment: The same sample of interviewed physicians at each site will be interviewed during the following 2 annual monitoring visits by the RCC monitor to assess changes in practice and feedback on the intervention. The interviews of maximum 30 minutes will be conducted by a trained research staff using written guides (**Form K**). See *Section 9.6.3 Qualitative Data Analysis* for more detail.

PROTOCOL

6.2.4 **3-monthly and Intermediate Visits**

- **3-monthly Visits (+3, +6, +9, +15, +18, +21, +27, +30, +33, +39 months): Intervention Arm only**

  Forms: **Form D – 3 monthly Visit_Intervention**
  Form Z –Intervention Management Plan

  For the **intervention arm only**:
  - The **care coordinator** ensures that intervention patients visit the clinic for 3-monthly visits.
  - One week prior to the visit, the **care coordinator** will ensure that the participant has obtained the following laboratory tests for the visit: HbA1c, venous fasting blood glucose, and any other lab tests that the physician had specified at the previous visit
  - Laboratory reports will be collected on the day of the visit by the care coordinator.
  - At the visit, the **care coordinator** will complete **Form D–3 monthly Visit_Intervention** with the following:
    - Interval history, hypoglycemic events, adverse events, self-care
    - Measures: blood pressure, heart rate, weight, BMI (calculated with baseline/annual height) and waist circumference
    - Investigations: values from lab reports (HbA1c, venous fasting blood glucose and other tests ordered)
  - The **care coordinator** will input **Form D** into the DSS and print out an **Intervention Management Plan (Form Z)**. The care coordinator will update the patient's medications in Form Z, and provide Form Z to the physician during consultation.
  - The **study physician** will review **Form Z** and accept/reject the management prompts, update medications and the patient management plan; fix next visit date and review the plan with the care coordinator.
  - The care coordinator will update the patient; address any barriers to care and motivate him/her to achieve risk factor management goals and confirm the next appointment dates. The care coordinator will input the updated Form Z in the DSS.
  - The care coordinator will facilitate the participant's adherence to appointments and self-management using the DSS-generated reminders and phone calls.

- **Intermediate Visits**

  For Control arm: clinic visits other than 12-monthly visits
  For Intervention arm: clinic visits other than 3-monthly and 12-monthly visits

  Forms: **Form I.c - Intermediate Visit_Control**
  Form I.i - Intermediate Visit_Intervention
  Form Z – Intervention Management Form

  The research officer and the care coordinator will need to document all intermediate visits for the control and the intervention arm participants, respectively.

  For the **control arm**:
  - The control participant's file will be tagged, so that whenever the patient returns for a visit, a clinic staff should notify the research officer that the patient has arrived.
  - After the patient's visit is complete, the research officer will need to fill out **Form I.c – Intermediate Visit_Control** only by looking at the patient's file.

- The research officer will input **Form I.c** in the DSS.
- The research officer does NOT contact the control patient to schedule appointments or ensure regular follow-up. The follow-up routine will continue as per existing care at the clinic.

For the **intervention arm:**

- For all other clinic visits outside the 3-monthly and 12-monthly visits, the **care coordinator** ensures patient appointments, documentation and follow-up of all intermediate visits.
- One week prior to the visit, the **care coordinator** will ensure that the participant has obtained the following laboratory tests for the visit: venous fasting blood glucose and any other lab tests that the physician had specified at the previous visit.
- Laboratory reports will be collected on the day of the visit by the care coordinator.
- At the visit, the **care coordinator** will complete **Form I.i – Intermediate Visit_Intervention** with the following:
    - Interval history, hypoglycemic events, adverse events, self-care
    - Measures: blood pressure, heart rate, weight, BMI (calculated with baseline/annual height) and waist circumference
    - Investigations: values from lab reports (venous fasting blood glucose and other tests ordered). List the last HbA1c value from the previous 3-monthly or 12-monthly visit.
- The **care coordinator** will input **Form I.i** into the DSS and print out an **Intervention Management Plan (Form Z)**. The care coordinator will update the patient's medications in Form Z, and provide Form Z to the physician during consultation.
- The **study physician** will review **Form Z** and accept/reject the management prompts, update medications and the patient management plan; fix next visit date and review the plan with the care coordinator.
- The care coordinator will update the patient; address any barriers to care and motivate him/her to achieve risk factor management goals and confirm the next appointment dates. The care coordinator will input the updated Form Z in the DSS.
- The care coordinator will facilitate the participant's adherence to appointments and self-management using the DSS-generated reminders and phone calls.

6.2.5 Completion/Final Evaluation

Forms: **Form F – Close-Out_All; G- Eye Exam**
Form Z – Intervention Management Plan
Form K – Interview Guide_Physician

Scheduling:

(1) If the last 12-monthly visit for a participant is scheduled for within 3 months from the last date of the trial (December 31, 2013), then the last 12-monthly visit becomes the close-out visit.
(2) If the last 12-monthly visit for a participant is/was scheduled for more than 3 months from the last date of the trial (December 31, 2013), then conduct a close-out visit by the last trial date. For an intervention patient, a scheduled intermediate visit within 3 months from the last trial date becomes the close-out visit.

The close-out visit process is exactly the same as the 12-monthly visit, except Form F is used in place of Form E. Please refer to Section 6.2.3 for details. For intervention patients, Form F will contain questions on acceptability of the intervention; updates to Form F will be sent to the IRB for review/approval prior to its use.

Final Qualitative Assessment: The physicians interviewed previously will be undergo a final interview with the RCC monitor at the trial closing visit for feedback on the intervention and changes in their clinical practice. These interviews of maximum 30 minutes will be conducted by a trained research staff using written guides (**Form K**). See *Section 9.6.3 Qualitative Data Analysis* for more detail.

Follow-up on participants once they have stopped participation in the study intervention group will be conducted for monitoring and reporting any adverse experiences or outcomes in the first year after discontinuing participation in the trial.

Potential reasons for early termination can be found in *Section 8: Intervention Discontinuation*.

PROTOCOL

7. SAFETY ASSESSMENTS

7.1 Specification of Safety Parameters

The CVD risk reduction delivery strategies of the intervention are not implementing any new drug or invasive procedure that requires specific monitoring of safety parameters. Rather, the intervention is a preventive health study that is enforcing existing, evidence-based guidelines for CVD risk reduction. The control group will follow the existing standard care provided at the clinic site, while the intervention is designed to track quality of care delivery, and achieve risk factor control (via regular examination of values of Hemoglobin A1c, fasting blood glucose, cholesterol and triglycerides, blood pressure, heart rate, and BMI, and implementation of more intensive preventive measures for any high range values).

Adverse events and serious adverse events will be monitored.

7.2 Adverse Events and Serious Adverse Events

Definition of an Adverse Event: "An **adverse event (AE)** is *any untoward medical occurrence in a subject temporally associated with participation in the clinical study or with use of the experimental agent being studied*. An adverse finding can include a sign, symptom, abnormal assessment (laboratory test value, vital signs, electrocardiogram finding, etc.), or any combination of these."

List of Adverse Events:

1. Mild hypoglycemia not requiring medical attention
2. Side-effects of medications (e.g. hepatic dysfunction or myopathy due to statins; dry cough due to ACE-I; negative effects of drugs on biochemical parameters such as hypo- or hyper- kalemia, hyperuricemia; and others per Investigator discretion)
3. PVD: intermittent claudication, rest pain
4. Allergic reactions/reactions on basis of drug interactions
5. Infection (UTI, skin infections, soft tissue infections, lower respiratory tract infections/physician-diagnosed pneumonia)
6. Weight gain

Definition of a Serious Adverse Event: "A **serious adverse event (SAE)** is any adverse event (*any untoward medical occurrence in a subject temporally associated with participation in the clinical study or with use of the experimental agent being studied*) that results in one or more of the following outcomes: Death; a life-threatening event; inpatient hospitalization or prolongation of existing hospitalization; a persistent or significant disability/incapacity; a congenital anomaly or birth defect; or an important medical event based upon appropriate medical judgment."

List of Serious Adverse Events:

1. Severe hypoglycemia requiring medical attention/hospitalization (a hypoglycemic episode associated with transient central nervous system dysfunction without other apparent cause in which the individual was unable to treat him/herself and had help from another person to administer glucose or glucagon)
2. Acute hyperglycemia (e.g. diabetic ketoacidosis)
3. CVD events: Angina, non-fatal MI / Unstable Angina, revascularization procedure [angioplasty or CABG], TIA, Stroke (non-fatal), Arrhythmia
4. Gangrene or amputation due to diabetes-related peripheral neuropathy and peripheral vascular disease
5. Major bleeding (e.g. intracerebral or gastro-intestinal)

PROTOCOL

6. Renal: end-stage renal disease requiring renal replacement therapy (dialysis or transplantation)
7. Eyes: severe diabetes-related eye disease (defined as the requirement for retinal photocoagulation or similar treatment and development of diabetes-related blindness in either eye in a participant known not to have this condition at study entry)
8. Major infection: requiring hospitalization and/or parenteral antibiotics (UTI, skin infections, soft tissue infections, lower respiratory tract infections/physician-diagnosed pneumonia)
9. Inpatient hospitalization or prolongation of existing hospitalization
10. Death
11. Any other major health conditions/events (important medical event based upon appropriate medical judgment)

Classification of AE Severity: AEs will be labeled according to severity, which is based on their impact on the patient. An AE will be termed 'mild' if it does not have a major impact on the patient, 'moderate' if it causes the patient some minor inconvenience and 'severe' if it causes a substantial disruption of the patient's well being.

AE Attribution Scale: AEs will be categorized according to the likelihood that they are related to the study intervention. Specifically, they will be labeled either definitely, probably, possibly, or unrelated to the study intervention.

Expected Risks: We judge the potential risks from data collection to be minimal. No invasive procedures are under consideration, except for collection of venous and capillary blood samples; these procedures are minimally invasive. However, there is a risk of bruising or discomfort with blood draws and, very rarely, the procedure can result in inflammation or infection of the arm veins. It is also unlikely, although possible that trial instrument questions may cause distress to the participant. Risks are considered minimal, and participants will be informed of all possible risks in the consent form. If the participant deems the risks too large, he or she may refuse to take part in the study or withdraw at any time.

Measures to Minimize Risk: Appropriate precautions will be taken to avoid inflicting harm or risk to the well being of the subjects. Any potential adverse effects will be monitored and reported by the study investigators immediately to Emory University's Institutional Review Board (IRB) and the PHFI Institutional Ethics Committee (IEC) for appropriate action. All members of the study team are CITI certified. Even though the potential for injury to research subjects due to the risks of the proposed procedures is judged to be minimal, all reasonable efforts will be made to minimize these risks through the exclusive use of properly trained and educated research personnel. Individuals who are injured by study procedures will be informed of their rights to and be offered treatment of the injury as part of the informed consent procedure and the prevailing local health policy. Specifically:

- To minimize risks associated with blood draws, the study team will include experienced and trained phlebotomists.
- Participants will be informed of their right to refuse to answer any survey question that makes them uncomfortable.
- Any undiagnosed disease or condition or abnormal test results that present during study testing will be brought to the attention of the Principal Investigator and the participant.
- The patient will be referred for emergency care for emergencies or to their personal physician or a community hospital or clinic if he or she does not have a personal physician for non-emergencies.
- Where mild injury / discomfort is caused to the patient, e.g. vasovagal attacks, needle-stick injury, etc., appropriate procedures will be followed to attend to this by study and medical staff

PROTOCOL

7.3 Reporting Procedures

Information about the occurrence of any AEs or SAEs will be sought at all scheduled visits. When a SAE occurs, the responsible Site Investigator should ensure that the SAE is reported within 24 hours to the RCC by completing a *Serious Adverse Event Form* (See **Form X - Serious Adverse Event** in ***Appendix 5***), sent by fax, email or online submission and with notification by telephone. The Clinical Site must report the SAE to the local ethics committee within 7 days. The RCC will report SAEs that are unanticipated or possibly related to the study intervention to the DSMB and the PHFI IEC within 15 calendar days, with appropriate follow-up reports and final resolution forms with supporting documents as per evolution of the disease. Anticipated SAEs or those unrelated to the study intervention will be reported to the same individuals/entities on a monthly basis.

Serious adverse events that occur within 15 days after the end of the scheduled follow-up visit will be reported in the same way as those that occur before the end of follow-up. In addition, any adverse event that occurs after the completion of the scheduled follow-up, and that the investigator deems due to the study intervention will be reported in the same way.

The Site Investigator will ensure that there is adequate follow-up of each participant who has a serious adverse event. Also, the Site Investigator should ensure that all regulatory requirements specified by the local IRB are completed. The Data and Safety Monitoring Board will regularly review all such events (*See below Section 7.4 Safety Monitoring*) and provide recommendations to the CARRS Trial Investigators. Documentation of all such SAEs will be retained in the participant's trial folder for at least 3 years.

7.4 Safety Monitoring

This is a translation trial of CVD risk reduction delivery strategy with no direct risks anticipated for participants. Site monitoring visits will occur on at least 3 occasions in the first year and on at least two occasions each year thereafter. Sites will provide 3-monthly progress reports with information on participant recruitment/retention, adverse events, and any protocol deviations or issues. The Quality Control Subcommittee will review the reports from the site monitoring visits and the site progress reports every 6 months. The Quality Control Subcommittee will provide an annual report for the DSMB, PHFI IEC, the Steering Committee and other applicable recipients who will review progress of this study on an annual basis and provide recommendations, as necessary.

The report will include:

1. A list and summarization of adverse events
2. Whether adverse event rates are consistent with pre-study assumptions;
3. Reason for dropouts from the study;
4. Enrollment of participants, by site, age, gender, other characteristics
5. Whether all participants met entry criteria;
6. Whether continuation of the study is justified on the basis that additional data are needed to accomplish the stated aims of the study; and
7. Conditions whereby the study might be terminated prematurely.

The DSMB will convene annually to review the report and other issues pertaining to the trial's progress and safety. The annual report will be signed by the DSMB chairperson and forwarded to the PHFI IEC and the CARRS Steering Committee of Investigators. Minutes of the DSMB recommendations will be provided to NHLBI within 30 days of convening.

PROTOCOL

8. INTERVENTION DISCONTINUATION

Criteria for discontinuing the intervention **for a participant** include:

1. Moves away from proximity of Clinic Site
2. If the Site Investigator finds the participant to be incompatible with the intervention

If any member of the local study team encounters a participant that meets any of the criteria for discontinuation, he/she must inform the Site Investigator, who will report the incident to the RCC by completing a Participant Discontinuation Form. The decision of participant discontinuation will be reported to the required entities.

Reasons for discontinuation of the study intervention itself **at a Clinic Site** include:

1. Infrastructure unable to handle intervention (determined by quality monitoring).
2. Discontinuation recommended by steering committee or sponsor
3. Low-recruitment rate: less than 8 patients per month recruited

Participants will continue to be followed with their permission if the study intervention is discontinued at the Clinic Site.

These participants will be followed up annually for 3 years for adverse events and serious adverse events/clinical outcomes of (1) death from any cause; (2) major macrovascular event: a composite of non-fatal MI, non-fatal stroke and death from any cardiovascular cause (based on investigator diagnosis).

Secondary outcomes which will be followed include:
(1) MI (non-fatal and fatal or revascularization procedure [angioplasty or CABG]);
(2) stroke (non-fatal and fatal);
(3) requirement for renal replacement therapy (dialysis or transplantation);
(4) death from renal disease;
(5) development of severe diabetes-related eye disease (defined as the requirement for retinal photocoagulation or similar treatment and development of diabetes-related blindness in either eye in a participant known not to have this condition at study entry); and
(6) major hypoglycemia episode (a hypoglycemic episode associated with transient central nervous system dysfunction without other apparent cause in which the individual was unable to treat him/herself and had help from another person to administer glucose or glucagon)
(7) Acute hyperglycemia (e.g. DKA)
(8) Amputation due to diabetes-related peripheral neuropathy and peripheral vascular disease
(9) Major infection requiring hospitalization (e.g. pneumonia)
(10) Any other health conditions/events

PROTOCOL

9. STATISTICAL CONSIDERATIONS

9.1 General Design Issues

The study design is a controlled, parallel group, multi-site translation trial to test the effectiveness of a multi-factorial CVD risk reduction intervention using clinical decision-support software and non-physician care coordinators, in Type 2 diabetes patients attending established out-patient clinics in South Asia, compared to the control group receiving standard care; for differences in the primary outcome of *achieving* multiple risk factor control targets.

Hypothesis: Compared to the control group, the intervention group will, on average, demonstrate a relative 40% greater proportion of participants achieving multiple CVD risk factor control targets (at least two targets including HbA1c < 7.0% AND at least one of SBP < 130 mmHg or LDL-cholesterol < 100 mg/dl).

Secondary outcomes include single CVD risk factor control targets, quality of care measures, and participant's health-related quality of life and treatment satisfaction.

An ancillary objective is assessing the sustainability of the intervention by determining the cost-effectiveness of the intervention vs. standard care and assessing patient and provider perspectives regarding the intervention's acceptability and effectiveness.

9.2 Sample Size and Randomization

In keeping with the theme of delivering comprehensive, multi-factorial CVD risk management to diabetes patients, this translation trial is powered on between-group differences in proportions achieving multiple risk factor targets (reflected by intermediate biochemical measures). Although there is great interest in and significance attached to improvements in the extent and regularity of preventative screening and treatment processes, these processes do not always mirror improvements in outcomes.[98]

Therefore, the trial is specifically powered for intermediate outcomes (i.e., multiple risk factor control). Sample size calculations are predicated on documented low proportions of sample populations reaching individual and multiple risk factor target goals,[89, 92] 20% allowance for loss to follow up and/or missing data, and 80% power to detect conservative minimum between-group differences in improvement (see Table 1). Calculations were performed using OpenEpi Software.[155, 156]

Table 1: Sample Size Calculations for CARRS Translation Trial

Power	Alpha	Risk Factor	Proportion of Control Group achieving	Proportion of Intervention Group achieving	Total Sample Size Required	20% additional-loss to follow up &/or missing data
		**Primary Outcome**				
80%	0.05	Multiple (≥2 risk factors controlled)	20%	28%	896 † - 942 *	1,076 – 1,131
90%	0.05	Multiple (≥2 risk factors controlled)	20%	30%	832 *	999
		**Secondary Outcomes**				
80%	0.05	Glycemia (HbA_1c)	37%	47%	766 †	920
		BP (mmHg)	36%	46%	772 †	927
		Lipid (TC; mg/dl)	48%	58%	774 †	929
90%	0.05	Glyc	37%	47%	1,032 †	
		BP	36%	46%	1,024 †	
		Lipid	48%	58%	1,034 †	

† Kelsey et al. Methods in Observational Epidemiology 2nd Edition, Table 12-15; * Fleiss with Continuity Correction

PROTOCOL

The primary outcome was constructed based on delivering care that improves global CVD risk control in participants with poor baseline control. A minimum n=1076 is required; an enhanced sample of 1,120 subjects will be studied to enable demonstration of a 40% relative difference in proportions attaining multiple clinical treatment goals between the intervention and control groups.

Our sample size also permits us to demonstrate reproducible and achievable secondary outcomes (10% point increases in participants reaching individual risk factor targets) in the intervention group. Although the study has not been powered for CVD event and/or mortality reduction, we anticipate that the data we collect will be useful for putatively planning an extension of this trial to detect distal clinically significant CVD and/or mortality endpoints. In addition, we will assess differences in quality of life and patient satisfaction between participants randomized to the intervention and standard care.

We aim to have 140 participants randomized (70 in the intervention arm, 70 controls) at a total of 8 clinic sites, amounting to 1,120 trial participants. The initial vanguard phase will occur at three sites (n=420), and another five sites (n=700) will be added 6 months later.

Careful consideration of the following points led to the choice of individual over cluster randomization:

- Bias will be problematic in cluster randomization, and in particular, since quality of care is being assessed simultaneously, it is unlikely that health care providers at different sites will cooperate to being controls (they are likely to refuse consent) as this will undermine their practices.
- The intervention is multi-faceted (structured guidelines, decision support system, reminders, care coordinator) and these elements will be tailored, based on risk stratification, to suit the needs of the individual. Therefore, there is the potential for heterogeneity in packages of care in individuals who see the same care coordinator. Group outcomes will therefore be dissimilar and not comparable.
- Outcomes (achieving risk factor control targets, quality of care indicators, quality of life and any unforeseen incident events, morbidity and mortality) are all measured at the individual level.

Individual randomization, in relative terms, has benefit with respect to the sample size and power derived from this design over cluster randomization, and will minimize bias.

9.3 Interim Analyses and Stopping Rules

Interim analysis is not planned for the study, because it is a preventive intervention and there are no expected risks in participation other than those that occur in standard care. However, monitoring of study data will be regular and continuous throughout the trial and will be overseen by the DSMB and Steering Committee Investigators.

9.4 Outcomes

Most of the trial outcomes will have regular biochemical or survey measures, while a few categories require qualitative (e.g. interviews) and/or derived estimates (e.g. costs).

Since intermediate endpoints of Hemoglobin A1c, blood pressure, and LDL-cholesterol are being measured for the study, an Endpoint Adjudication Subcommittee is not necessary for their classification. However, an **Endpoint Adjudication Subcommittee (*Appendix 4* for description)** will be designated to classify adverse events and other hard clinical endpoints (i.e. MI, unstable angina, stroke, all cause mortality, CV mortality,

PROTOCOL

vision-threatening retinopathy, malignant hypertension, rest pain of legs, amputation, major infection) to be assessed for analysis.

Study measures are listed in **Table 2** below.

9.4.1 Primary outcome

The study has one primary outcome of interest, multiple CVD risk factor control targets: at least two targets including HbA1c < 7.0% and at least one of: BP < 130/80 mmHg or LDL-cholesterol < 100 mg/dl (LDL-cholesterol < 70 mg/dl for those with history of CVD event).

9.4.2 Secondary outcomes

Assessment questionnaires/tools are listed in bold below and are described in detail in **Table 3.**

The secondary outcomes include:

(1) Single risk factor control targets, as demonstrated by:
   c. at least an absolute 10% point greater proportion of participants in the intervention group achieving good glycemic control (HbA1c < 7%);
   d. at least an absolute 10% point greater proportion of participants in the intervention group achieving blood pressure control (systolic BP < 130 and diastolic BP < 80 mmHg); and
   e. at least an absolute10% point greater proportion of participants in the intervention group achieving lipid control (LDL-cholesterol < 100 mg/dl; < 70 mg/dl for those with history of CVD event)

(2) Quality of care measures, as demonstrated by participant and provider adherence to:
   a. currently advocated CVD risk factor management guidelines (i.e. proportion of patients prescribed and/or using lipid- or BP-lowering medication, where indicated; proportion of participants smoking who have stopped; proportion of patients who were given lifestyle modification advice and/or adhering to dietary and physical activity targets) and
   b. evidence-based processes of care (i.e. use of aspirin and/or RAS-modifiers; and annual eye, foot, dental and urine examinations);
   c. Assessment methods:
      i. *Participant Perspective (Self-management):* **Summary of Diabetes Self-Care Activities (SDSCA)**
      ii. *Provider Perspective:* Electronic Health Records from DSS (Documentation by Care Coordinator and Research Officer)

(3) Patient-related outcomes of:
   a. Participant health-related quality of life (QoL)
      i. Event-related: **European Quality of Life 5 Dimensions (EQ-5D)**
      ii. General: **Health Utility Index (HUI-3)**
   b. Treatment satisfaction
      i. Diabetes Treatment Satisfaction Questionnaire (DTSQ)

(4) To evaluate the sustainability of the intervention using the following methods:
   a. Cost-effectiveness of the intervention by assessing the incremental costs and benefits of the

intervention versus standard care for the clinic facility.

i. Questionnaire: **Frequency/Costs of Care (See *Section 9.5.2* for more detail)**

ii. Electronic health records of DSS; Clinical Administration System

b. Assessments of acceptability of the intervention from the provider perspective through interviews and the patient perspective in the final questionnaire. Patients who drop out of the study will be followed up by a Research Coordinating Centre staff to explore reasons for discontinuing involvement. **See *Section 9.5.3* for details on interview methods and qualitative data analysis.**

Table 2: Study Measures for CARRS Translational Trial

Variable	Method	Additional Test(s)	Baseline	Frequency of Repeat Measurements<br>3 monthly = intervention<br>12 monthly = all
**Demographic and Anthropometric**				
Age / Sex / Marital Status	Q		√	-
Education / Occupation / Income / Language/ Religion	Q		√	-
Height / Weight / (BMI) / Waist Circumference	M		√	3 monthly
**Risk Factor Control**				
Venous Fasting Blood Glucose (FBG)	B		√	3 monthly
Glycated Hemoglobin (HbA_1c)	B		√	3 monthly
Lipids (TC, HDL, LDL, TG)	B		√	12 monthly
Blood Pressure (BP)	M		√	3monthly
Smoking Status	Q		√	3 monthly
**Quality of Care Measures: CVD Risk Factor Guidelines and Care Processes (Participant and Physician)**				
Participant Self-Care	Q (SDSCA)		√	3 monthly
Prescriptions for glycemia, lipid, blood pressure	Q *(Participant received/ using)*	*Appropriately provided by Physician:* Cross-check with electronic health records of DSS	√	
Aspirin Use				
RAS-modifier (ACEi/ARB) Use				
Lifestyle advice				
Eye Examination (dilated pupil fundoscopy)	E	*Appropriately provided by Physician:* Cross-check with electronic health records of DSS	√	12 monthly
Foot Examination (monofilament test)	E		√	
Urine Examination (albumin:creatinine ratio)	U / B		√	
Electrocardiogram	E		√	
**Patient-related (self-reported) Outcomes**				
Quality of Life – Event related	Q (EQ-5D)		√	12 monthly
Quality of Life – General/Health Utility	Q (HUI-3)		√	

Variable	Method	Additional Test(s)	Baseline	Frequency of Repeat Measurements<br>3 monthly = intervention<br>12 monthly = all
Treatment satisfaction	Q (DTSQ)		√	
**Incidence of Complications**				
Hospitalization / ER visits / Out-patient visits	Q	Cross-check with hospital records		12 monthly
Revascularization / Surgery	Q			
Amputation / Infections*	Q			
Renal Failure / Dialysis / Transplant	Q			
**QA/Acceptability-Sustainability: Views of Intervention by Participants and Physicians**				
Self-assessment	Interview (Physician)		√	12 monthly
	Q (Patient)			Final only
**Sustainability: Cost-Effectiveness**				
Costs of care (direct& indirect)	Q	Trial data; Clinic admin. System	√	12 monthly

Q = questionnaire; E = examination; M = measurement; B = blood sample(s); U=urine sample; DTSQ = Diabetes Treatment Satisfaction Questionnaire; EQ-5D = European Quality of Life 5 Dimensions qestionnaire; HUI-3 = Health Utility Index Mark 3

**Infections to capture:* UTI, skin infections, lower respiratory tract infections/physician-diagnosed pneumonia; any serious infections requiring hospitalization and/or parenteral antibiotics;

Table 3: Description of assessment questionnaires of secondary outcomes *(Section 9.4.2)*

Questionnaire Title	Description	Use in Trial	Time Point
Participant Self-Management<br><br>***Summary of Diabetes Self-Care Activities, SDSCA*** [157]	-self-administered 11core items and 15 additional items<br>- 8 areas: general diet, specific diet, exercise, blood-glucose testing, medications, foot care, smoking, and self-care recommendations	-Assess quality of care measures from participant's perspective	Baseline and 3-monthly
Quality of Life, short<br><br>***European Quality of Life 5 Dimensions, EQ-5D*** (EuroQol Group, Rotterdam, Netherlands) [158]	-Self-administered 5 item<br>-assess acute health perceptions<br>-includes numerical scale to rate health state from 0 (worst imaginable health state) to 100 (best imaginable health state)	-Assess acute, event-related changes in well-being of participants	Baseline and 12-monthly
General Quality of Life (Health Utility)<br><br>***Health Utility Index Mark 3, HUI-3*** (Health Utilities Inc.,Dundas, Ontario Canada) [159]	-self-administered 15-item, 12-monthly<br>-8 areas: vision, hearing, speech, ambulation, dexterity, emotion, cognition, pain	-Assess general health status of participants<br>-Generate utility scores for health states, link to cost data	Baseline and 12-monthly
Treatment Satisfaction<br><br>***Diabetes Treatment Satisfaction Questionnaire, DTSQ*** [160]	-DTSQs version<br>-self-administered 8-item<br>-asses treatment satisfaction in diabetes medical therapy	-Assess participant treatment satisfaction and impact of intensive arm	Baseline and 12-monthly
Frequency/Costs of Care	-administered by research staff<br>For detail, see ***Section 9.5.2Economic Analyses***	-Assess cost-effectiveness of intervention	Baseline and 12-monthly

PROTOCOL

9.5 Data Analyses

This section on data analyses has been divided into three parts: quantitative analysis, analysis for cost-effectiveness, and qualitative analysis.

9.5.1 Quantitative Analysis

All statistical analysis of quantitative data will be performed using SAS 9.1 (SAS Institute, Cary, North Carolina) or STATA 9.0 (Statacorp, Texas). A two-sided significance level of 5% will be used for all statistical inference. All data analysis will be conducted according to the intention-to-treat principle.

Baseline differences between groups will be assessed using t-tests or Wilcoxon rank-sum tests for continuous outcomes and chi-square tests for categorical outcomes. Recruitment and retention of trial participants will be assessed by examining: number eligible to be randomized, number enrolling in the study, and dropout from regular testing at the completion of 30 months.

The effectiveness of the intervention comparing:

- baseline and close-out evaluations for between-group differences will be performed using
  - McNemar's test for categorical outcomes (proportion achieving the primary outcome of multiple risk factor targets and secondary outcomes of single risk factor targets and quality of care measures) and
  - Paired t-test for continuous outcomes (mean values of secondary outcomes including single CVD risk factors and quality of life/satisfaction scale levels).
- multiple time points for between-group and/or within-group differences will be performed using generalized estimating equation (GEE) for both categorical and continuous outcomes.

Tests for heterogeneity across sites, age groups, genders and baseline disease severity will be applied.

Any acute target organ damage, CVD events or mortality noted during the 30 months of trial follow-up (confirmed by the independent Endpoint Adjudication Committee) will be compared between study arms using Cox's longitudinal models to explore differences in incidence rates and changes over time in a preliminary manner, as the study is not powered for these end-points.

In the event that there is an interest in identifying a coordinator effect, we will use simple multi-level models which can include dummy variables for characteristics of the physician / coordinator and draw out any significant effects of seeing a particular coordinator (relative to a referent coordinator) on individual outcomes. We believe testing at an adequate number of sites, with an adequately powered sample size, sufficient number of care coordinators and using the appropriate statistical methods, will allow us to exclude the possibility that the effects shown can be attributed to one person.

9.5.2 Analysis for Cost-Effectiveness

For this trial, the economic research questions are:

1) Is the intensive, multifactorial care package [with a care coordinator (CC) and decision support system (DSS)] more cost-effective than standard care in achieving multiple risk factor control for diabetes patients in India?
2) Is the intensive, multifactorial care package [with a care coordinator (CC) and decision support system (DSS)] more cost-effective than standard care in avoiding major diabetes-related complications for diabetes patients in India?

PROTOCOL

3) Is the intensive, multifactorial care package [with a care coordinator (CC) and decision support system (DSS)] more cost-effective than standard care in avoiding mortality in productive age ranges (life years gained before age 65) for diabetes patients in India?
4) Is the intensive, multifactorial care intervention associated with improved quality of life and treatment satisfaction compared to the standard care in India?
    a. is intensive, multifactorial care package [with a care coordinator (CC) and decision support system (DSS)] more cost-effective than standard care in terms of achieving better health-related quality of life and health utility (as represented by the quality adjusted life year or QALY) in diabetes patients in India?

Cost effectiveness analysis (CEA) is a tool that aids optimization of decision-making. The CARRS trial will assess the incremental costs and benefits (effectiveness) of the intervention (intensive, multi-factorial, multi-faceted care delivery intervention to reduce cardio-metabolic risk) compared to standard care in high-risk diabetes patients with multiple, poorly-controlled risk factors. Analyses will be performed from the **perspectives of urban clinical facilities and society** (single payer national health system).

The data, such as primary endpoints for the intervention and standard care will be routinely collected according to the design of the main trial. Data on resource consumption will additionally be derived from secondary sources (clinic records, research budget), however some data will be collected from *participants through short questionnaires.* Primary and secondary data sources will be used to note unit costs of resources in the first year of the trial. Unit costs of many health resources consumed will vary based on heterogeneity among clinics (private or government-funded) as well as the locality in which they operate. Unit costs for outpatient services, outpatient procedures, laboratory tests, and consultations will be collected from each site and corroborated with research billing records. The unit cost of labor and fringe benefits, equipment, and supplies consumed by care coordinator services will be assessed similarly. The unit cost of medications will be derived from average wholesale prices of the pharmacy at each clinic in each locality. The total cost of each resource is calculated by multiplying the quantity consumed of each type of resource by the unit cost. A discount rate of 5% will be used to adjust for inflation over the study years. Sensitivity analyses will also be performed using different data sources and discount rates of 3% and 7%.

The study will collect data on the following domains:
- Direct costs (over **one year** and **over the duration of the trial**)
    - o Costs of the intervention, including:
        - research costs [software, CC salary and benefits, laboratory tests],
        - clinic costs [physician time, research officer time - labor and fringe benefits, overheads, resources for patient management (telephone calls, letters, team meetings, and adherence activities)]
        - patient costs [medication and therapeutic procedures, medical supplies, diagnostic tests other than lab and preventative tests covered by study, travel, routine clinic visits (outside the study), emergency room visits, and hospitalizations -- research staff at each clinic site will take detailed information regarding hospitalization including duration of stay, costs, procedures, diagnostics, and will seek consent from the participant to obtain a copy of the discharge summary for each hospital admission - a copy of the discharge summary will be sent to the coordinating center]
    - o Costs of standard medical care
        - Patient costs (majority of expenses are out-of-pocket) and system costs (if government-funded)
- Indirect costs (over **one year** and over **the duration of the trial**)
    - o Opportunity costs
        - Salary of participant

PROTOCOL

        - Salary of caregiver
        - Time spent at clinic waiting
        - Time spent on self-management
    - Lost productivity
        - Symptom count and severity
        - Loss of concentration, loss of function (health utility index)
- <u>Health outcomes</u>
    - Risk factors
        - Difference in risk factor parameters from baseline
        - Proportion achieving single risk factor control
        - Proportion achieving multiple risk factor control (PRIMARY OUTCOME)
    - Diabetes-related complications
        - CVD endpoints (composite of non-fatal MI or stroke, CVD death)
        - New or worsening nephropathy
        - New or worsening retinopathy
        - New or worsening foot ulcers
        - Lower limb gangrene or amputation
        - Hypoglycemia
        - Diabetic ketoacidosis
- <u>Adverse events</u>
    - hypoglycemia requiring medical attention
    - weight gain
    - any serious adverse events related to intensive intervention
- <u>Willingness-to-pay for the intervention</u>
    - Interviewing physicians in the trial [and others for sensitivity analyses] regarding their perceived value of the DSS and CC
- <u>Acceptability of intervention</u>
    - WHO Diabetes Treatment Satisfaction Questionnaire
    - Interviews

The ratio of cost to outcome from CEA compares the cost-effectiveness among the study groups. The numerator of the CE ratio for each study group will reflect net costs, incorporating research (limited to those costs that are *directly applicable to patient care* and not investigator time and planning efforts) and out-of-research costs to the clinic and patients. The denominator will represent the net "effectiveness" outcome, tallying all benefits and harms of the study group's intervention (or lack thereof). The primary endpoints of achieving multiple risk factor control will be considered the primary outcome measure for this economic evaluation. Altogether, effectiveness measures will include: 1) net achieved multiple risk factor control, 2) diabetes complication-free life year gained, 3) life-year gained, 4) quality-adjusted life-year (QALY) gained. Diabetes-related complication free-year is defined as time until first occurrence of CVD endpoints, end-stage nephropathy, sight-threatening retinopathy, or amputation. The measure of life-year gained is determined by the difference in number of life–years between intensive therapy and standard therapy. QALY's will be calculated using utility values derived from the HUI-3.

The incremental cost-effectiveness ratio (ICER) is represented by net incremental costs to net incremental effectiveness of the intervention versus standard care, within the duration of the trial. The formula is shown here:

$$\text{ICER} = \frac{(\text{Mean Cost}_{\text{intervention}} - \text{Mean Cost}_{\text{control}})}{(\text{Mean Effect}_{\text{intervention}} - \text{Mean Effect}_{\text{control}})}$$

The ICER is a point estimate; confidence intervals will be calculated using bootstrap methods. In addition, sensitivity analyses will be performed in order to examine societal effects of key parameters on CE ratios

PROTOCOL

(e.g. different wage rates or market value of intervention components). The ICER will be calculated for each clinic, given the heterogeneity among different providers, and also for all clinics combined (reflecting the trial as a whole). Depending on the outcomes of the trial, a series of ratios may be calculated for several health outcomes, including cost effectiveness of the intervention (for achieving the primary outcome of multiple risk factor control), cost per year of productive life gained, costs per event/complication avoided. In addition, data regarding events, mortality, and health-related quality of life (including health utilities) will permit calculation of the quality-adjusted life years, representing not only health decrements or improvements, but also the quality of life associated with a given health state. We will therefore use these to conduct cost-utility analysis (CUA) of the intervention group versus standard care during an extended follow-up of the trial participants. The formula for the incremental cost-utility ratio (ICUR) is below:

$$\text{ICUR} = \frac{(\text{Mean Cost}_{\text{intervention}} - \text{Mean Cost}_{\text{control}})}{(\text{Mean QALY}_{\text{intervention}} - \text{Mean QALY}_{\text{control}})}$$

9.5.3 **Qualitative Analysis**

Qualitative methods will be applied to assess acceptability and sustainability of the intervention by a subsample of study physicians. The data from the interviews will also be used primarily for quality assurance of the intervention administration (see Table 4: Quality Assurance Strategy, pg 49). A maximum of 3 physicians from each clinic site (maximum total n = 24) will be selected for 4 interviews throughout the trial - for a maximum of 96 physician interviews.

Physicians: The physicians interviewed will be the main Investigator and co-Investigators at each clinic site. They will first be interviewed by a trained RCC study staff during the site initiation visit regarding their diabetes care practices and views on the intervention. The same physicians will interviewed during the following 2 annual monitoring visits and after study closing by the RCC monitor, to understand any changes in their practice and impressions and feedback regarding the intervention. Refer to **Form K – Interview Guide_Physician (Appendix 5)** for details on the questions and themes to be captured.

The interviews of not more than 30 minutes will either be conducted in-person or by phone by a trained researcher that is not affiliated directly with any of the sites, to avoid any bias. The interviews will be audio-taped with the permission of the physician participant using written consent (Appendix 2).

Audiotapes of interviews will be transcribed, and the transcriptions audited for accuracy. The transcripts will be de-identified prior to analysis. The MAXqda (2007) program will be used to manipulate textual data for analysis. Analysis of the textual data will follow the grounded theory methodology whereby key themes are identified inductively from the textual data. These themes will then be compared using structured comparisons to identify specific issues relevant to sub-groups of participants.

PROTOCOL

10. DATA COLLECTION AND QUALITY ASSURANCE

10.1 Data Collection Forms

Patient information collected for the trial is recorded on the appropriate Case Report Forms (CRFs) and entered into the database via the corresponding electronic Case Report Forms (eCRFs) found in the electronic health records (EHR) component of the DSS, which also serves as the web-based data management system. All completed eCRFs must be submitted to the Research Coordinating Centre (RCC) at the end of every month. (Detailed instructions on how to fill the CRF's are included in *Manual of Procedures*.)

10.2 Data Management

Under the supervision of the trial project manager, a data management system will be administered by the Research Coordinating Centre (RCC), data administrator and IT technician. They will execute timely transfers, confirm receipt, organize, and back-up all study data. The data management system for the trial is in-built since the DSS is web-enhanced and encompasses the following features: secure, password-protected access; electronic data capture from remote clinic sites; automated edit tracking; audit trails; programming for quality assurance checks; utility to export to statistical software; validation tools for data-entry (split screen views); and encrypted transfer facilities.

The trial project manager will monitor study enrollment, loss to follow up, adherence and satisfaction with intervention, as well as adverse events. These matters will be regularly communicated to an independent designated Data Safety and Monitoring Board that does not represent a conflict of interest.

Whether the database server is onsite at the RCC in New Delhi or in another location (depending on the vendor), the server will have restricted-access, and regular back-up schedules and appropriate server security procedures (to ward off unauthorized data retrieval attempts) will be instituted. All study participants will be identified by unique digit-based numerics. These regulations that ensure complete patient and data safety and confidentiality will be instituted and documented meticulously.

10.3 Quality Assurance

The study will be conducted in accordance with the International ICH Guidelines for Good Clinical Practice with all relevant local, national, and international regulations.

10.3.1 **Training**

The RCC is responsible for data accuracy, consistency and quality. All Site research staff will be trained on the study protocol and procedure manuals before the start of patient recruitment. Training of care coordinators could also be facilitated from time to time if required. In the event of incomplete, incongruous or ambiguous data, the Site Investigator and/or coordinator will be contacted for clarification by the RCC and Quality Control Subcommittee that monitor study data and patient safety.

Study data derived from laboratories and participant follow-up visits, specialist clinic attendance, and preventative screening will all be collected using standardized tools and instruments, from certified laboratories with internal quality control protocols and subject to external quality verification through

PROTOCOL

regular data monitoring, checks, and interim analyses, with corrective actions executed as required, throughout the duration of the study.

10.3.2 Quality Control Committee

The membership of the Data Management/Quality Control Committee can be found in **Section 12: Study Organization.** This committee will have indirect access to the central software system (through the RCC staff) that collects all the trial data from the different Clinic Sites for monitoring and analysis. The committee will review the electronic data uploaded by the all the sites.

10.3.3 Trial Documentation / Data Quality

The Site Principal Investigator will maintain an on-site study binder (Trial Master File) that will contain the trial protocol, Procedure Manual, Patient informed consent form, Institutional Review Board document, medication records, general trial correspondence and patient screening logs, for reference.

All required study information must be recorded on source documents or the appropriate worksheet and corresponding eCRF. The Site Investigator is responsible for ensuring regular submission of the completed eCRFs to the RCC by the web-based data management system.

Case Report Forms and biological samples will be labeled with a unique study identifier. Ten percent of CRFs will be duplicated and the copy will be used for double data entry. All quantitative data will be entered into a Microsoft Access database and audited for accuracy. Coded forms will be kept separately from the code list to maintain confidentiality. Study staff at the RCC will check missing and outlier values monthly. All forms will be stored in a locked file cabinet in a locked office.

All worksheets, supporting source documents and administrative records must be retained by the Site Investigator for a minimum of three years (as per the amended Schedule Y Guidelines: Drug and Cosmetic Rules, 1945) following the last notification of approval by an appropriate regulatory authority.

The Site Investigator should refer to the associated *Manual of Procedures* for further information regarding details of the procedures to be followed during the course of the trial.

10.3.4 Monitoring

During the study, representatives of the RCC will visit all the study sites on at least 3 occasions in the first year and on at least two occasions each year thereafter. The purpose of these visits will be to ensure that the study is conducted according to the protocol and good clinical practice guidelines are being followed. These quality control reviews will also inspect study records and source documents for specific verification of participant details, data quality, and completeness of intervention implementation.

Access to Case Report Forms, source documents, and other study files must be made available at all study sites for monitoring and audit purposes at these monitoring visits during the course of the study and after the study. Any deviations will be documented by the assigned monitoring personnel.

See Table 4 below for full details.

PROTOCOL

Table 4: Quality Assurance Strategy for CARRS Translation Trial

	Quality Assurance	Quality Verification and Control	
	**Design and Planning**	**During Study**	**Analysis**
**CARRS Research Coordinating Center**	• IRB approval & peer-reviewed protocols<br>• Investigator Certification<br>• Translation of materials into local language(s)<br>• Peer-reviewed development of algorithm guidelines and decision-support software	• Manage randomization sequence and allocations – secure and limited access<br>• Review of randomization efficacy<br>• Monitor study procedures, documentation, and adherence to protocol at each clinic site; monitor acceptability, patient safety, progress, and satisfaction	• Central analyses to test study hypotheses<br>• Evaluate validity of findings prior to publication
**Care Coordinators**	Selective recruitment (stringent criteria)<br>Extensive training in:<br>• Following algorithms<br>• Software use and recording data<br>• Coordination of activities<br>• Motivational techniques	• Physician (site investigator) supervision and acceptability - **Interviews (Form K)**<br>• Regular evaluation with feedback<br>***See 9.6.3 Qualitative Data Analysis***<br>• Regular evaluation by RCC/Quality Control Subcommittee	• Vigilance in analysis for possible clustering effect(s)
**Laboratories**	• Laboratory selection (all certified) – one per clinic site where ALL study-related tests are done<br>• Identify central reference laboratory (AIIMS)<br>• Centrally-developed calibration guidelines and checks for equipment<br>• Develop internal and external quality assessment protocols and schedule<br>• Specific protocols for biochemical assays<br>• Trained staff to handle, process & store samples	• Lyophilized samples from reference laboratory sent to all labs<br>• Assessment of intra- and inter-laboratory variability<br>• Provision to re-analyze 5-10% samples in reference laboratory	• Assess intra- and inter-laboratory coefficients of variation
**Data Storage and Management**	• Software development and piloting (involving both dummy data only AND patient data testing)<br>• Controlled and differential access to software system at clinic sites and RCC – testing pre-trial<br>• Establish security procedures<br>• Establish back-up server(s)<br>• Establish data transfer, data cleaning, editing, and verification systems	• Locked, password-protected storage with active back-up<br>• Review of audit trails for data modification/irregularities by RCC/QC Subcommittee<br>• Independent observers (DSMB)to monitor enrollment, retention, and adverse effects	• Small percentage of CRFs to undergo duplicate data entries to assess accuracy<br>• Validity checks<br>• Reports of patient safety, progress, and audits to be provided

11. PARTICIPANT RIGHTS AND CONFIDENTIALITY

11.1 Institutional Review Board (IRB) Review

This protocol and the patient information sheet-informed consent document and any subsequent modifications will be reviewed and approved by the ethics committees of all the Clinic Sites; the IEC of the Public Health Foundation of India (PHFI), who is responsible for oversight of the study; and Emory University IRB as a partnering research institute. The protocol will also undergo review by India's Health Ministry's Screening Committee (HMSC).

11.2 Informed Consent Form

A signed consent form will be obtained from each participant. For participants who are illiterate, a (legally acceptable representative (LAR) or 3rd party witness must also sign the consent form. The consent form describes the purpose of the study, the procedures to be followed, and the risks and benefits of participation. A copy will be given to each participant or legal guardian and this fact will be documented in the participant's record. A copy of the consent form can be found in **Appendix 1**.

11.3 Participant Confidentiality

The database server and additional off-site servers will both be housed in restricted-access buildings. Paper CRFs will be stored in locked cabinets in similarly secured buildings at participating clinics and will be accessed only by permitted study staff and the monitor from the RCC. Names and other easily recognizable identifiers will be removed from all CRFs prior to data entry and analysis. Instead, participant identification numbers (PID) will be used so that data and specimens may be linked; these are not meaningful to casual observers without access to the original study logs (accessible only to permitted research team). Any data, specimens, forms, reports, video recordings, and other records that leave the Clinic Site will be identified only by the PID to maintain confidentiality. All data files will be maintained under password protection at all times. All paper records will be kept in a locked file cabinet. The study records will be available to regulatory agencies, including IRBs, the DSMB, ICMR, and OHRP. The funders, NHLBI and Ovations, also have a right to access the study records.

All study staff will be trained in procedures to minimize the potential for breaches of confidentiality, including but not limited to, ensuring that all files are closed and no conversations about individual study participants occur in public settings.

11.4 Study Discontinuation

The study may be discontinued at any time by the site IRBs (for their specific clinical site only), PHFI IEC, ICMR, NHLBI, the OHRP or other applicable agencies as part of their duty to ensure that participants are protected.

PROTOCOL

12. <u>STUDY ORGANIZATION</u>

12.1 Overview

The CARRS Translation Trial is a study of the Center for Cardiometabolic Risk Reduction in South Asia (CARRS), under the Public Health Foundation of India (PHFI), contracted by the National Heart, Lung and Blood Institute (NHLBI), USA. CARRS is one of the eleven Centers of Excellence funded by NHLBI and UnitedHealth Chronic Disease Initiative to enable research and training in developing countries on chronic cardiovascular and lung diseases. Partner institutes of COE-CARRS include All India Institute of Medical Sciences (New Delhi), Madras Diabetes Research Foundation (Chennai), and Aga Khan University (Karachi, Pakistan). The developed country partner is Emory University (Atlanta, USA).

The trial has a designated Research Coordinating Center (RCC) to oversee the successful design and conduct of the trial at the eight selected Clinic Sites (CS) in South Asia (see Figure 5). The Steering Committee supports the Research Coordinating Center and provides the scientific leadership for the trial (see Figure 6). The Executive Committee serves as the operational arm, for day-to-day management and making any recommendations to the Steering Committee.

12.2 Clinical Sites

The 1120 trial participants will be recruited, randomized, treated and followed at the 8 Clinical Sites. Each Clinical Site consists of an out-patient diabetes clinic which may be an individual practice or under a hospital facility. The RCC will work with the Clinical Sites on issues of trial setup and training, recruitment, compliance with protocol, data management, and quality control. The Clinical Sites will transmit their data directly to the RCC.

12.3 The Research Coordinating Center

The Research Coordinating Center (RCC), with input from the Steering Committee, is responsible for developing the protocol, certification of Clinical Sites; developing and distributing the Manuel of Procedures; training trial personnel in the standardized protocol implementation and data collection; collecting and managing all trial data; quality control; analyzing data; and preparing reports for the Data Safety Monitoring Board, Steering Committee and NHLBI/Westat (NHLBI's administrative coordinating center). The RCC will conduct at least annual visits to each Clinical Site to monitor and assure high performance during the trial.

During recruitment for the trial, the RCC will be responsible for monitoring patient recruitment and will provide monthly progress reports to the Steering Committee and other required entities.

12.4 NHLBI/Westat

CARRS Translation Trial is sponsored by the National Heart, Lung and Blood Institute (NHLBI). Westat is a group that serves as NHLBI's administrative coordinating center. NHLBI/Westat is involved in administration of CARRS and other Centers of Excellence in Cardiometabolic Research. Representatives from NHLBI/Westat participate in the fiscal management and general monitoring of the trial. Support for trial development and management, analysis, and publication is provided by NHLBI/Westat.

PROTOCOL

12.5 Laboratories

Central Laboratory: The laboratory at the All India Institute of Medical Sciences will serve as the Central Laboratory. The Central Laboratory will coordinate the external quality assurance methods for all clinical sites.

Clinical Site Laboratories**:** Each clinical site will identify one laboratory that has NABL certification to serve as the trial laboratory for assessment of participant investigations. The Clinical Site laboratories will undergo external quality control checks arranged by the RCC for cross-site standardization.

.

12.6 The CARRS Trial Steering Committee, Committee of Investigators, Subcommittees, the Executive Committee, and the Advisory Committee

The CARRS Trial Steering Committee provides the overall leadership for the study and establishes the scientific and administrative policy. It is composed of Principal Investigators from the Center for Cardiometabolic Risk Reduction in South Asia (CARRS) at PHFI [D Prabhakaran, K Srinath Reddy]; partner institutes including All India Institute of Medical Sciences (AIIMS)-New Delhi [N Tandon], Madras Diabetes Research Foundation (MDRF)-Chennai [V Mohan], Aga Khan University (AKU)-Karachi [M Kadir], and developed partner: Emory University-Atlanta, USA [KM Venkat Narayan, M Ali]; as well as the senior research coordinator and the trial project manager from the RCC. This Steering Committee oversees the overall conduct of the trial. The Steering Committee and the Committee of Investigators developed the trial design, prepared the final protocol, and approved the study forms and manual of operations. During the study collection phases of the trial, this committee oversees the data collection practices and procedures to identify and correct deficiencies. The committee will also consider and adopt changes in the study protocol or procedures as necessary during the trial.

The Committee of Investigators is comprised of the Principal Investigators from the CARRS network mentioned above, the Clinical Site Investigators and senior research staff.

There are 5 standing subcommittees. These are the Data Management and Quality Control Subcommittee; the Publication, Presentation and Ancillary Studies (PP&A) Subcommittee; the Endpoint Adjudication Subcommittee; the Qualitative Methods Subcommittee; and the Cost-Effectiveness Subcommittee. The responsibilities of these subcommittees are provided in ***Appendix 4***.

An Executive Committee serves as the operational arm of the Steering Committee and makes decision on behalf of the steering committee on day-to-day operational issues requiring immediate action and makes recommendations to the Steering Committee, where necessary. It will meet weekly by conference call to review trial progress and any study issues that may arise. This committee will also develop time lines for the accomplishment of tasks and Steering Committee Meeting agendas. The members of the Executive Committee include the Steering Committee Chair, the Steering Committee Vice-chair, and the RCC personnel.

The principal investigators also have the assistance of an Advisory Committee of distinguished scientists and dignitaries. The Advisory Committee will be composed of: M.K. Bhan, (*Chair*) Secretary to the Government of India, Department of Biotechnology, Ministry of Science and Technology; Shah Ebrahim, Professor, London School of Hygiene and Tropical Medicine; Jeffrey Koplan, Vice President, Global Health and Director, Global Health Institute, Emory University; formerly, Director, CI.C (1998-2002); K. Anji Reddy, Chairman, Dr. Reddy's Laboratories, India; Vijayalakshmi Ravindranath, Director, National Brain Research

PROTOCOL

Center, Manesar; and Allan Sniderman, Director, Mike Rosenbloom Laboratory for Cardiovascular Research, Edwards Professor of Cardiology, McGill University, Montreal, Canada. See **Figure 5** for the organization of the committees and subcommittees.

12.7 Data Safety Monitoring Board (DSMB)

An independent Data Safety Monitoring Board will monitor data and oversee patient safety. Members of the Board, appointed by the Steering Committee, are senior experts in the areas of cardiovascular medicine, diabetes, biostatistics, epidemiology, and bioethics. The Steering Committee Chair, Vice-chair and senior staff of the RCC will participate in DSMB meetings as non-voting members. The DSMB will meet at least once a year to monitor safety, to advise NHLBI about the study progress, including contractor performance, and to make recommendations to the Steering Committee regarding study continuation and protocol changes. Additionally, the RCC will provide the DSMB Chair any serious adverse event reports and trial data at his/her request at regular intervals to ensure identification of any other major adverse outcomes.

12.8 Conflict of Interest Policy

The Center for Cardiometabolic Risk Reduction in South Asia (CARRS) will establish a policy regarding Conflict of Interest to be adhered to by all investigators. The policy will provide rules to conduct the trial in an unbiased and informed manner that meets public standards.

Figure 5: Study Organization of CARRS Trial

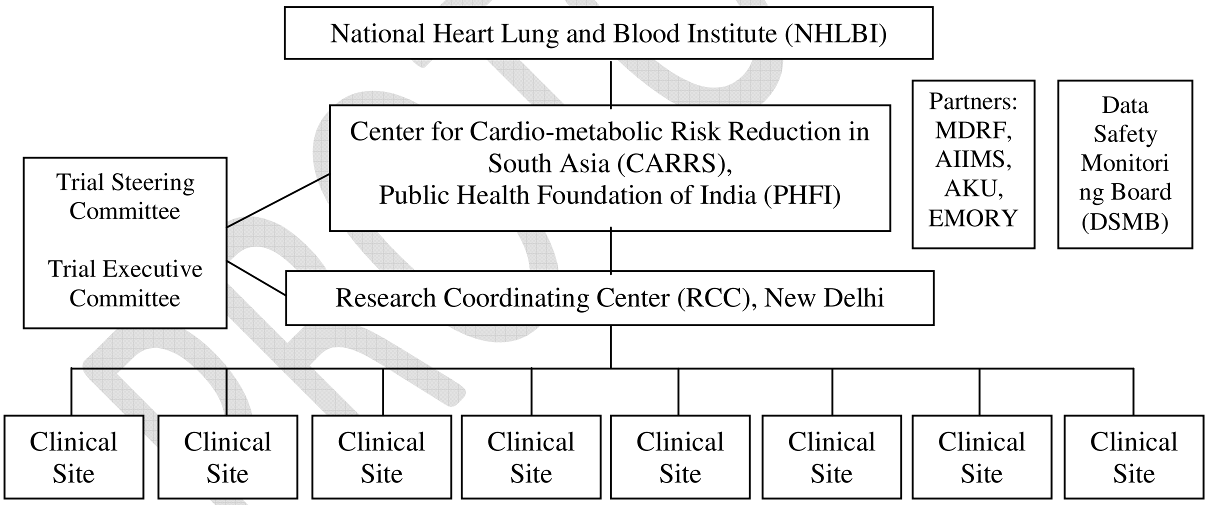

Figure 6: COE-CARRS Structure and Trial Committees

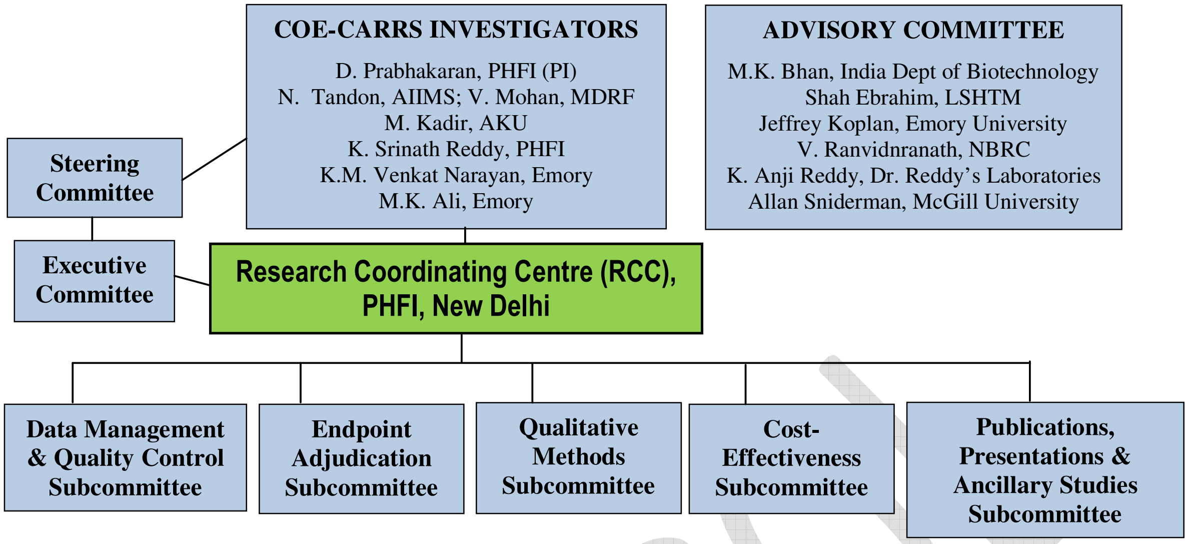

13. PUBLICATION OF RESEARCH FINDINGS

At defined points over the course of the five years, formal analysis of quantitative and qualitative data will be reviewed for validity and subsequent publication. The investigators will form a Publication, Presentation and Ancillary Studies (PP&A) subcommittee, which will develop a suitable policy protecting the CARRS network's rights regarding ownership of study materials and data. Generally, publication of manuscripts from the study will be in the name of the research group, with each individual study investigator named personally at the end of the report; the support of NHLBI will be acknowledged. As required, NHLBI/Westat (NHLBI's administrative coordinating center) will be provided a minimum 45-day advance notice of intent to submit a manuscript for publication and a copy of the manuscript. However, full editorial control will reside with the Steering Committee (SC). The Research Coordinating Center will prepare regular study reports to be submitted to regulatory authorities (e.g. NHLBI/Westat).

14. FUNDING

The translation trial is funded by an award from the National Heart Lung and Blood Institute (NHLBI), part of the National Institutes of Health in Bethesda, Maryland, USA. The trial has supplemental funding from Ovations (Ovations) Chronic Disease Initiative, UnitedHealth Group, USA. NHLBI and Ovations have funded the development of the research group, **Center of Excellence-Center for Cardiometabolic Risk Reduction in South Asia (COE-CARRS)**, which is conducting the trial. The study was initiated and designed by the CARRS investigators and the data will be collected, analyzed and published independent of NHLBI and Ovations.

15. ADMINISTRATIVE CLAUSES

The administrative clauses relating to this protocol are covered by the Clinical Trial Agreement (CTA) between the Clinic Site and the Research Coordinating Center, and the CARRS network base at PHFI and NHLBI.

PROTOCOL

16. REFERENCES

1. Global Health Centers of Excellence, NHLBI and UnitedHealth. 2009. (Accessed at http://www.nhlbi.nih.gov/about/globalhealth/centers/index.htm.)
2. Mathers CD, Lopez AD, Murray CJL. The Burden of Disease and Mortality by Condition: Data, Methods, and Results for 2001. In: Lopez AD, Mathers CD, Ezzati M, Jamison DT, Murray CJL, eds. Global Burden of Disease and Risk Factors,. New York: Oxford University Press; 2006.
3. The human, social & economic impact of diabetes. 2007. (Accessed February 2008, at http://www.idf.org/home/index.cfm?node=41.)
4. Economic Intelligence Unit. The silent epidemic: An economic study of diabetes in developed and developing countries. New York, London, Hong Kong: The Economist June 2007.
5. Kempler P. Learning from large cardiovascular clinical trials: classical cardiovascular risk factors. Diabetes research and clinical practice 2005;68 Suppl1:S43-7.
6. Stamler J, Vaccaro O, Neaton JD, Wentworth D. Diabetes, other risk factors, and 12-yr cardiovascular mortality for men screened in the Multiple Risk Factor Intervention Trial. Diabetes Care 1993;16:434-44.
7. Ramachandran A, Snehalatha C, Latha E, Satyavani K, Vijay V. Clustering of cardiovascular risk factors in urban Asian Indians. Diabetes care 1998;21:967-71.
8. Pradeepa R, Mohan V. The changing scenario of the diabetes epidemic: implications for India. Indian J Med Res 2002;116:121-32.
9. Hughes LO, Raval U, Raftery EB. First myocardial infarctions in Asian and white men. BMJ 1989;298:1345-50.
10. McKeigue PM, Ferrie JE, Pierpoint T, Marmot MG. Association of early-onset coronary heart disease in South Asian men with glucose intolerance and hyperinsulinemia. Circulation 1993;87:152-61.
11. Ghaffar A, Reddy KS, Singhi M. Burden of non-communicable diseases in South Asia. BMJ 2004;328:807-10.
12. Mohan V, Sandeep S, Deepa R, Shah B, Varghese C. Epidemiology of type 2 diabetes: Indian scenario. Indian J Med Res 2007;125:217-30.
13. Enas EA, Mehta J. Malignant coronary artery disease in young Asian Indians: thoughts on pathogenesis, prevention, and therapy. Coronary Artery Disease in Asian Indians (CADI) Study. Clin Cardiol 1995;18:131-5.
14. Radha V, Mohan V. Genetic predisposition to type 2 diabetes among Asian Indians. Indian J Med Res 2007;125:259-74.
15. Chambers JC, Elliott P, Zabaneh D, et al. Common genetic variation near MC4R is associated with waist circumference and insulin resistance. Nat Genet 2008;40:716-8.
16. Yajnik CS. Early life origins of insulin resistance and type 2 diabetes in India and other Asian countries. J Nutr 2004;134:205-10.
17. Ramachandran A. Epidemiology of diabetes in India--three decades of research. J Assoc Physicians India 2005;53:34-8.
18. Lev-Ran A. Human obesity: an evolutionary approach to understanding our bulging waistline. Diabetes Metab Res Rev 2001;17:347-62.
19. Joshi P, Islam S, Pais P, et al. Risk factors for early myocardial infarction in South Asians compared with individuals in other countries. Jama 2007;297:286-94.
20. Diaz VA, Mainous AG, 3rd, Baker R, Carnemolla M, Majeed A. How does ethnicity affect the association between obesity and diabetes? Diabet Med 2007;24:1199-204.
21. McKeigue PM, Marmot MG, Syndercombe Court YD, Cottier DE, Rahman S, Riemersma RA. Diabetes, hyperinsulinaemia, and coronary risk factors in Bangladeshis in east London. Br Heart J 1988;60:390-6.
22. McKeigue PM, Shah B, Marmot MG. Relation of central obesity and insulin resistance with high diabetes prevalence and cardiovascular risk in South Asians. Lancet 1991;337:382-6.
23. Vardan S, Mookherjee S, Sinha AK. Special features of coronary heart disease in people of the Indian sub-continent. Indian Heart J 1995;47:399-407.
24. Diabetes Atlas 3rd Edition. 2007. (Accessed February 2008, at www.eatlas.idf.org/media.)
25. Gupta R, Kumar P. Global Diabetes Landscape - Type 2 Diabetes Mellitus in South Asia: Epidemiology, Risk Factors, and Control. Insulin 2008;3:78-94.
26. Gupta R, Joshi P, Mohan V, Reddy KS, Yusuf S. Epidemiology and causation of coronary heart disease and stroke in India. Heart 2008;94:16-26.

27. Wild S, Roglic G, Green A, Sicree R, King H. Global prevalence of diabetes: estimates for the year 2000 and projections for 2030. Diabetes Care 2004;27:1047-53.
28. International Diabetes Federation. Diabetes Atlas 3rd Edition. Brussels; 2006.
29. Gaziano TA, Reddy KS, Paccaud F, Horton S, Chaturvedi V. Cardiovascular Disease. In: Jamison DT, Breman JG, Measham AR, et al., eds. Disease Control Priorities in Developing Countries, 2nd ed. New York: Oxford University Press; 2006:645-62.
30. Lopez AD, Murray CC. The global burden of disease, 1990-2020. Nat Med 1998;4:1241-3.
31. Manley SE, Stratton IM, Cull CA, et al. Effects of three months' diet after diagnosis of Type 2 diabetes on plasma lipids and lipoproteins (UKPDS 45). UK Prospective Diabetes Study Group. Diabet Med 2000;17:518-23.
32. Kris-Etherton P, Eckel RH, Howard BV, St. Jeor S, Bazzarre TL. Lyon Diet Heart Study : Benefits of a Mediterranean-Style, National Cholesterol Education Program/American Heart Association Step I Dietary Pattern on Cardiovascular Disease. Circulation 2001;103:1823-5.
33. Fletcher GF, Balady GJ, Amsterdam EA, et al. Exercise standards for testing and training: a statement for healthcare professionals from the American Heart Association. Circulation 2001;104:1694-740.
34. Hill JM, Zalos G, Halcox JP, et al. Circulating endothelial progenitor cells, vascular function, and cardiovascular risk. N Engl J Med 2003;348:593-600.
35. Werner N, Kosiol S, Schiegl T, et al. Circulating endothelial progenitor cells and cardiovascular outcomes. N Engl J Med 2005;353:999-1007.
36. Laufs U, Werner N, Link A, et al. Physical training increases endothelial progenitor cells, inhibits neointima formation, and enhances angiogenesis. Circulation 2004;109:220-6.
37. Antithrombotic Trialists Collaboration. Collaborative meta-analysis of randomised trials of antiplatelet therapy for prevention of death, myocardial infarction, and stroke in high risk patients. BMJ 2002;324:71-86.
38. Hansson L, Zanchetti A, Carruthers SG, et al. Effects of intensive blood-pressure lowering and low-dose aspirin in patients with hypertension: principal results of the Hypertension Optimal Treatment (HOT) randomised trial. HOT Study Group. Lancet 1998;351:1755-62.
39. Lindholm LH, Ibsen H, Dahlof B, et al. Cardiovascular morbidity and mortality in patients with diabetes in the Losartan Intervention For Endpoint reduction in hypertension study (LIFE): a randomised trial against atenolol. Lancet 2002;359:1004-10.
40. Niskanen L, Hedner T, Hansson L, Lanke J, Niklason A. Reduced cardiovascular morbidity and mortality in hypertensive diabetic patients on first-line therapy with an ACE inhibitor compared with a diuretic/beta-blocker-based treatment regimen: a subanalysis of the Captopril Prevention Project. Diabetes Care 2001;24:2091-6.
41. Yusuf S, Sleight P, Pogue J, Bosch J, Davies R, Dagenais G. Effects of an angiotensin-converting-enzyme inhibitor, ramipril, on cardiovascular events in high-risk patients. The Heart Outcomes Prevention Evaluation Study Investigators. N Engl J Med 2000;342:145-53.
42. Brenner BM, Cooper ME, de Zeeuw D, et al. Effects of losartan on renal and cardiovascular outcomes in patients with type 2 diabetes and nephropathy. N Engl J Med 2001;345:861-9.
43. Collins R, Armitage J, Parish S, Sleigh P, Peto R. MRC/BHF Heart Protection Study of cholesterol-lowering with simvastatin in 5963 people with diabetes: a randomised placebo-controlled trial. Lancet 2003;361:2005-16.
44. Colhoun HM, Betteridge DJ, Durrington PN, et al. Primary prevention of cardiovascular disease with atorvastatin in type 2 diabetes in the Collaborative Atorvastatin Diabetes Study (CARDS): multicentre randomised placebo-controlled trial. Lancet 2004;364:685-96.
45. Baigent C, Keech A, Kearney PM, et al. Efficacy and safety of cholesterol-lowering treatment: prospective meta-analysis of data from 90,056 participants in 14 randomised trials of statins. Lancet 2005;366:1267-78.
46. Yusuf S. Two decades of progress in preventing vascular disease. Lancet 2002;360:2-3.
47. Tight blood pressure control and risk of macrovascular and microvascular complications in type 2 diabetes: UKPDS 38. UK Prospective Diabetes Study Group. Bmj 1998;317:703-13.
48. Patel A, MacMahon S, Chalmers J, et al. Effects of a fixed combination of perindopril and indapamide on macrovascular and microvascular outcomes in patients with type 2 diabetes mellitus (the ADVANCE trial): a randomised controlled trial. Lancet 2007;370:829-40.
49. Tuomilehto J, Rastenyte D, Birkenhager WH, et al. Effects of calcium-channel blockade in older patients with diabetes and systolic hypertension. Systolic Hypertension in Europe Trial Investigators. N Engl J Med 1999;340:677-84.
50. Cockcroft JR. ACE inhibition in hypertension: focus on perindopril. Am J Cardiovasc Drugs 2007;7:303-17.

PROTOCOL

51. Whelton PK, Barzilay J, Cushman WC, et al. Clinical Outcomes in Antihypertensive Treatment of Type 2 Diabetes, Impaired Fasting Glucose Concentration, and Normoglycemia: Antihypertensive and Lipid-Lowering Treatment to Prevent Heart Attack Trial (ALLHAT). Arch Intern Med 2005;165:1401-9.
52. Collins R, Peto R, MacMahon S, et al. Blood pressure, stroke, and coronary heart disease. Part 2, Short-term reductions in blood pressure: overview of randomised drug trials in their epidemiological context. Lancet 1990;335:827-38.
53. Turnbull F, Neal B, Algert C, et al. Effects of different blood pressure-lowering regimens on major cardiovascular events in individuals with and without diabetes mellitus: results of prospectively designed overviews of randomized trials. Arch Intern Med 2005;165:1410-9.
54. Estacio RO, Coll JR, Tran ZV, Schrier RW. Effect of intensive blood pressure control with valsartan on urinary albumin excretion in normotensive patients with type 2 diabetes. Am J Hypertens 2006;19:1241-8.
55. Adler AI, Stratton IM, Neil HA, et al. Association of systolic blood pressure with macrovascular and microvascular complications of type 2 diabetes (UKPDS 36): prospective observational study. Bmj 2000;321:412-9.
56. Implications of the United Kingdom Prospective Diabetes Study. Diabetes Care 2002;25:S28-32.
57. Vijayaraghavan K, Deedwania PC. The renin angiotensin system as a therapeutic target to prevent diabetes and its complications. Cardiol Clin 2005;23:165-83.
58. Pahor M, Psaty BM, Alderman MH, Applegate WB, Williamson JD, Furberg CD. Therapeutic benefits of ACE inhibitors and other antihypertensive drugs in patients with type 2 diabetes. Diabetes Care 2000;23:888-92.
59. Effects of ramipril on cardiovascular and microvascular outcomes in people with diabetes mellitus: results of the HOPE study and MICRO-HOPE substudy. Heart Outcomes Prevention Evaluation Study Investigators. Lancet 2000;355:253-9.
60. Chalmers J, Joshi R, Patel A. Advances in reducing the burden of vascular disease in type 2 diabetes. Clin Exp Pharmacol Physiol 2008;35:434-7.
61. Antithrombotic Trialists' Collaboration. Aspirin in the primary and secondary prevention of vascular disease: collaborative meta-analysis of individual participant data from randomised trials. The Lancet 2009;373:1849-60.
62. Final report on the aspirin component of the ongoing Physicians' Health Study. Steering Committee of the Physicians' Health Study Research Group. N Engl J Med 1989;321:129-35.
63. A randomised, blinded, trial of clopidogrel versus aspirin in patients at risk of ischaemic events (CAPRIE). CAPRIE Steering Committee. Lancet 1996;348:1329-39.
64. Bhatt DL, Marso SP, Hirsch AT, Ringleb PA, Hacke W, Topol EJ. Amplified benefit of clopidogrel versus aspirin in patients with diabetes mellitus. Am J Cardiol 2002;90:625-8.
65. Mohiuddin SM, Mooss AN, Hunter CB, Grollmes TL, Cloutier DA, Hilleman DE. Intensive Smoking Cessation Intervention Reduces Mortality in High-Risk Smokers With Cardiovascular Disease. Chest 2007;131:446-52.
66. Critchley JA, Capewell S. Mortality Risk Reduction Associated With Smoking Cessation in Patients With Coronary Heart Disease: A Systematic Review. JAMA 2003;290:86-97.
67. The effect of intensive treatment of diabetes on the development and progression of long-term complications in insulin-dependent diabetes mellitus. The Diabetes Control and Complications Trial Research Group. N Engl J Med 1993;329:977-86.
68. Intensive blood-glucose control with sulphonylureas or insulin compared with conventional treatment and risk of complications in patients with type 2 diabetes (UKPDS 33). UK Prospective Diabetes Study (UKPDS) Group. Lancet 1998;352:837-53.
69. Skyler JS. Effects of glycaemic control on diabetes complications and on the prevention of diabetes. Clinical Diabetes 2004;22:162-6.
70. Gerstein HC, Miller ME, Byington RP, et al. Effects of intensive glucose lowering in type 2 diabetes. N Engl J Med 2008;358:2545-59.
71. Patel A, MacMahon S, Chalmers J, et al. Intensive blood glucose control and vascular outcomes in patients with type 2 diabetes. N Engl J Med 2008;358:2560-72.
72. Beals JK. VA Diabetes Trial shows that intensive glucose control has little effect on cardiovascular risk. In: the heartorg: Lipid & Metabolic; 2008.
73. Deedwania PC, Fonseca VA. Diabetes, prediabetes, and cardiovascular risk: shifting the paradigm. Am J Med 2005;118:939-47.
74. Nathan DM, Cleary PA, Backlund JY, et al. Intensive diabetes treatment and cardiovascular disease in patients with type 1 diabetes. N Engl J Med 2005;353:2643-53.

PROTOCOL

75. Haffner SJ, Cassells H. Hyperglycemia as a cardiovascular risk factor. Am J Med 2003;115 Suppl 8A:6S-11S.
76. Ford ES, Ajani UA, Croft JB, et al. Explaining the Decrease in U.S. Deaths from Coronary Disease, 1980-2000. N Engl J Med 2007;356:2388-98.
77. Wagenknecht LE, Zaccaro D, Espeland MA, Karter AJ, O'Leary DH, Haffner SM. Diabetes and progression of carotid atherosclerosis: the insulin resistance atherosclerosis study. Arterioscler Thromb Vasc Biol 2003;23:1035-41.
78. Levitan EB, Song Y, Ford ES, Liu S. Is nondiabetic hyperglycemia a risk factor for cardiovascular disease? A meta-analysis of prospective studies. Arch Intern Med 2004;164:2147-55.
79. Holman RR, Paul SK, Bethel MA, Matthews DR, Neil HA. 10-year follow-up of intensive glucose control in type 2 diabetes. N Engl J Med 2008;359:1577-89.
80. Reichard P, Nilsson BY, Rosenqvist U. The effect of long-term intensified insulin treatment on the development of microvascular complications of diabetes mellitus. The New England journal of medicine 1993;329:304-9.
81. Srikanth S, Deedwania P. Comprehensive risk reduction of cardiovascular risk factors in the diabetic patient: an integrated approach. Cardiol Clin 2005;23:193-210.
82. Barnett AH. The importance of treating cardiometabolic risk factors in patients with type 2 diabetes. Diab Vasc Dis Res 2008;5:9-14.
83. Gaede P, Vedel P, Larsen N, Jensen GV, Parving HH, Pedersen O. Multifactorial intervention and cardiovascular disease in patients with type 2 diabetes. N Engl J Med 2003;348:383-93.
84. Gaede P, Lund-Andersen H, Parving HH, Pedersen O. Effect of a multifactorial intervention on mortality in type 2 diabetes. N Engl J Med 2008;358:580-91.
85. Wald NJ, Law MR. A strategy to reduce cardiovascular disease by more than 80%. Bmj 2003;326:1419.
86. Association AD. Standards of Medical Care in Diabetes 2009. Diabetes Care 2009;32:S13-S61.
87. Saaddine JB, Engelgau MM, Beckles GL, Gregg EW, Thompson TJ, Narayan KM. A diabetes report card for the United States: quality of care in the 1990s. Annals of internal medicine 2002;136:565-74.
88. Beckles GL, Engelgau MM, Narayan KM, Herman WH, Aubert RE, Williamson DF. Population-based assessment of the level of care among adults with diabetes in the U.S. Diabetes care 1998;21:1432-8.
89. Saydah SH, Fradkin J, Cowie CC. Poor control of risk factors for vascular disease among adults with previously diagnosed diabetes. Jama 2004;291:335-42.
90. Savage PJ. Treatment of diabetes mellitus to reduce its chronic cardiovascular complications. Current opinion in cardiology 1998;13:131-8.
91. Lifestyle and risk factor management and use of drug therapies in coronary patients from 15 countries; principal results from EUROASPIRE II Euro Heart Survey Programme. European heart journal 2001;22:554-72.
92. Nagpal J, Bhartia A. Quality of diabetes care in the middle- and high-income group populace: the Delhi Diabetes Community (DEDICOM) survey. Diabetes Care 2006;29:2341-8.
93. Raheja BS, Kapur A, Bhoraskar A, et al. DiabCare Asia--India Study: diabetes care in India--current status. The Journal of the Association of Physicians of India 2001;49:717-22.
94. Mendis S, Abegunde D, Yusuf S, et al. WHO study on Prevention of REcurrences of Myocardial Infarction and StrokE (WHO-PREMISE). Bulletin of the World Health Organization 2005;83:820-9.
95. Xavier D, Pais P, Devereaux PJ, et al. Treatment and outcomes of acute coronary syndromes in India (CREATE): a prospective analysis of registry data. Lancet 2008;371:1435-42.
96. Brown JB, Nichols GA, Perry A. The burden of treatment failure in type 2 diabetes. Diabetes Care 2004;27:1535-40.
97. Narayan KM, Benjamin E, Gregg EW, Norris SL, Engelgau MM. Diabetes translation research: where are we and where do we want to be? Annals of internal medicine 2004;140:958-63.
98. Selby JV, Swain BE, Gerzoff RB, et al. Understanding the gap between good processes of diabetes care and poor intermediate outcomes: Translating Research into Action for Diabetes (TRIAD). Med Care 2007;45:1144-53.
99. Sikka R, Waters J, Moore W, Sutton DR, Herman WH, Aubert RE. Renal assessment practices and the effect of nurse case management of health maintenance organization patients with diabetes. Diabetes Care 1999;22:1-6.
100. O'Hare JP, Raymond NT, Mughal S, et al. Evaluation of delivery of enhanced diabetes care to patients of South Asian ethnicity: the United Kingdom Asian Diabetes Study (UKADS). Diabet Med 2004;21:1357-65.

PROTOCOL

101. Choe HM, Mitrovich S, Dubay D, Hayward RA, Krein SL, Vijan S. Proactive case management of high-risk patients with type 2 diabetes mellitus by a clinical pharmacist: a randomized controlled trial. Am J Manag Care 2005;11:253-60.
102. Mullen BA, Kelley PA. Diabetes nurse case management: an effective tool. J Am Acad Nurse Pract 2006;18:22-30.
103. Philis-Tsimikas A, Walker C, Rivard L, et al. Improvement in diabetes care of underinsured patients enrolled in project dulce: a community-based, culturally appropriate, nurse case management and peer education diabetes care model. Diabetes Care 2004;27:110-5.
104. Taylor CB, Miller NH, Reilly KR, et al. Evaluation of a nurse-care management system to improve outcomes in patients with complicated diabetes. Diabetes Care 2003;26:1058-63.
105. Couch C, Sheffield P, Gerthoffer T, Ries A, Hollander P. Clinical outcomes in patients with type 2 diabetes managed by a diabetes resource nurse in a primary care practice. Proc (Bayl Univ Med Cent) 2003;16:336-40.
106. Krein SL, Klamerus ML, Vijan S, et al. Case management for patients with poorly controlled diabetes: a randomized trial. Am J Med 2004;116:732-9.
107. Litaker D, Mion L, Planavsky L, Kippes C, Mehta N, Frolkis J. Physician - nurse practitioner teams in chronic disease management: the impact on costs, clinical effectiveness, and patients' perception of care. J Interprof Care 2003;17:223-37.
108. Fera T, Bluml BM, Ellis WM, Schaller CW, Garrett DG. The Diabetes Ten City Challenge: interim clinical and humanistic outcomes of a multisite community pharmacy diabetes care program. J Am Pharm Assoc (2003) 2008;48:181-90.
109. Martin I.C, Berger ML, Anstatt DT, et al. A randomized controlled open trial of population-based disease and case management in a Medicare Plus Choice health maintenance organization. Prev Chronic Dis 2004;1:A05.
110. Gabbay RA, Lendel I, Saleem TM, et al. Nurse case management improves blood pressure, emotional distress and diabetes complication screening. Diabetes Res Clin Pract 2006;71:28-35.
111. Closing the gap: effect of diabetes case management on glycemic control among low-income ethnic minority populations: the California Medi-Cal type 2 diabetes study. Diabetes Care 2004;27:95-103.
112. Aubert RE, Herman WH, Waters J, et al. Nurse case management to improve glycemic control in diabetic patients in a health maintenance organization. A randomized, controlled trial. Ann Intern Med 1998;129:605-12.
113. Davidson MB, Ansari A, Karlan VJ. Effect of a nurse-directed diabetes disease management program on urgent care/emergency room visits and hospitalizations in a minority population. Diabetes Care 2007;30:224-7.
114. Fanning EL, Selwyn BJ, Larme AC, DeFronzo RA. Improving efficacy of diabetes management using treatment algorithms in a mainly Hispanic population. Diabetes Care 2004;27:1638-46.
115. Gary TL, Bone LR, Hill MN, et al. Randomized controlled trial of the effects of nurse case manager and community health worker interventions on risk factors for diabetes-related complications in urban African Americans. Prev Med 2003;37:23-32.
116. Lin D, Hale S, Kirby E. Improving diabetes management: structured clinic program for Canadian primary care. Can Fam Physician 2007;53:73-7.
117. Bodenheimer T, MacGregor K, Stothart N. Nurses as leaders in chronic care. BMJ 2005;330:612-3.
118. O'Connor PJ, Desai J, Solberg LI, et al. Randomized trial of quality improvement intervention to improve diabetes care in primary care settings. Diabetes Care 2005;28:1890-7.
119. Hetlevik I, Holmen J, Kruger O, Kristensen P, Iversen H, Furuseth K. Implementing clinical guidelines in the treatment of diabetes mellitus in general practice. Evaluation of effort, process, and patient outcome related to implementation of a computer-based decision support system. Int J Technol Assess Health Care 2000;16:210-27.
120. McMahon GT, Gomes HE, Hickson Hohne S, Hu TM, Levine BA, Conlin PR. Web-based care management in patients with poorly controlled diabetes. Diabetes Care 2005;28:1624-9.
121. Meigs JB, Cagliero E, Dubey A, et al. A controlled trial of web-based diabetes disease management: the MGH diabetes primary care improvement project. Diabetes Care 2003;26:750-7.
122. Phillips LS, Ziemer I.C, Doyle JP, et al. An endocrinologist-supported intervention aimed at providers improves diabetes management in a primary care site: improving primary care of African Americans with diabetes (IPCAAD) 7. Diabetes Care 2005;28:2352-60.
123. Renders CM, Valk GD, Franse LV, Schellevis FG, van Eijk JT, van der Wal G. Long-term effectiveness of a quality improvement program for patients with type 2 diabetes in general practice. Diabetes Care 2001;24:1365-70.

124. Khunti K, Gadsby R, Millett C, Majeed A, Davies M. Quality of diabetes care in the UK: comparison of published quality-of-care reports with results of the Quality and Outcomes Framework for Diabetes. Diabet Med 2007;24:1436-41.
125. de Sonnaville JJ, Bouma M, Colly LP, Deville W, Wijkel D, Heine RJ. Sustained good glycaemic control in NIDDM patients by implementation of structured care in general practice: 2-year follow-up study. Diabetologia 1997;40:1334-40.
126. Prabhakaran D., Jeemon P., Mohanan P. P., et al. Management of acute coronary syndromes in secondary care settings in Kerala: Impact of a quality improvement programme. Natl Med J India 2008;21:62-6.
127. Ramachandran K, Husain N, Maikhuri R, et al. Impact of a comprehensive telephone-based disease management programme on quality-of-life in patients with heart failure. Natl Med J India 2007;20:67-73.
128. Hirsch IB, Goldberg HI, Ellsworth A, et al. A multifaceted intervention in support of diabetes treatment guidelines: a cont trial. Diabetes Res Clin Pract 2002;58:27-36.
129. Maislos M, Weisman D. Multidisciplinary approach to patients with poorly controlled type 2 diabetes mellitus: a prospective, randomized study. Acta Diabetol 2004;41:44-8.
130. Glazier RH, Bajcar J, Kennie NR, Willson K. A systematic review of interventions to improve diabetes care in socially disadvantaged populations. Diabetes Care 2006;29:1675-88.
131. Knight K, Badamgarav E, Henning JM, et al. A systematic review of diabetes disease management programs. Am J Manag Care 2005;11:242-50.
132. Shojania KG, Ranji SR, McDonald KM, et al. Effects of quality improvement strategies for type 2 diabetes on glycemic control: a meta-regression analysis. JAMA 2006;296:427-40.
133. Renders CM, Valk GD, Griffin S, Wagner EH, Eijk JT, Assendelft WJ. Interventions to improve the management of diabetes mellitus in primary care, outpatient and community settings. Cochrane database of systematic reviews (Online) 2001:CD001481.
134. Abegunde DO, Shengelia B, Luyten A, et al. Can non-physician health-care workers assess and manage cardiovascular risk in primary care? Bulletin of the World Health Organization 2007;85:432-40.
135. Bunting BA, Smith BH, Sutherland SE. The Asheville Project: clinical and economic outcomes of a community-based long-term medication therapy management program for hypertension and dyslipidemia. J Am Pharm Assoc (2003) 2008;48:23-31.
136. Ahern MM, Hendryx M. Avoidable hospitalizations for diabetes: comorbidity risks. Dis Manag 2007;10:347-55.
137. Alberti KG, Zimmet P, Shaw J. The metabolic syndrome--a new worldwide definition. Lancet 2005;366:1059-62.
138. Bohm M, Werner C, Jakobsen A, et al. Treating to protect: current cardiovascular treatment approaches and remaining needs. Medscape journal of medicine 2008;10 Suppl:S3.
139. Meigs JB, D'Agostino RB, Sr., Wilson PW, Cupples LA, Nathan DM, Singer DE. Risk variable clustering in the insulin resistance syndrome. The Framingham Offspring Study. Diabetes 1997;46:1594-600.
140. Kannel WB, McGee DL. Diabetes and cardiovascular disease. The Framingham study. Jama 1979;241:2035-8.
141. Haffner SM, Lehto S, Ronnemaa T, Pyorala K, Laakso M. Mortality from coronary heart disease in subjects with type 2 diabetes and in nondiabetic subjects with and without prior myocardial infarction. N Engl J Med 1998;339:229-34.
142. Donahoe SM, Stewart GC, McCabe CH, et al. Diabetes and Mortality Following Acute Coronary Syndromes. JAMA 2007;298:765-75.
143. Franco OH, Steyerberg EW, Hu FB, Mackenbach J, Nusselder W. Associations of Diabetes Mellitus With Total Life Expectancy and Life Expectancy With and Without Cardiovascular Disease. Arch Intern Med 2007;167:1145-51.
144. Malmberg K, Yusuf S, Gerstein HC, et al. Impact of diabetes on long-term prognosis in patients with unstable angina and non-Q-wave myocardial infarction: results of the OASIS (Organization to Assess Strategies for Ischemic Syndromes) Registry. Circulation 2000;102:1014-9.
145. Ramachandran A, Mary S, Yamuna A, Murugesan N, Snehalatha C. High prevalence of diabetes and cardiovascular risk factors associated with urbanization in India. Diabetes Care 2008;31:893-8.
146. Stratton IM, Adler AI, Neil HA, et al. Association of glycaemia with macrovascular and microvascular complications of type 2 diabetes (UKPDS 35): prospective observational study. Bmj 2000;321:405-12.
147. K. K. Shea, A. Shih, K. Davis. Health Care Opinion Leaders' Views on the Transparency of Health Care Quality and Price Information in the United States. In: The Commonwealth Fund; 2007.
148. Wagner EH, Austin BT, Von Korff M. Organizing care for patients with chronic illness. The Milbank quarterly 1996;74:511-44.

PROTOCOL

149. Wagner EH, Austin BT, Von Korff M. Improving outcomes in chronic illness. Managed care quarterly 1996;4:12-25.
150. Beckman JA, Creager MA, Libby P. Diabetes and atherosclerosis: epidemiology, pathophysiology, and management. JAMA 2002;287:2570-81.
151. Hobbs FD. Type-2 diabetes mellitus related cardiovascular risk: new options for interventions to reduce risk and treatment goals. Atheroscler Suppl 2006;7:29-32.
152. Moss SE, Klein R, Klein BE. Cause-specific mortality in a population-based study of diabetes. American journal of public health 1991;81:1158-62.
153. Geiss LS, Herman WM, Smith PJ. Mortality in Non-Insulin-Dependent Diabetes. In: National Diabetes Data Group, ed. Diabetes in America 2nd Edition: NIH & NIDDK; 1995:233-55.
154. World Health Organization. Definition, diagnosis and classification of diabetes mellitus and its complications; 1999.
155. OpenEpi: Open Source Epidemiologic Statistics for Public Health, Version 2.2.1. (Accessed October 29, 2008, at www.OpenEpi.com.)
156. Kelsey JL, Whittemore AS, Evans AS et al. Methods in observational epidemiology 2nd Edition. New York, NY: Oxford University Press; 1996.
157. Toobert DJ, Hampson SE, Glasgow RE. The summary of diabetes self-care activities measure: results from 7 studies and a revised scale. Diabetes Care 2000;23:943-50.
158. The EuroQol Group. EuroQol-a new facility for the measurement of health-related quality of life. Health Policy 1990;16:199-208.
159. Feeny D, Furlong W, Torrance G, et al. Multiattribute and single-attribute utility functions for the health utilities index mark 3 system. Medical Care 2002;40:113-28.
160. Bradley C. Diabetes treatment satisfaction questionnaire (DTSQ). Handbook of Psychology and Diabetes: A Guide to Psychological Measurement in Diabetes Research and Management:111–32.

PROTOCOL

17. SUPPLEMENTS/APPENDICES

17.1 Appendix 1: Patient Information Sheet and Informed Consent Form

Version 1.2; 10/Nov/2010
(Please give one copy to the participant and keep a copy for the Investigator)

Site Investigator: [Name of Site Investigator] **Hospital:** [Name of Hospital]

Study Title: Developing and Testing Integrated, Multi-factorial CVD Risk Reduction Delivery Strategies in South Asia

Coordinating Centre: Public Health Foundation India (PHFI), New Delhi

Sponsor Names: National Heart, Lung and Blood Institute, National Institutes of Health, USA
Ovations Chronic Disease Initiative, UnitedHealth Group, USA

Introduction:

You are invited to participate in a study for individuals with inadequate blood glucose (sugar) control and one or both of the following: high blood pressure and high cholesterol. These conditions increase your risk of complications of diabetes like heart attack, heart failure, stroke (brain injury caused by blocked blood flow), blood clots, kidney disease, eye, foot and skin problems and nerve damage affecting your sense of feeling. This study involves research to test whether a diabetes care intervention (making use of a patient care coordinator and a computerized decision-making system) to reduce heart disease risk works better than usual care.

Your participation is entirely voluntary (your choice). To help you make your decision, please read this information sheet. You are free to discuss the contents of this document with members of your family or your physician. You may take as much time as you like to consider whether or not to take part in the study. If you choose not to take part, your current or future care will not be affected. If you agree to take part, you are free to withdraw from the study at any time without penalty or loss of benefits, to which you are otherwise entitled.

Once you understand what is involved in the study and you wish to participate, you, along with your study doctor, will be asked to sign the consent form. If you have questions at any time during the research study, you should feel free to ask them and obtain answers to your questions. You are not giving up any of your legal rights by volunteering for this research study or by signing this consent form.

Study Purpose:

The purpose of this research study is to find out whether patient care monitored by an additional staff member (care coordinator) and better glucose, blood pressure, and cholesterol control with computer assistance (also called decision support system), will reduce the risk of complications, like heart disease and stroke, in diabetes patients. A total of 1120 participants across 8 to 10 clinic sites in South Asia will be involved in this study for 2.5 to 3 years. Approximately 140 participants will be selected from each site to participate in the study.

PROTOCOL

You will be randomly assigned by the computer to one of the two groups: (1) continuation of the usual diabetes care, and (2) diabetes management with frequent monitoring by an additional staff (care coordinator) and computer-assisted decision support (also called decision support system, DSS). The DSS will help the doctors by giving suggestions and reminders and also remind patients about appointments and visits. The care coordinator will help patients follow the physician's advice. The treatments given to patients in both the groups will remain the same. However, the care coordinator and DSS group participants will receive more active help with managing their diabetes.

There is a 50:50 chance of being assigned to either of the two groups (like the flip of a coin). This decision is made centrally, and the group each person receives is decided by chance only ("random allocation").

During the study:

If you agree to participate, you will be seen at the beginning of the study on 3 occasions. The first 2 visits will be to see if you are eligible for the study and the 3rd visit is for baseline evaluation and assigning you to your treatment group. At the first visit, you will have a brief medical history taken. If you still qualify for the study, you will need to come back within 1 week after fasting overnight for the second visit to do blood tests to check your blood sugar and fat levels, and kidney and liver function. The amount of blood taken will be approximately 2 teaspoons (10 ml). A urine test for kidney function and pregnancy test (if applicable) will also be done. At the third visit, depending on the results of the previous tests, you will be informed whether or not you qualify to continue on with the study. If you qualify, we will do a full history and physical exam, and other questionnaires about your care. Afterwards, we will provide you your group assignment.

Subsequent follow-up clinic visits: The expected time that you will be followed-up for this trial is 2.5 years. Those assigned to getting usual care for diabetes will be seen as per routine care, with an additional visit every 12 months for study-related tests. Those assigned to the group with the care coordinator and DSS will be called back to the clinic every 3 months. At these visits, we will obtain blood tests and do a general physical exam to monitor your diabetes control. At every 12-monthly visit, we will do a few additional questionnaires and some extra tests.

Risks and Discomforts:

This study will not cause you any harm or discomfort more than your existing care for diabetes. When blood is drawn for lab tests during the study, there is a possibility of bruising, discomfort from the needle puncture, and infection. At present, there are no other anticipated risks or side effects.

You may not participate if you are a woman of childbearing potential who is not using an approved birth control method, if you are pregnant, or if you are nursing. If you become pregnant during the course of the study, you should notify the study doctor of this fact immediately, since diabetes during pregnancy requires more careful management, different from the standard care planned in this study, which is for non-pregnant adults.

PROTOCOL

Benefits:

The benefits to you for participating in this study may be the same as your existing clinical care for diabetes. You may become more aware of your condition and treatments with increased clinic visits. However, we do not know which of the two study groups is better, so you may receive no direct benefit from participating in this study. Information gathered from this study may be helpful to the future management of heart disease risk among patients.

Participation in this study will be at no cost to you. There will be no monetary compensation for your participation.

Alternative Procedures:

Different plans to care for diabetes patients have been assessed on an individual basis. Some diabetic clinics use additional staff for patient follow-up, which is similar to the care coordinator. Some hospitals make use of electronic health records for collecting patient information, which is similar to the decision-support software. However, whether these plans of using a care coordinator and DSS are useful, is not known. This study aims to bring these care plans together into one comprehensive care package to reduce the risk of heart disease.

Confidentiality:

During your participation in this clinical study, the research staff will collect information related to your health. Your collected information will be stored locally and sent to the Research Coordinating Centre (RCC) in New Delhi who are performing this study. Your information will be kept in a secure location with access limited to authorized personnel only. The RCC will store and process your information with electronic data processing systems. In the electronic database, your information will be identified only with a code number. At the end of the study, all personal identifiers will be destroyed; the remaining information will be stored for 3 years and securely disposed thereafter. Blood samples for long-term storage will only be identified with a code number and will be stored at a central laboratory within India. The stored blood samples will be accessed only by the research team for later analyses.

By signing this informed consent form, you are agreeing to allow the study monitors, government regulatory agencies, and Research Ethics Board to examine your medical records. Your name will be kept confidential to the extent allowed by law, and you will not be identified personally in any presentations or reports dealing with this research. When the results of the study are published, your identity will not be revealed.

Withdrawal of Participation:

Your decision whether or not to participate in this research study is completely voluntary. In addition, you may withdraw from the study at any time for any reason. If you decide to withdraw from the study before the finish, you will be asked to provide the reason(s) for withdrawal, but you have the option not to provide the reason(s). There will be no penalty or loss of benefits to you if you decide not to participate or decide to withdraw from the study. Your participation can also be stopped by your study doctor or the study sponsor for the following reasons:

- Any undesirable effect appears

PROTOCOL

- If you do not comply with the requirements of the study
- If your study doctor or the sponsor has the opinion that it would be in your best interest to withdraw from the study
- If the sponsor stops the study

Inquiries/Questions:

If you have any questions about the research, develop a research-related problem, or note a change in your condition, you should contact the Site Investigator, [Name of Site Investigator], at [phone number and address].

Should you have any questions regarding your rights as a research participant, you may contact the Institutional Review Board of [name of local institution] at [phone number and address for the IRB].

PROTOCOL

CONSENT FORM

Version 1.2; 10/Nov/2010
(Please give one copy to the participant and keep a copy for the Investigator)

Study Title: Developing and Testing Integrated, Multi-factorial CVD Risk Reduction Delivery Strategies in
South Asia - the CARRS Translation Trial

Participant: Mr/Mrs/Miss __________ ______________ ___ / ___ /______; _____ years
First Name Last Name Birth Date (dd/mm/yyyy); Age

Please read the following before putting your signature below:	Place [X]
(i) I confirm that I have read and understood the participant information sheet for the above study and have had the opportunity to ask questions.	[ ]
(ii) I understand that my participation in the study is voluntary and that I am free to withdraw at any time, without giving any reason, without my medical care or legal rights being affected.	[ ]
(iii) I understand that the Sponsor of the study, others working on the Sponsor's behalf, the Ethics Committee, and the regulatory authorities will not need my permission to look at my health records, both for the current study and any further research that may be conducted in relation to it, even if I stop taking part in the study. I understand that my identity will not be revealed in any information released to third parties or published.	[ ]
(iv) I agree not to restrict the use of any of my information or results that arise from this study provided such a use is only for scientific purpose(s).	[ ]
(v) I have been given a copy of the information sheet and consent form to keep. By signing this form I have not given up my legal rights.	[ ]

____ Date: ___ / ___ /_______
Signature/Thumb Impression of Participant

____
Printed Name of Participant

____ Date: ___ / ___ /_______
Signature of Investigator

____
Printed Name of Investigator

____ Date: ___ / ___ /_______
Signature of the Witness (or Legal Representative)

____
Printed Name of Witness (or Legal Representative)

PROTOCOL

17.2 Appendix 2: Physician Interview Information Sheet and Consent Form

Version 1.0 dated 6 January 2010

(Please give one copy to the physician and keep a copy for the Interviewer)

Study Title: Developing and Testing Integrated, Multi-factorial CVD Risk Reduction Delivery Strategies in South Asia

Principal Investigator: Dr. D Prabhakaran, MD, MSc, DM

Coordinating Centre: Public Health Foundation India (PHFI), New Delhi

Sponsor Names: National Heart, Lung and Blood Institute, National Institutes of Health, USA
Ovations Chronic Disease Initiative, UnitedHealth Group, USA

Introduction and Purpose:

You are presently a study physician for the CARRS Translation Trial. The trial is testing the effectiveness and sustainability of a diabetes management intervention that uses multi-faceted strategies (non-physician care coordinator and clinical decision-support software) to reduce the cardiovascular disease risk among Type 2 diabetes patients. We are requesting your participation in 4 interviews throughout the trial about your diabetes care practice and the performance and sustainability of the intervention. Your feedback will help improve the intervention which is intended for scale-up in India to reduce the cardiovascular morbidity and mortality among diabetes patients. A maximum of 3 providers will be interviewed at your site.

This form is designed to tell you everything you need to understand before you decide to consent (agree) to be in the study or not to be in the study. It is entirely your choice. If you decide to take part, you can change your mind later on and withdraw from the research study. The decision to join or not join the research study will not cause you to lose any benefits.

Procedures:

If you agree to participate, you will be taking part in 4 interviews over the course of the trial (at baseline and annually afterwards). Each interview will last approximately 30 minutes and will be conducted at your office, clinical facility or another mutually agreed upon location. During the baseline interview, the questions will be about your present practice in diabetes care and the challenges and successes you have in patient management; and also your views on the feasibility of the intervention. During the 3 follow-up annual interviews, you will be asked about the intervention's progress and the effects on patient management, and your views on its sustainability. The interview will be tape-recorded with your consent, and the recordings will be safely stored to protect your privacy. Your name will not be used in the recording.

Risks and Discomforts:

There are no foreseeable risks or discomforts associated with this study. You may stop the study at any time.

Benefits:

Your feedback from the interview will be used to improve the diabetes management intervention during the course of the trial. After the trial, the goal is that the tested intervention strategies can be scaled-up for use in other clinics to improve diabetes care in India and globally.

Compensation:

Your participation is completely voluntary. No material compensation will be provided for your participation. Your help with this study is greatly appreciated.

PROTOCOL

Confidentiality:

A study number rather than your name will be used on data collected. The code that links the study number to your name will be kept in a secure place, available only to me. All research records and recorded interviews will be kept in a secure, pass-word protected computer. Your name and other facts that might identify you will not appear in study results. Any shared data will not include any identifiable information. Audio-recordings of the interview will be destroyed at the end of the study. People other than those doing the study may look at study records: Agencies and committees that make rules and policy about how research is done have the right to review these records, as well as agencies that pay for the study.

Withdrawal of Participation:

Participating in the interviews is voluntary, and you may leave the study at any time without penalty. This decision will not affect in any way your current or future care or any other benefits to which you are otherwise entitled. You may also refuse to answer any questions that you do not want to during the interview.

Inquiries/Questions:

If you have questions about your rights as a research subject or if you have questions, concerns or complaints about the research, you may contact COE-CARRS: Address: C-1/52, 2nd floor, Safdarjung Development Area, New Delhi 110 016; Phone: 91.11.26850117/18; or email CARRStrial@ccdcindia.org.

If you have questions about your rights as a research participant or if you have questions, concerns or complaints about the research, you may also contact the Public Health Foundation Institutional Ethics Committee: Address- Balbir Saxena Marg, Ground Floor, Hauz Khas, New Delhi 110 016; Phone: 91.11.46046000 (Extention - 259 & 255); or email trc-iec@phfi.org.

Consent:

Do not sign this consent form unless you have had a chance to ask questions and get answers that make sense to you. Nothing in this form can make you give up any legal rights. By signing this form you will not give up any legal rights.

You will receive a copy of this consent form to keep.
Please sign below if you agree to participate in this study.

____

Name of Physician-Participant

____ _______________

Signature of Physician-Participant Date

____ _______________

Interviewer Date

PROTOCOL

17.3 Appendix 3: Cardiovascular Disease (CVD) Risk Management Algorithm-Guidelines

(As of 23 October 2010)

General Guideline: Attempt to use the lowest-cost medication, but do not change medications.

1) Glycemia Control:
At Visit 3 (Baseline/Randomization), the physician will assess the labs to determine the glycemia control strategy for the intervention participant. Treatment will be increased since the participants already have HbA1c> 8%.

- The physician and the care coordinator must update the treatment for follow-up.
- The patient will also be supplied instructions and expectations with the change of medications.

Table 1: Decision Support Table for Glycemia Control

		Fasting Blood Glucose (mg/dl)		
Hba1c (%)		**<110**	**110-130**	**>130**
	**<7**	**Good control**<br>Continue with existing regimen	**Fair control (maybe inconsistent HbA1c and FBG)**<br>Reinforce lifestyle counseling	**Likely poor control (inconsistent HbA1c and FBG)**<br>Check post-prandial blood glucose levels<br>Increase* treatment (Take steps to reduce FBG)
	**7-8**	**Likely poor control**<br>-Re-check HbA1c<br>-Check post-prandial blood glucose levels<br>-If high, mealtime interventions[$]	**Insufficient control**<br>Increase* treatment<br>Check post-prandial glucose levels and control	**Poor control**<br>Greater** increase in treatment<br>Check post-prandial glucose levels and control
	**>8**	**Likely poor control**<br>-Re-check HbA1c<br>-Check post-prandial blood glucose levels<br>-If high, mealtime interventions[$]	**Poor control**<br>Greater** increase in treatment<br>Check post-prandial glucose levels and control	**Very poor control**<br>Greater** increase in treatment<br>Check post-prandial glucose levels and control

***Increase** = 1 of the following possible changes:

- Increase in Metformin dose by 500 mg
- Increase in SU dose by 25% of maximum dose of the sulfonylurea in use[#]
- Increase in pioglitazone by 15 mg *(Rosiglitazone not used widely)*

[$] Consider adding one increment of alpha glucosidase inhibitors: 25 mg of acarbose; 25 mg of miglitol; 0.2 mg of voglibose
[#] 5 mg of glibenclamide; 2 mg of glimepiride; 80 mg of gliclazide; 30 mg of modified release gliclazide

If starting first OHA: for those BMI<23, start with SU; for those BMI>23, start with Metformin

****Greater Increase** = 2 of the following possible changes (2 of the same OR 2 different):

- Increase in Metformin dose by 500 mg
- Increase in SU dose by 25% of maximum dose of the sulfonylurea in use[#]
- 33% increase in maximum pioglitazone

[#] 5 mg of glibenclamide; 2 mg of glimepiride; 80 mg of gliclazide; 30 mg of modified release gliclazide

Note: These main OHA are suggested because DPP-4 inhibitors (Sitagliptin) is not used widely, and GLP-1 analogs (Exenatide) is limited to wealthier patients.

If attained maximal SU and maximal metformin dosages:

- And between 7-8% HbA1c → Add 3rd OHA (glitazone suggested); **If attained maximal triple OHA treatment** → start insulin therapy **(see below for Insulin instructions)**
- And ≥8.0% HbA1c → Add insulin, basal bedtime dose **(see below for Insulin instructions)**

Use of Insulin for participants on maximal oral therapy:

1. Add bedtime NPH dose (10 units or .2 U/kg/day); Instruct on SHBGM
2. After 1 week, Review FBG of SHBGM (target: 80-110)
   a. If too low → reduce NPH dose
   b. If too high → increase NPH dose. If insulin dose >.5 U/kg, add AM NPH (10 units or .2 U/kg/day)
      i. Titrate as needed

PROTOCOL

	ii. If HbA1c remains high despite adequate pre-prandial glucose control, check post-prandial levels and if they are high → stop secretagogues (SU, glinides,) and either switch to biphasic or add prandial insulins

---

2) Blood pressure control:
At Visit 2 (Baseline/Randomization), the physician will assess blood pressure readings to determine the blood pressure control strategy for the intervention participant.

**Table 2:** Decision Support Table for blood pressure control					
	Systolic BP				
Diastolic BP		<130	130-140	140-160	>160
	<80	**Good control**<br><br>Continue with existing regimen	**Fair control**<br>-Re-check BP at next visit<br>-Reinforce lifestyle counseling	**Likely poor control**<br>-Systolic hypertension<br>-Increase treatment (1-2 increment, Consider CCB)	**Urgent**<br>-Immediate and greater medication (at least 2 increments, consider CCB)
	80-90	**Likely poor control**<br><br>-Re-check BP<br>-Reinforce lifestyle counseling	**Insufficient control**<br><br>Increase treatment (1 increment)	**Poor control**<br><br>Increase treatment (1-2 increments)	**Urgent**<br>-Immediate and greater medication (at least 2 increments, consider CCB)
	>90	**Likely poor control**<br><br>Increase treatment (1 increment)	**Poor control**<br><br>Increase treatment (1 increment)	**Very poor control**<br><br>Increase treatment (1-2 increments)<br><br>-earlier follow-up, in 4 weeks	**Very urgent**<br>-Immediate and greater medication (at least 2 increments)<br><br>-earlier follow-up, every 2 weeks

BP medication increment units:
1st line: ACE-I (Enalapril 5mg) → if cough (assess in 2 weeks), use ARB
ARB (Losartan 25 mg)
Thiazide (HCTZ 12.5 mg)
HCTZ-like (Indapamide 1.5 mg)
CCB (amlodipine 5 mg)
B-blockers (atenolol 50 mg, metoprolol SR 50 mg)

Increment units of other anti-hypertensive medication:
ACE-I: ramipril (5 mg); lisinopril (5mg); perindopril (2 mg); quinaril (2 mg): captopril (25 mg)
ARB: telmisartan (20 mg); olmisartan (10 mg); candesartan (4 mg); irbesartan (150 mg)
CCB: diltiazem (30 mg); verapamil (40 mg)
B-blockers: metoprolol (25 mg)
Alpha blockers: Slow release prazosin (2.5 mg)
Centrally active agents: clonidine (0.1mg); alpha methyl dopa (250 mg)

If initiating Ace-Inhibitor and if baseline: *Cr > 1.4 mg/dL Women; Cr> 1.5 mg/dL Men* **OR** *K+ > 5 meq/dL*
→ Follow-up K+ and Cr at *2-4 weeks*. Otherwise, repeat K+ and Cr at 3 monthly visit (for those initiated)

PROTOCOL

3) Lipid control:
At Visit 2, the physician will assess blood pressure readings to determine the blood pressure control strategy for the intervention participant.

**Table 3:** Decision Support Table for lipid control			
	LDL cholesterol level (mg/dL)		
With history of previous CVD event	**<70**	**70-100**	**>100**
Without history of previous CVD event	**<100**	**100-130**	**>130**
	**Good control**<br><br>Continue with existing regimen	**Poor control**<br><br>Increase treatment (1 increment)	**Very poor control**<br><br>Greater increase in treatment (2 increments)

Lipid medication increment units:
1st line: Atorvastatin 10 mg **less costly and small starting dose

Simvastatin 20 mg
Rosuvastatin 10 mg

- **If initiating lipid medication, follow-up LFTs in 3 months.**
- **For fibrates, will provide guidelines once ACCORD** cross-over study results are published by Spring 2010.

<u>**Screenings for individuals with diabetes**</u>
- Annual foot exam (with monofilament test)
- Annual eye exam (with dilated pupil fundoscopy)
- Annual microalbuminuria test (urine analysis)
- Annual ECG

<u>**Smoking Cessation**</u>
- Intensive and regular counseling for smoking cessation for those who are smoking
- Regular follow-up for those who have quit smoking

<u>**Lifestyle Advice**</u>
- Regular follow-up and counseling at every visit regarding diet, exercise, medication adherence, foot care, and other social aspects (i.e. stress, support)

<u>**Aspirin Use, 75 mg-162 mg /day**</u>
NOTE: If documented aspirin allergy or contraindications, use clopidogrel (75 mg/day)

Primary Prevention: For diabetes patients with increased CVD risk (10-year risk > 10%):
- Male >50 years or Female >60 years (but less <70 years) with at least 1 additional risk factor:
  - family history of CVD
  - Hypertension
  - Smoking
  - dyslipidemia, or
  - albumiuria
- If between 70-79, consider lipid and blood pressure-lowering therapies first, then reassess whether aspirin adds additional net benefit.
- If Male <50 years or Female<60 years with CVD risk factors, follow clinical judgment.

Secondary prevention: For all diabetes patients with history of CVD
Up to 1 year after ACS: combination aspirin (75-162 mg/day) and clopidogrel (75 mg/day)

Other reference: Antithrombotic Trialists' (ATT) Collaboration. Aspirin in the primary and secondary prevention of vascular disease: collaborative meta-analysis of individual participant data from randomised trials. Lancet. 2009 May 30; 373(9678): 1849–1860.

PROTOCOL

17.4 Appendix 4: Subcommittee Responsibilities

The designated Subcommittees will be responsible for monitoring portions of the trial during data collection, data analysis, and dissemination of results.

Data Management and Quality Control Subcommittee
This subcommittee developed the Decision Support Software, the central Research Coordinating Center data management software, and the programs for the quality control checks of collected data.

The subcommittee will establish quality assurance criteria under which the Clinical Sites and the Research Coordinating Center are expected to perform. The subcommittee will review all aspects of quality control monitoring in areas such as protocol execution, laboratory standardization (collection, processing, and storage of body fluid samples), data collection (quality, timeliness, completeness) and data entry/management. Any deviations from expected performance levels will be relayed to this subcommittee by the RCC and/or the Clinical Sites. Personnel from this committee will conduct at least 1 visit a year to all the Clinical Sites for quality assurance checks; the visit reports will be reviewed by the Subcommittee to determine what action to take.

Publication, Presentation and Ancillary Study Subcommittee
This subcommittee develops the policies and guidelines by which CARRS Translation Trial investigators will conduct analyses, write papers, make presentations, and from which ancillary studies using the CARRS network studies as a platform may be approved. Other responsibilities include: approving all analyses, papers, presentations, and proposals; soliciting writing group personnel; and monitoring the progress of all proposed papers to ensure prompt completion and publication. The subcommittee ensures that all proposals are processed in a timely manner.

Endpoint Adjudication Subcommittee
This subcommittee will convene on a regular basis to classify the occurrence of hard clinical endpoints in a masked fashion and monitor event classification by Sites for quality control.
Definitions to be used for classification of clinical events will be provided by the Steering Committee and included in the Manual of Procedures for the Endpoint Adjudication Subcommittee.

Qualitative Methods Subcommittee
This subcommittee developed the qualitative evaluation tools, training manual for evaluators, and the data analysis plan. This subcommittee monitors the progress of this evaluation component and provides regular reports to the Steering Committee.

Cost-Effectiveness Subcommittee
This subcommittee established the protocol for measuring cost-effectiveness of the trial, developed the training guide for assessors, and the data analysis plan. This subcommittee monitors the progress of this evaluation component and provides regular reports to the Steering Committee.

PROTOCOL

17.5 Appendix 5: Case Report FORMS (CRFs)

See attachments:

1.	Form A – Screening Part 1	v1.2-17JUL2010
2.	Form B – Screening Part 2	v1.2-17JUL2010
3.	Form C – Baseline_Randomization	v1.3-29JUL2010
4.	Form D – 3 monthly Visit_Intervention	v1.2-17JUL2010
5.	Form E – Follow-up 12 monthly_All	v1.3-29JUL2010
6.	Form F – Close-out_All	v1.3-29JUL2010
7.	Form G – Eye Exam	v1.2-17JUL2010
8.	Form I.c – Intermediate Visit_Control	v1.2-17JUL2010
9.	Form I.i – Intermediate Visit_Intervention	v1.2-17JUL2010
10.	Form K – Interview Guide_Physician	v1.2-17JUL2010
11.	Form Z – Intervention Management Plan	v1.2-17JUL2010

PROTOCOL
