# Supplementary material for: Effect of a multicomponent quality improvement strategy on sustained achievement of diabetes care goals and macrovascular and microvascular complications in South Asia at 6.5 years follow-up: Post hoc analyses of the CARRS randomized clinical trial
Source: PLoS Med. 2024 Jun 3;21(6):e1004335. doi: 10.1371/journal.pmed.1004335 (PMC11198027; doi:10.1371/journal.pmed.1004335)
Supplement: S1 Supporting information — Fig A. Mean change in HbA1c, SBP, DBP, and LDL-c at baseline, 2.5 years, and 6.5 years by treatment assignment. Table A. Multiple and single risk factor control at baseline, 2.5 years, and 6.5 years by treatment assignment. Fig B. Multiple and single risk factor control at baseline, 2.5 years, and 6.5 years by treatment assignment and sites that continued versus discontinued active intervention. Fig C. Mean changes in HbA1c, SBP, DBP, and LDL-c at baseline, 2.5 years, and 6.5 years by treatment assignment and sites that continued versus discontinued active intervention. Table B. Relative risks and risk differences for composite and individual macrovascular and microvascular endpoints. Table C. Relative risks and risk differences for composite and individual macrovascular and microvascular endpoints—Sensitivity analysis limited to sites that continued the intervention. Table D. Relative risks and risk differences for composite and individual macrovascular and microvascular endpoints—Sensitivity analysis limited to sites that discontinued the intervention at 2.5 years. Fig D. Intervention effects on composite major adverse cardiovascular outcomes by baseline socioeconomic and clinical characteristics. Fig E. Intervention effects on multiple risk factor control by baseline socioeconomic and clinical characteristics Intervention effects on composite first macrovascular outcomes by baseline socioeconomic and clinical characteristics. Table E. Serious adverse events by treatment assignment. Table F. Post hoc sensitivity analysis examining multiple and single risk factor control using alternative statistical approaches. Table G. Baseline demographic and clinical characteristics of patients at sites that continued versus discontinued the intervention. Table H. Sensitivity analysis examining multiple and single risk factor control using alternative statistical approaches. Table I. Sensitivity analysis examining multiple risk factor control using controlled imputation to acco [file pmed.1004335.s004.docx]

**Supplementary Online Content**

**Supplementary Tables and Figures**

**Fig A.** Mean change in HbA1c, SBP, DBP, and LDL-c at baseline, 2.5 years, and 6.5 years by treatment assignment

**Table A.** Multiple and single risk factor control at baseline, 2.5 years, and 6.5 years by treatment assignment

**Fig B.** Multiple and single risk factor control at baseline, 2.5 years, and 6.5 years by treatment assignment and sites that continued versus discontinued active intervention

**Fig C.** Mean changes in HbA1c, SBP, DBP, and LDL-c at baseline, 2.5 years, and 6.5 years by treatment assignment and sites that continued versus discontinued active intervention

**Table B.** Relative Risks and Risk Differences for Composite and Individual Macrovascular and Microvascular endpoints

**Table C.** Relative Risks and Risk Differences for Composite and Individual Macrovascular and Microvascular endpoints – Sensitivity Analysis limited to sites that continued the intervention

**Table D.** Relative Risks and Risk Differences for Composite and Individual Macrovascular and Microvascular endpoints – Sensitivity Analysis limited to sites that discontinued the intervention at 2.5 years

**Fig D:** Intervention Effects on Composite Major Adverse Cardiovascular Outcomes by Baseline Socioeconomic and Clinical Characteristics

**Fig E:** Intervention Effects on Multiple Risk Factor Control by Baseline Socioeconomic and Clinical Characteristics

**Table E: Serious Adverse Events by Treatment Assignment**

**Table F:** Post Hoc Sensitivity Analysis examining Multiple and Single Risk Factor Control using Alternative Statistical Approaches

**Table G**: Baseline demographic and clinical characteristics of patients at sites that continued versus discontinued the intervention

**Table H**: Sensitivity Analysis examining Multiple and Single Risk Factor Control using Alternative Statistical Approaches

**Table I**: Sensitivity Analysis examining Multiple Risk Factor Control using Controlled Imputation to Account for Non-random Missing Data

**Table J**: Sensitivity Analysis examining Multiple and Single Risk Factor Control Adjusting for Clustering by Site Care Coordinato

This supplementary material has been provided by the authors to give readers additional information about their work.

**Fig A**. Mean change in HbA1c, SBP, DBP, and LDL-c at baseline, 2.5 years, and 6.5 years by treatment assignment


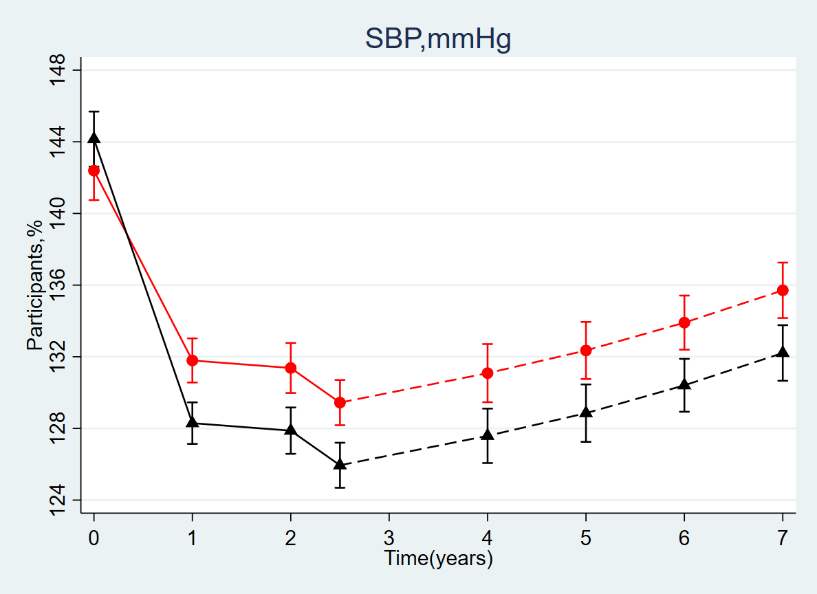

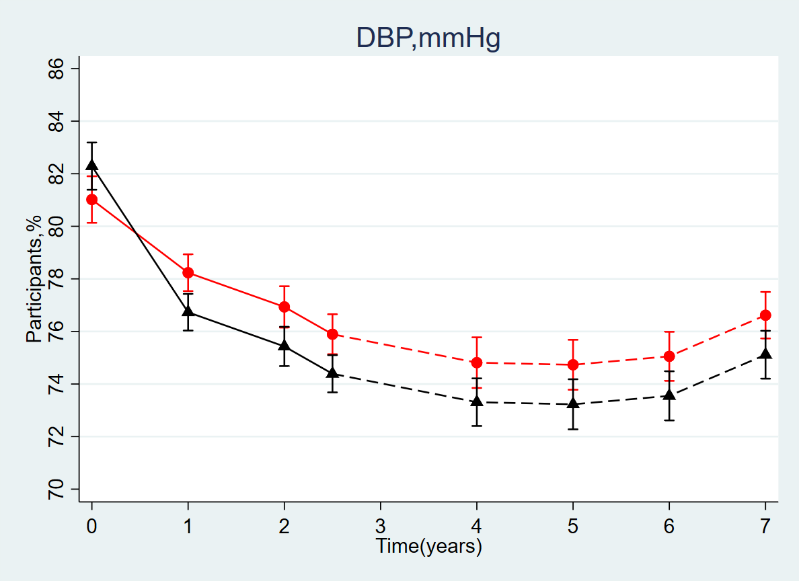

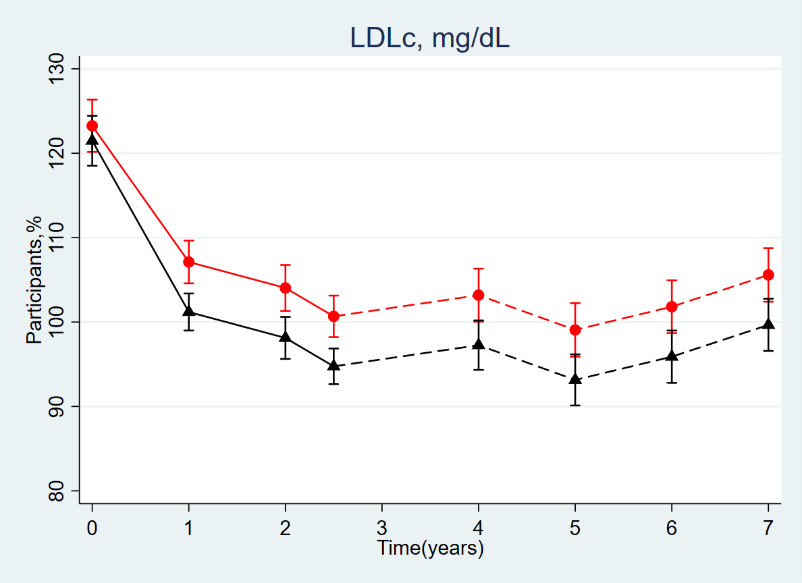

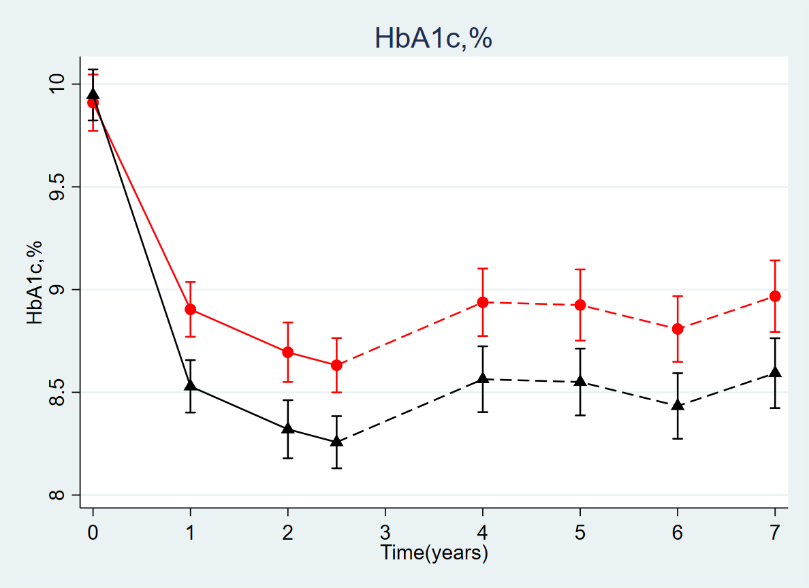


Difference (95%CI) = -0.38 (-0.53, -0.23)

Difference (95%CI) = -3.40 (-4.82, -1.97)

Difference (95%CI) = -1.46 (-2.25, -0.67)

Difference (95%CI) = -6.04 (-8.74, -3.35)

**(Figure: S1.A)**

**(Figure: S1.B)**

**(Figure: S1.C)**

| I, n | 575 | 499 | 319 | 478 | 215 | 200 | 244 | 251 |
| --- | --- | --- | --- | --- | --- | --- | --- | --- |
| UC, n | 571 | 504 | 310 | 469 | 202 | 200 | 275 | 272 |

| I, n | 575 | 499 | 319 | 472 | 215 | 201 | 245 | 254 |
| --- | --- | --- | --- | --- | --- | --- | --- | --- |
| UC, n | 571 | 505 | 311 | 464 | 204 | 200 | 272 | 275 |

| I, n | 575 | 499 | 319 | 472 | 215 | 201 | 245 | 254 |
| --- | --- | --- | --- | --- | --- | --- | --- | --- |
| UC, n | 571 | 505 | 311 | 464 | 204 | 200 | 272 | 275 |

| I, n | 575 | 499 | 319 | 473 | 215 | 200 | 245 | 249 |
| --- | --- | --- | --- | --- | --- | --- | --- | --- |
| UC, n | 504 | 312 | 471 | 194 | 203 | 274 | 271 | 504 |

**(Figure: S1.D)**

Intervention Δ black font; Usual Care red font•

Data reported are mean values for hbA1c, SBP, LDLc and DBP over the time period

Overall mean difference was obtained via linear regression models using generalized estimating equations. Estimates combine all nonmissing values collected at baseline and year 1, 2, 2.5, 4, 5, 6, and 7. Error bars indicate 95% CIs. Model terms included treatment group, time, respective baseline value, age, gender and site.

Data reported are mean values for HbA1c, SBP, LDLc and DBP over the time period

Dashed lines show measures and trends between 2.5 years and 6.5 years post-randomization

DBP, diastolic blood pressure; HbA1c, glycated hemoglobin; LDLc, low-density lipoprotein cholesterol; SBP, systolic blood pressure

**Table A**. Multiple and single risk factor control at baseline, 2.5 years, and 6.5 years by treatment assignment

|  |  | **Baseline** | **12m** | **24m** | **36m** | **48m** | **60m** | **72m** | **≥84m** | Relative risk* (95% CI) | Risk difference* % (95% CI) | Relative risk# (95% CI) | Risk difference# % (95% CI) |
| --- | --- | --- | --- | --- | --- | --- | --- | --- | --- | --- | --- | --- | --- |
| **Multiple risk factor control** | **I** | 0 | 14.9  (12.1, 17.7) | 17.4  (13.7, 21) | 21.8  (18.4, 25.1) | 15.2  (11.4, 19) | 18.7  (14.4, 23) | 19.2  (15.3, 23.1) | 14.1  (10.6, 17.6) | 1.77 (1.45, 2.17) | 7.6 (5, 10.3) | 1.77 (1.45, 2.16) | 7.6 (4.9, 10.2) |
|  | **UC** | 0 | 8.4  (6.7, 10.2) | 9.8  (7.6, 12) | 12.3  (10, 14.6) | 8.6  (6.2, 10.9) | 10.6  (7.9, 13.3) | 10.8  (8.3, 13.3) | 7.9  (5.7, 10.2) |  |  |  |  |
| **HbA1c <8%** | **I** | 0 | 18.7  (15.7, 21.7) | 20.9  (17.1, 24.7) | 24.1  (20.7, 27.5) | 16.8  (12.9, 20.7) | 21.2  (16.8, 25.6) | 21.2  (17.2, 25.2) | 19.4  (15.4, 23.4) | 1.60 (1.34, 1.92) | 7.7 (4.8, 10.7) | 1.59 (1.33, 1.91) | 7.6 (4.7, 10.6) |
|  | **UC** | 0 | 11.7  (9.5, 13.8) | 13.0  (10.5, 15.6) | 15.1  (12.5, 17.6) | 10.5  (7.8, 13.1) | 13.2  (10.2, 16.2) | 13.2  (10.5, 15.9) | 12.1  (9.3, 14.9) |  |  |  |  |
| **BP <130/80 mm Hg** | **I** | 16.2 (13.4, 19.4) | 47.8  (44.3, 51.3) | 52.5  (48.4, 56.6) | 55.9  (52.2, 59.6) | 55.5  (50, 61) | 49.5  (44.4, 54.7) | 47.8  (42.8, 52.8) | 40.4  (35.7, 45.1) | 1.20 (1.11, 1.30) | 8.5 (4.9, 12.1) | 1.20 (1.11, 1.3) | 8.3 (4.7, 11.8) |
|  | **UC** | 20.3 (17.2, 23.8) | 39.7  (36.5, 42.9) | 43.6  (39.8, 47.4) | 46.5  (43.1, 49.9) | 46.1  (41.3, 50.9) | 41.2  (36.7, 45.6) | 39.7  (35.5, 43.9) | 33.6  (29.5, 37.6) |  |  |  |  |
| **LDLc <100 mg/dL (<70 mg/dL with history of CVD)** | **I** | 27.0 (23.5, 30.7) | 51.6  (48.2, 55) | 59.5  (55.4, 63.6) | 63.0  (59.4, 66.7) | 60.2  (55.2, 65.3) | 63.2  (58, 68.4) | 61.1  (56.5, 65.6) | 55.7  (50.9, 60.5) | 1.15 (1.07, 1.23) | 7.5 (3.7, 11.4) | 1.15 (1.07, 1.23) | 7.6 (3.8, 11.5) |
|  | **UC** | 24.2 (20.8, 27.9) | 44.9  (41.7, 48.2) | 51.8  (47.8, 55.8) | 54.9  (51.3, 58.5) | 52.5  (48, 57) | 55.1  (50.4, 59.7) | 53.2  (49.1, 57.3) | 48.5  (44.2, 52.8) |  |  |  |  |

Multiple risk factor control defined as: HbA1c <8% & either BP <130/80 mmHg and/or LDLc <100 mg/dL [<70 mg/dL with history of CVD]

Numbers reported are % (95% CI)

Relative risk and risk difference calculated using log-binomial regression or poisson regression

* Models adjusted for site and respective baseline values

# Models adjusted for age, sex, site and respective baseline values

I, Intervention; UC, Usual care; BP, blood pressure; HbA1c, glycated hemoglobin; LDLc, low-density lipoprotein cholesterol; SBP, systolic blood pressure

**Fig B.** Multiple and single risk factor control at baseline, 2.5 years, and 6.5 years by treatment assignment and sites that continued versus discontinued active intervention


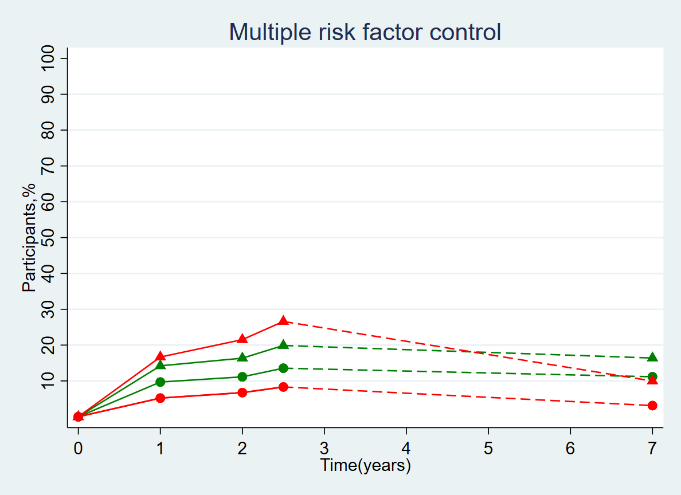

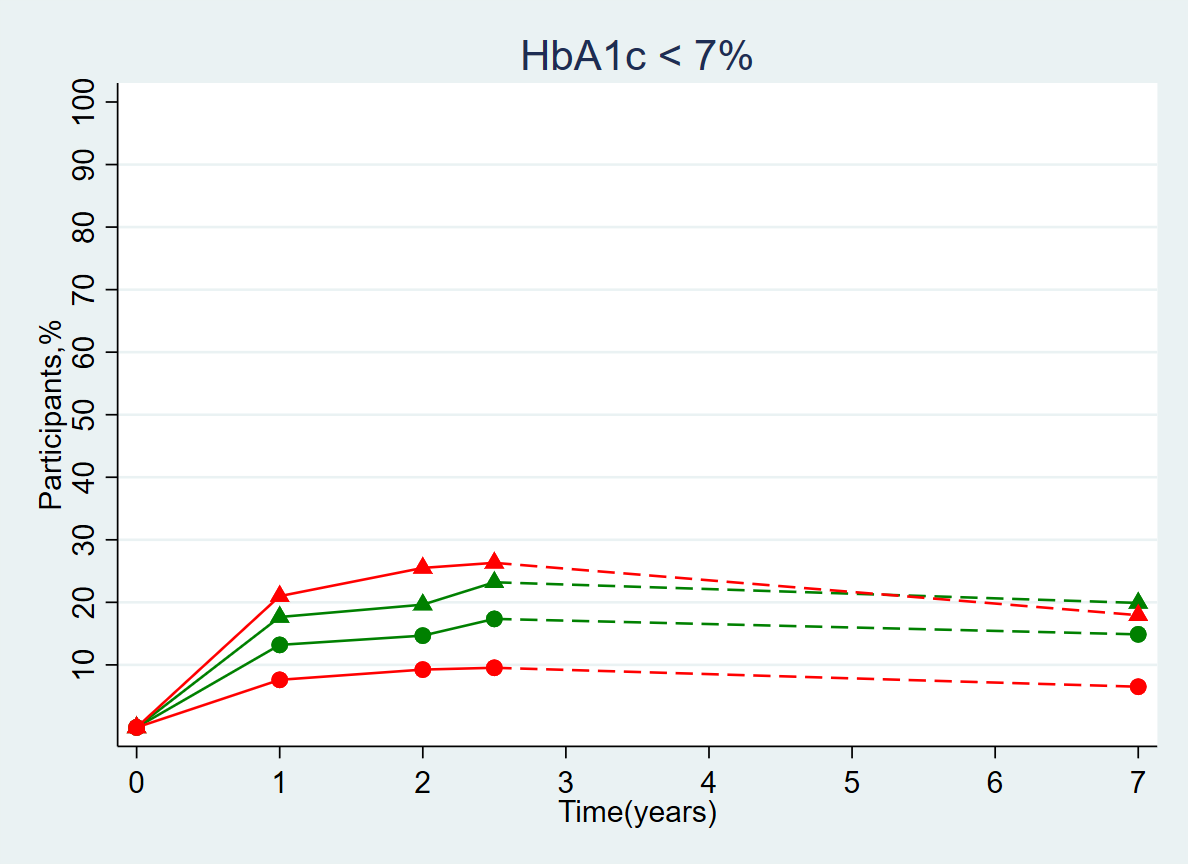

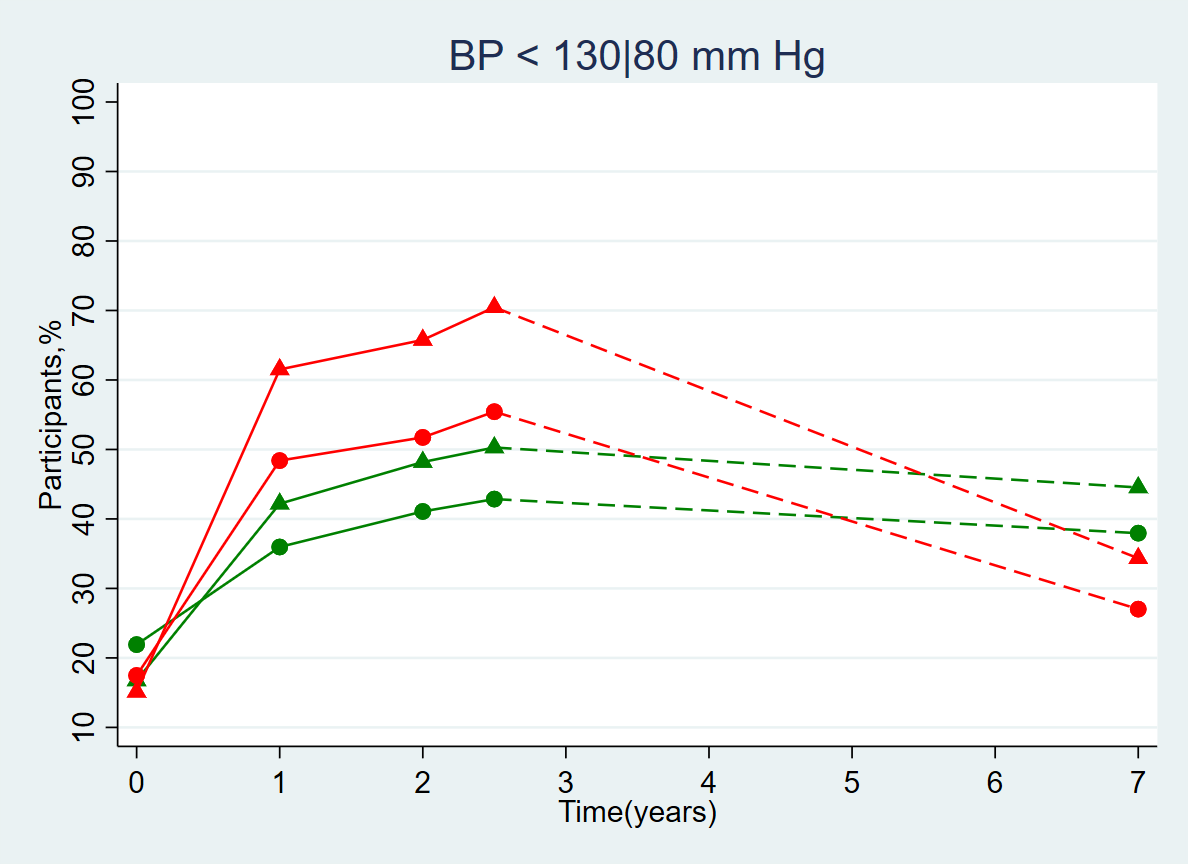

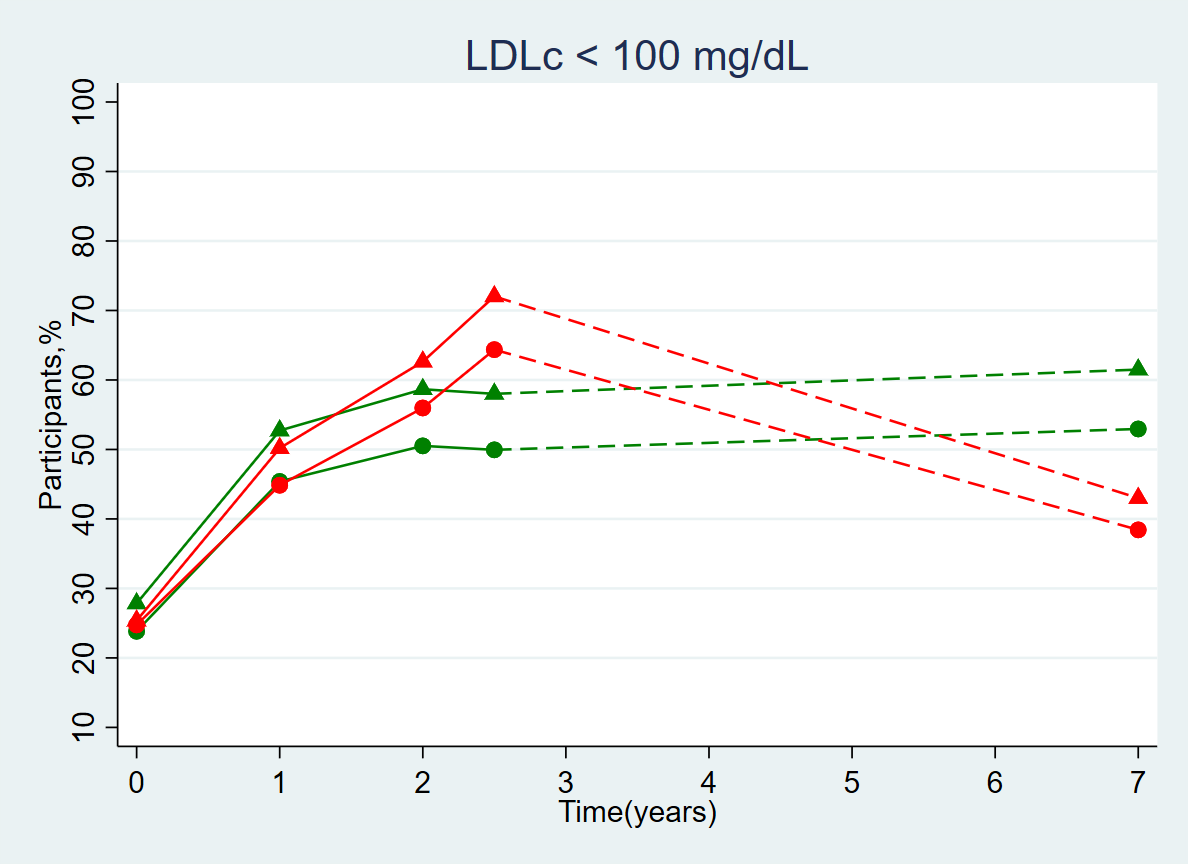


Δ Intervention • Usual Care

Green font: sites that continue intervention

Red font: sites that did not continue intervention

Data reported are percentages that obtained multiple or single risk factor control over the time period. Estimates combined all nonmissing values collected at baseline, year 1, 2, 3, 4, 5, 6, and 7 (end of the study). Model terms included treatment group, time, respective baseline value, age, sex and site.

Dashed lines show measures and trends between end-of-study (30 months) and end-of-follow-up (60 months)

BP, blood pressure; HbA1c, glycated hemoglobin; LDLc, low-density lipoprotein cholesterol

**Fig C.** Mean changes in HbA1c, SBP, DBP, and LDL-c at baseline, 2.5 years, and 6.5 years by treatment assignment and sites that continued versus discontinued active intervention


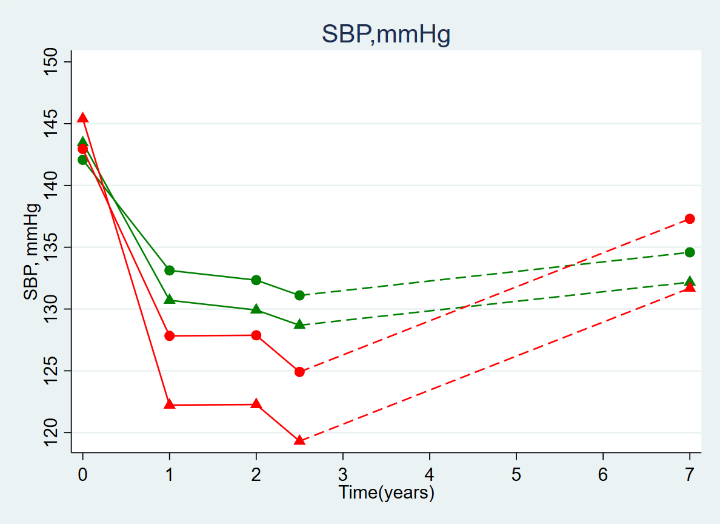

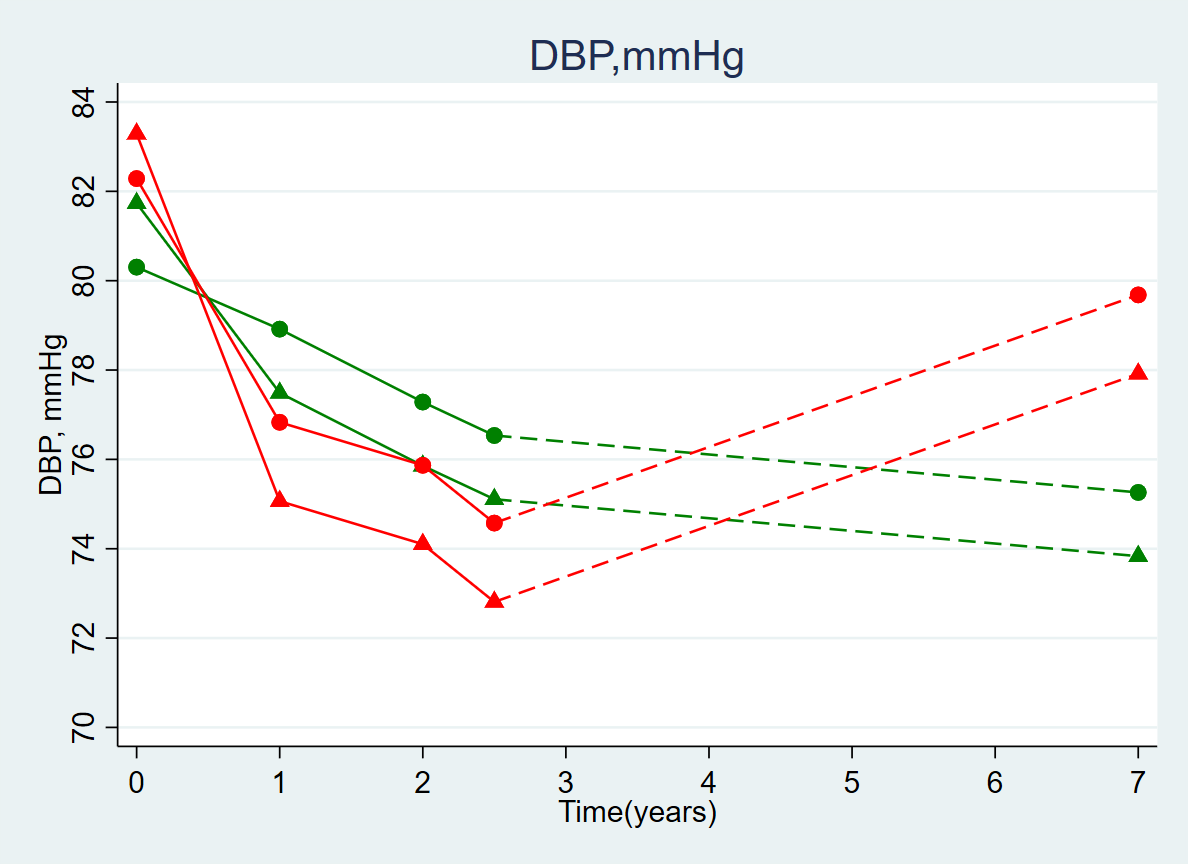

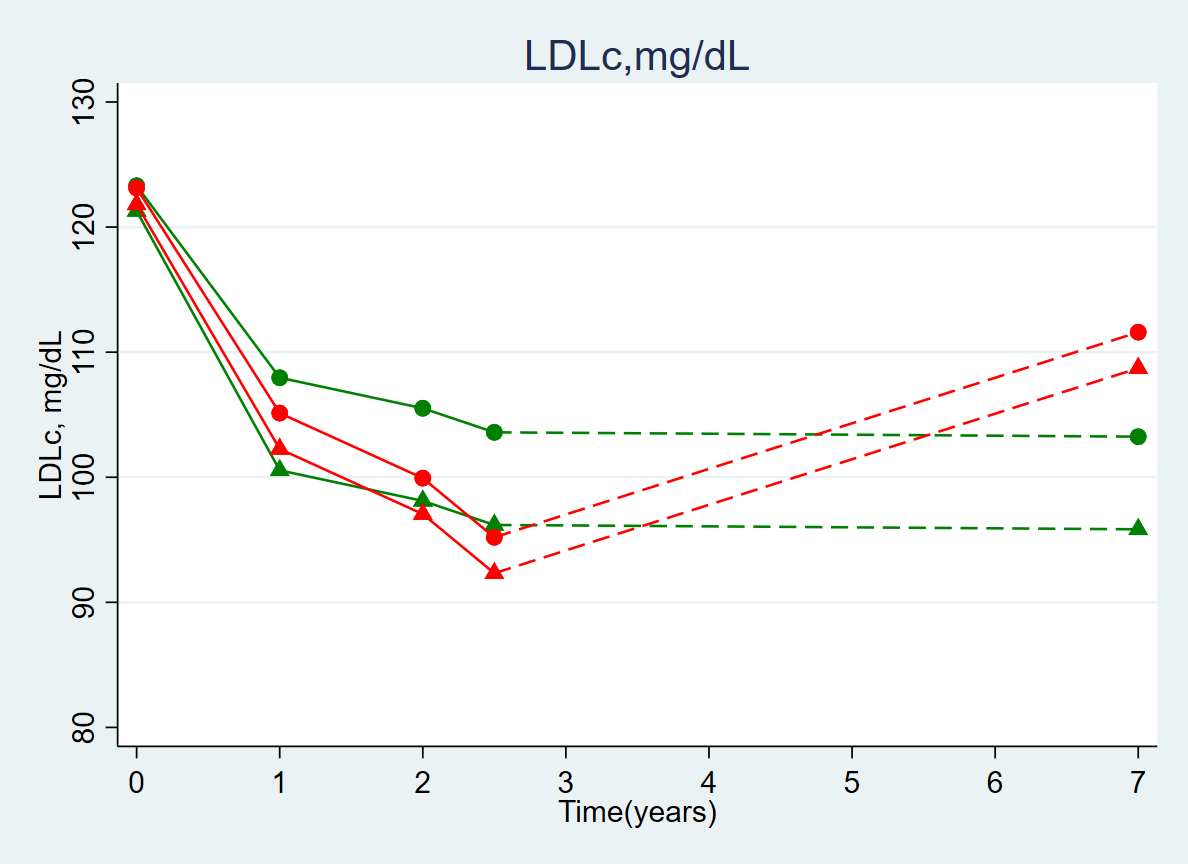

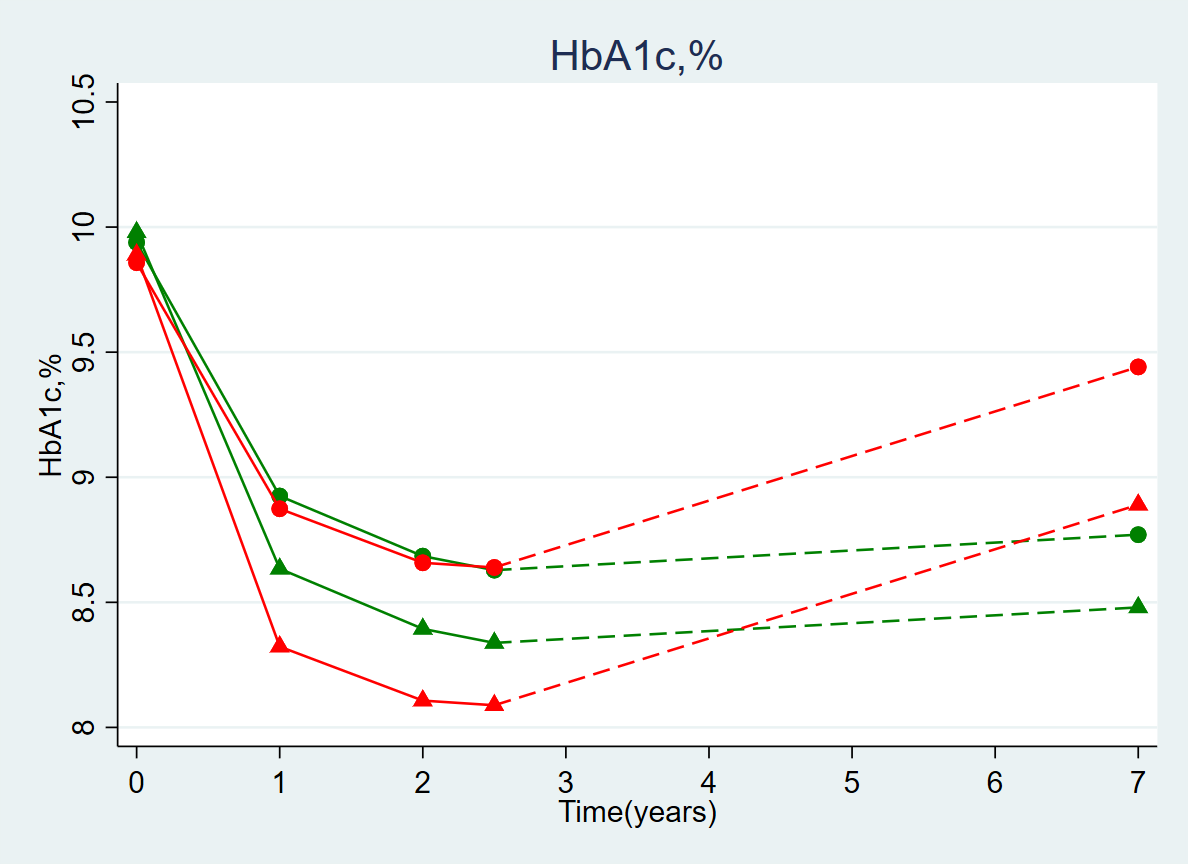


Δ Intervention • Usual Care

Green font: sites that continue intervention

Red font: sites that did not continue intervention

Data reported are mean values for hbA1c, SBP, LDLc and DBP over the time period. Estimates combine all nonmissing values collected at baseline, year 1, 2, 3, 4, 5, 6 and 7 (end of the study). Model terms included treatment group, time, respective baseline value, age, sex and site.

Dashed lines show measures and trends between end-of-study (30 months) and end-of-follow-up (60 months)

DBP, diastolic blood pressure; HbA1c, glycated hemoglobin; LDLc, low-density lipoprotein cholesterol; SBP, systolic blood pressure.

**Fig D**: Intervention Effects on Multiple Risk Factor Control by Baseline Socioeconomic and Clinical Characteristics


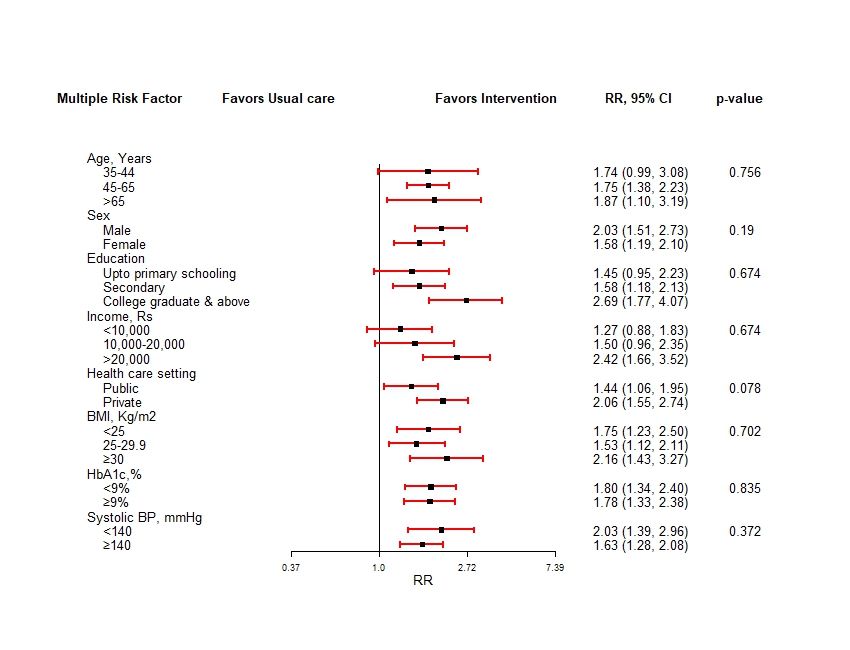


*x-axis is a logarithmic scale*

**Table B**. Relative Risks and Risk Differences for Composite and Individual Macrovascular and Microvascular endpoints

|  | Events, Number (%) | | |  |  | Event rate (ER) per 100 person years, ER (95% CI) | | | | HR, 95% CI | |  |
| --- | --- | --- | --- | --- | --- | --- | --- | --- | --- | --- | --- | --- |
|  | Overall n=1146 | Intervention group n=575 | Usual care group n=571 | Absolute risk difference (%) | NNT | Overall | Intervention group | Usual care group | Log rank test (p-value) | unadjusted | adjusted^1^ | |
| First Macrovascular Events | 105 (9.2%) | 48 (8.4%) | 57 (10.0%) | -1.63 (-4.97, 1.71) | 61 | 1.80  (1.48, 2.18) | 1.64  (1.24, 2.19) | 1.95  (1.51, 2.53) | 0.406 | 0.85  (0.58, 1.25) | 0.85  (0.58, 1.25) | |
| First Microvascular Events | 410 (35.8%) | 182 (31.7%) | 228 (39.9%) | -8.3 (-13.8, -0.03) | 12 | 8.17  (7.90, 9.59) | 7.54  (6.52, 8.72) | 9.94  (8.73, 11.3) | 0.0043 | 0.75  (0.62, 0.92) | 0.68  (0.56, 0.83) | |
| Composite of macrovascular and microvascular endpoints | 507 (44.2%) | 233 (40.5%) | 274 (48.0%) | -7.46 (-13.2, -1.73) | 13 | 11.05  (10.13, 12.05) | 9.88  (8.69, 11.23) | 12.28  (10.91, 13.83) | 0.012 | 0.80  (0.67, 0.95) | 0.72  (0.61, 0.86) | |
| **Single Macrovascular Events** |  |  |  |  |  |  |  |  |  |  |  | |
| *All deaths* | 96 (8.4%) | 51 (8.9%) | 45 (7.9%) | 0.99  (-2.22, 4.2) | 101 | 1.60  (1.31, 1.95) | 1.71  (1.3, 2.25) | 1.49  (1.11, 2) | 0.461 | 1.16  (0.78, 1.74) | 1.19  (0.80, 1.79) | |
| *Cardiovascular deaths* | 40 (3.5%) | 22 (3.8%) | 18 (3.2%) | 0.67  (-1.45, 2.8) | 148 | 0.67  (0.49, 0.91) | 0.74  (0.49, 1.12) | 0.60  (0.38, 0.95) | 0.483 | 1.25  (0.67, 2.33) | 1.34  (0.71, 2.5) | |
| *Non-fatal MI* | 38 (3.3%) | 18 (3.1%) | 20 (3.5%) | -0.37  (-2.45, 1.7) | 269 | 0.64  (0.47, 0.89) | 0.61  (0.39, 0.97) | 0.68  (0.44, 1.05) | 0.777 | 0.91  (0.48, 1.72) | 0.92  (0.49, 1.74) | |
| *Non-fatal Stroke* | 17 (1.4%) | 8 (1.4%) | 9 (1.6%) | -0.18  (-1.58, 1.22) | 541 | 0.29  (0.18, 0.46) | 0.27  (0.13, 0.54) | 0.30  (0.16, 0.58) | 0.822 | 0.90  (0.35, 2.32) | 0.96  (0.37, 2.51) | |
| *Amputation* | 9 (0.8%) | 6 (1.0%) | 3 (0.5%) | 0.52  (-0.5, 1.54) | 193 | 0.15  (0.08 ,0.29) | 0.2  (0.09 ,0.45) | 0.1  (0.03 ,0.31) | 0.309 | 2.02  (0.51, 8.1) | 2.22  (0.55, 8.9) | |
| *Revascularisation* | 26 (2.3%) | 11 (1.9%) | 15 (2.6%) | -0.71  (-2.44, 1.01) | 140 | 0.43  (0.29, 0.64) | 0.37  (0.21, 0.67) | 0.50  (0.30, 0.84) | 0.456 | 0.74  (0.34, 1.62) | 0.74  (0.34, 1.61) | |
| **Single Microvascular Events** |  |  |  |  |  |  |  |  |  |  |  | |
| *Retinopathy* | 69 (6.0%) | 30 (5.2%) | 39 (6.8%) | -1.61  (-4.37, 1.14) | 62 | 1.2  (0.95, 1.52) | 1.05  (0.73, 1.5) | 1.35  (0.99, 1.85) | 0.272 | 0.77  (0.48, 1.23) | 0.72  (0.45, 1.16) | |
| *Nephropathy* | 71 (6.2%) | 33 (5.7%) | 38 (6.7%) | -0.92  (-3.71, 1.88) | 109 | 1.2  (0.95, 1.51) | 1.12  (0.8, 1.57) | 1.28  (0.93, 1.76) | 0.600 | 0.88  (0.55, 1.41) | 0.87  (0.55, 1.4) | |
| *Macroalbuminuria* | 49 (4.3%) | 23 (4.0%) | 26 (4.6%) | -0.55  (-2.9, 1.79) | 181 | 0.82  (0.62, 1.08) | 0.78  (0.52, 1.17) | 0.87  (0.59, 1.27) | 0.727 | 0.90  (0.52, 1.59) | 0.90  (0.51, 1.59) | |
| *Doubling of creatinine* | 26 (2.3%) | 10 (1.7%) | 16 (2.8%) | -1.06  (-2.79, 0.66) | 94 | 0.43  (0.29, 0.64) | 0.33  (0.18, 0.62) | 0.53  (0.33, 0.87) | 0.248 | 0.63  (0.29, 1.39) | 0.63  (0.28, 1.38) | |
| *Renal Failure* | 7 (0.6%) | 1 (0.2%) | 6 (1.1%) | -0.88  (-1.78, 0.03) | 114 |  |  |  |  |  |  | |
| *Neuropathy* | 355 (31.0%) | 159 (27.7%) | 196 (34.3%) | -6.67  (-12.02, -1.33) | 15 | 7.2  (6.5, 8.0) | 6.4  (5.5, 7.4) | 8.1  (7.0, 9.3) | 0.021 | 0.78  (0.63, 0.96) | 0.71  (0.57, 0.87) | |

NNT, Number needed to treat; HR, hazard ratio

^1^ Adjusted for age, sex and site, p-value calculated using log rank test

Absolute risk difference calculated as difference of events in intervention minus usual care group; Number needed to treat calculated as 1 divided by absolute risk difference

First macrovascular events defined as death from cardiovascular causes or non-fatal myocardial infarction or stroke, or revascularization [angioplasty or coronary artery bypass graft])

First microvascular events defined as diabetic retinopathy, diabetic nephropathy, and diabetic neuropathy.

Composite of macrovascular and microvascular complications defined as all deaths, non-fatal myocardial infarction or stroke, revascularization, amputation, diabetic retinopathy, diabetic nephropathy, and diabetic neuropathy.

Nephropathy defined as macroalbuminuria or doubling of creatinine or renal failure/dialysis; macroalbuminuria is defined as a urinary albumin-to-creatinine (UACR) level of more than 300 mg/g Cr in any follow-up; doubling of creatinine calculated as 2x increase in Creatinine values of at least 200 μmol/L.

Blank cells implies insufficient power to provide credible estimates.

**Table C**. Relative Risks and Risk Differences for Composite and Individual Macrovascular and Microvascular endpoints – Sensitivity Analysis limited to sites that continued the intervention over 2.5 years to 6.5 years post-randomization

|  | Events, Number (%) | | |  |  | Event rate (ER) per 100 person years, ER (95% CI) | | | | HR, 95% CI | |  |
| --- | --- | --- | --- | --- | --- | --- | --- | --- | --- | --- | --- | --- |
|  | Overall n=735 | Intervention group n=370 | Usual care group n=365 | Absolute risk difference (%) | NNT | Overall | Intervention group | Usual care group | Log rank test (p-value) | unadjusted | adjusted^1^ | |
| First Macrovascular Event | 71 (9.7%) | 33 (8.9%) | 38 (10.4%) | -1.49  (-5.76, 2.78) | 67 | 1.85  (1.46 ,2.33) | 1.73  (1.23 ,2.43) | 1.96  (1.43 ,2.7) | 0.609 | 0.89  (0.56, 1.41) | 0.87  (0.54, 1.39) | |
| First Microvascular Event | 288 (39.1%) | 124 (33.5%) | 164 (44.9%) | -11.42  (-18.43, -4.41) | 9 | 9.43  (8.40, 10.6) | 7.88  (6.61, 9.40) | 11.1 (9.50, 12.9) | 0.0023 | 0.68  (0.55, 0.88) | 0.68  (0.54, 0.86) | |
| Composite of macrovascular and microvascular endpoints | 352 (47.9%) | 155 (41.9%) | 197 (54.0%) | -12.1  (-19.3, -4.9) | 8 | 11.95  (10.77 ,13.27) | 10.22  (8.73 ,11.96) | 13.8  (12 ,15.87) | 0.002 | 0.72  (0.58, 0.89) | 0.71  (0.58, 0.88) | |
| **Single Macrovascular Event** |  |  |  |  |  |  |  |  |  |  |  | |
| *All deaths* | 62 (8.4%) | 30 (8.1%) | 32 (8.8%) | -0.66  (-4.68, 3.36) | 152 | 1.56  (1.22 ,2.00) | 1.53  (1.07 ,2.19) | 1.59  (1.12 ,2.25) | 0.879 | 0.96  (0.58, 1.58) | 0.99  (0.6, 1.63) | |
| *CV death* | 21 (2.9%) | 12 (3.2%) | 9 (2.5%) | 0.78  (-1.63, 3.18) | 129 | 0.53  (0.34 ,0.81) | 0.61  (0.35 ,1.08) | 0.45  (0.23 ,0.86) | 0.459 | 1.38  (0.58, 3.28) | 1.42  (0.6, 3.38) | |
| *Non-fatal MI* | 35 (4.8%) | 17 (4.6%) | 18 (4.9%) | -0.34  (-3.42, 2.74) | 297 | 0.90  (0.64 ,1.25) | 0.88  (0.55 ,1.42) | 0.92  (0.58 ,1.45) | 0.925 | 0.97  (0.50, 1.88) | 0.96  (0.50, 1.87) | |
| *Non-fatal Stroke* | 12 (1.6%) | 6 (1.6%) | 6 (1.6%) | -0.02  (-1.85, 1.81) | 4502 | 0.30  (0.17 ,0.53) | 0.31  (0.14 ,0.68) | 0.30  (0.13 ,0.67) | 0.984 | 1.01  (0.33, 3.14) | 1.08  (0.35, 3.38) | |
| *Amputation* | 6 (0.8%) | 5 (1.4%) | 1 (0.3%) | 1.08  (-0.22, 2.37) | 93 | 0.15  (0.07 ,0.34) | 0.26  (0.11 ,0.62) | 0.05  (0.01 ,0.35) | 0.097 | 5.11  (0.6, 43.74) | 5.26  (0.61, 45.14) | |
| *Revascularisation* | 19 (2.6%) | 9 (2.4%) | 10 (2.7%) | -0.31  (-2.6, 1.99) | 325 | 0.48  (0.31 ,0.76) | 0.46  (0.24 ,0.89) | 0.50  (0.27 ,0.94) | 0.167 | 0.92  (0.37, 2.27) | 0.89  (0.36, 2.2) | |
| **Single Microvascular Event** |  |  |  |  |  |  |  |  |  |  |  | |
| *Retinopathy* | 6 (0.8%) | 17 (4.6%) | 26 (7.1%) | -2.53  (-5.92, 0.86) | 40 | 1.12  (0.83 ,1.52) | 0.89  (0.56 ,1.44) | 1.35  (0.92 ,1.98) | 0.167 | 0.65  (0.35, 1.2) | 0.62  (0.34, 1.15) | |
| *Nephropathy* | 62 (8.4%) | 28 (7.6%) | 34 (9.3%) | -1.75  (-5.77, 2.27) | 57 | 1.58  (1.23 ,2.03) | 1.44  (0.99 ,2.08) | 1.72  (1.23 ,2.4) | 0.536 | 0.85  (0.52, 1.41) | 0.83  (0.5, 1.37) | |
| *Macroalbuminuria* | 48 (6.5%) | 22 (5.9%) | 26 (7.1%) | -0.31  (-2.6, 1.99) | 325 | 1.21  (0.92 ,1.61) | 1.13  (0.74 ,1.71) | 1.30  (0.89 ,1.91) | 0.671 | 0.88  (0.5, 1.56) | 0.86  (0.49, 1.53) | |
| *Doubling of creatinine* | 18 (2.4%) | 6 (1.6%) | 12 (3.3%) | -1.67  (-3.90, 0.57) | 60 | 0.45  (0.28 ,0.72) | 0.30  (0.14 ,0.68) | 0.60  (0.34 ,1.05) | 0.159 | 0.50  (0.19, 1.34) | 0.51  (0.19, 1.38) | |
| *Renal Failure* | 7 (1.0%) | 1 (0.3%) | 6 (1.6%) | -1.37  (-2.78, 0.03) | 73 |  |  |  |  |  |  | |
| *Neuropathy* | 245 (33.3%) | 107 (28.9%) | 138 (37.8%) | -8.9  (-15.7, -2.1) | 11 | 7.59  (6.7 ,8.6) | 6.55  (5.42 ,7.91) | 8.66  (7.33 ,10.24) | 0.023 | 0.75  (0.58, 0.96) | 0.72  (0.56, 0.93) | |

NNT, Number needed to treat; HR, hazard ratio

^1^ Adjusted for age, sex and site, p-value calculated using log rank test

Absolute risk difference calculated as difference of events in intervention minus usual care group; Number needed to treat calculated as 1 divided by absolute risk difference

First macrovascular events defined as death from cardiovascular causes or non-fatal myocardial infarction or stroke, or revascularization [angioplasty or coronary artery bypass graft])

First microvascular events defined as diabetic retinopathy, diabetic nephropathy, and diabetic neuropathy.

Composite of macrovascular and microvascular complications defined as all deaths, non-fatal myocardial infarction or stroke, revascularization, amputation, diabetic retinopathy, diabetic nephropathy, and diabetic neuropathy.

Nephropathy defined as macroalbuminuria or doubling of creatinine or renal failure/dialysis; macroalbuminuria is defined as a urinary albumin-to-creatinine (UACR) level of more than 300 mg/g Cr in any follow-up; doubling of creatinine calculated as 2x increase in Creatinine values of at least 200 μmol/L.

Blank cells implies insufficient power to provide credible estimates.

**Table D**. Relative Risks and Risk Differences for Composite and Individual Macrovascular and Microvascular endpoints – Sensitivity Analysis limited to sites that discontinued the intervention at 2.5 years post-randomization

|  | Events, Number (%) | | |  |  | Event rate (ER) per 100 person years, ER (95% CI) | | | | HR, 95% CI | |
| --- | --- | --- | --- | --- | --- | --- | --- | --- | --- | --- | --- |
|  | Overall n=411 | Intervention group n=205 | Usual care group n=206 | Absolute risk difference (%) | NNT | Overall | Intervention group | Usual care group | Log rank test (p-value) | unadjusted | adjusted^1^ |
| First Macrovascular Events | 34 (8.3%) | 15 (7.3%) | 19 (9.2%) | -1.91  (-7.23, 3.42) | 52 | 1.71  (1.22 ,2.39) | 1.49  (0.90 ,2.48) | 1.93  (1.23 ,3.03) | 0.468 | 0.78  (0.4, 1.53) | 0.79  (0.39, 1.58) |
| First Microvascular Events | 122 (29.7%) | 58 (28.3%) | 64 (31.1%) | -2.78  (-11.6, 6.05) | 36 | 7.38  (6.18, 8.12) | 6.91  (5.34, 8.93) | 7.88 (6.16,10.1) | 0.465 | 0.88 (0.61, 1.25) | 0.73 (0.51, 1.04) |
| Composite of macrovascular and microvascular endpoints | 155 (37.7%) | 78 (38.0%) | 77 (37.4%) | -0.01  (-5.91, 5.88) | 6752 | 9.42  (8.05 ,11.03) | 9.27  (7.42 ,11.57) | 9.59  (7.67 ,11.99) | 0.861 | 0.97  (0.71, 1.33) | 0.76  (0.55, 1.05) |
| **Single Macrovascular Event** |  |  |  |  |  |  |  |  |  |  |  |
| *All deaths* | 34 (8.3%) | 21 (10.2%) | 13 (6.3%) | 3.9  (-1.4, 9.2) | 25 | 1.68  (1.2 ,2.35) | 2.06  (1.34 ,3.16) | 1.29  (0.75 ,2.23) | 0.184 | 1.59  (0.8, 3.18) | 1.77  (0.87, 3.6) |
| *CV death* | 19 (4.6%) | 10 (4.9%) | 9 (4.4%) | 0.51  (-3.55, 4.57) | 196 | 0.95  (0.6 ,1.48) | 0.99  (0.53 ,1.84) | 0.90  (0.47 ,1.73) | 0.825 | 1.11  (0.45, 2.72) | 1.23  (0.49, 3.08) |
| *Non-fatal MI* | 3 (0.7%) | 1 (0.5%) | 2 (1.0%) | -0.5  (-2.1, 1.2) | 207 | 0.15  (0.05 ,0.47) | 0.10  (0.01 ,0.71) | 0.20  (0.05 ,0.81) | 0.557 | 0.49  (0.04, 5.45) | 0.45  (0.04, 5.03) |
| *Non-fatal Stroke* | 5 (1.2%) | 2 (1.0%) | 3 (1.5%) | -0.48  (-2.6, 1.64) | 208 | 0.25  (0.1 ,0.6) | 0.20  (0.05 ,0.79) | 0.30  (0.10 ,0.94) | 0.648 | 0.66  (0.11, 3.96) | 0.65  (0.1, 4.09) |
| *Amputation* | 3 (0.7%) | 1 (0.5%) | 2 (1.0%) | -0.5  (-2.1, 1.2) | 207 | 0.15  (0.05 ,0.47) | 0.20  (0.05 ,0.81) | 0.10  (0.01 ,0.7) | 0.547 | 0.49  (0.04, 5.36) | 0.5  (0.04, 5.77) |
| *Revascularisation* | 7 (1.7%) | 2 (1.0%) | 5 (2.4%) | -1.45  (-3.95, 1.04) | 69 | 0.35  (0.17 ,0.74) | 0.51  (0.21 ,1.22) | 0.20  (0.05 ,0.8) | 0.256 | 0.40  (0.08, 2.06) | 0.42  (0.08, 2.31) |
| **Single Microvascular Event** |  |  |  |  |  |  |  |  |  |  |  |
| *Retinopathy* | 26 (6.3%) | 13 (6.3%) | 13 (6.3%) | 0.03  (-4.68, 4.74) | 3249 | 1.35  (0.92 ,1.99) | 1.36  (0.79 ,2.33) | 1.35  (0.78 ,2.32) | 0.977 | 1.01  (0.47, 2.18) | 0.96  (0.44, 2.07) |
| *Nephropathy* | 9 (2.2%) | 5 (2.4%) | 4 (1.9%) | 0.5  (-2.33, 3.33) | 201 | 0.45  (0.23 ,0.87) | 0.50  (0.21 ,1.2) | 0.40  (0.15 ,1.07) | 0.797 | 1.19  (0.32, 4.43) | 1.27  (0.33, 4.86) |
| *Macroalbuminuria* | 1 (0.2%) | 1 (0.5%) | 0 (0.0%) | 0.49  (-0.47, 1.44) | 205 |  |  |  |  |  |  |
| *Doubling of creatinine* | 8 (1.9%) | 4 (2.0%) | 4 (1.9%) | na |  | 0.4 (0.2 ,0.8) | 0.40  (0.15 ,1.06) | 0.40  (0.15 ,1.07) | 0.944 | 0.95  (0.24, 3.81) | 1.03  (0.25, 4.22) |
| *Renal Failure* |  | 0 (0.0%) | 0 (0.0%) | na |  |  |  |  |  |  |  |
| *Neuropathy* | 110 (26.8%) | 52 (25.4%) | 58 (28.2%) | -2.8  (-11.3, 5.8) | 36 | 6.54  (5.42 ,7.88) | 6.04  (4.61 ,7.93) | 7.05  (5.45 ,9.12) | 0.448 | 0.87  (0.6, 1.26) | 0.72  (0.49, 1.06) |

NNT, Number needed to treat; HR, hazard ratio

^1^ Adjusted for age, sex and site, p-value calculated using log rank test

Absolute risk difference calculated as difference of events in intervention minus usual care group; Number needed to treat calculated as 1 divided by absolute risk difference

First macrovascular events defined as death from cardiovascular causes or non-fatal myocardial infarction or stroke, or revascularization [angioplasty or coronary artery bypass graft])

First microvascular events defined as diabetic retinopathy, diabetic nephropathy, and diabetic neuropathy.

Composite of macrovascular and microvascular complications defined as all deaths, non-fatal myocardial infarction or stroke, revascularization, amputation, diabetic retinopathy, diabetic nephropathy, and diabetic neuropathy.

Nephropathy defined as macroalbuminuria or doubling of creatinine or renal failure/dialysis; macroalbuminuria is defined as a urinary albumin-to-creatinine (UACR) level of more than 300 mg/g Cr in any follow-up; doubling of creatinine calculated as 2x increase in Creatinine values of at least 200 μmol/L.

Blank cells implies insufficient power to provide credible estimates.

**Fig E**: Intervention Effects on Composite First Macrovascular Outcomes by Baseline Socioeconomic and Clinical Characteristics


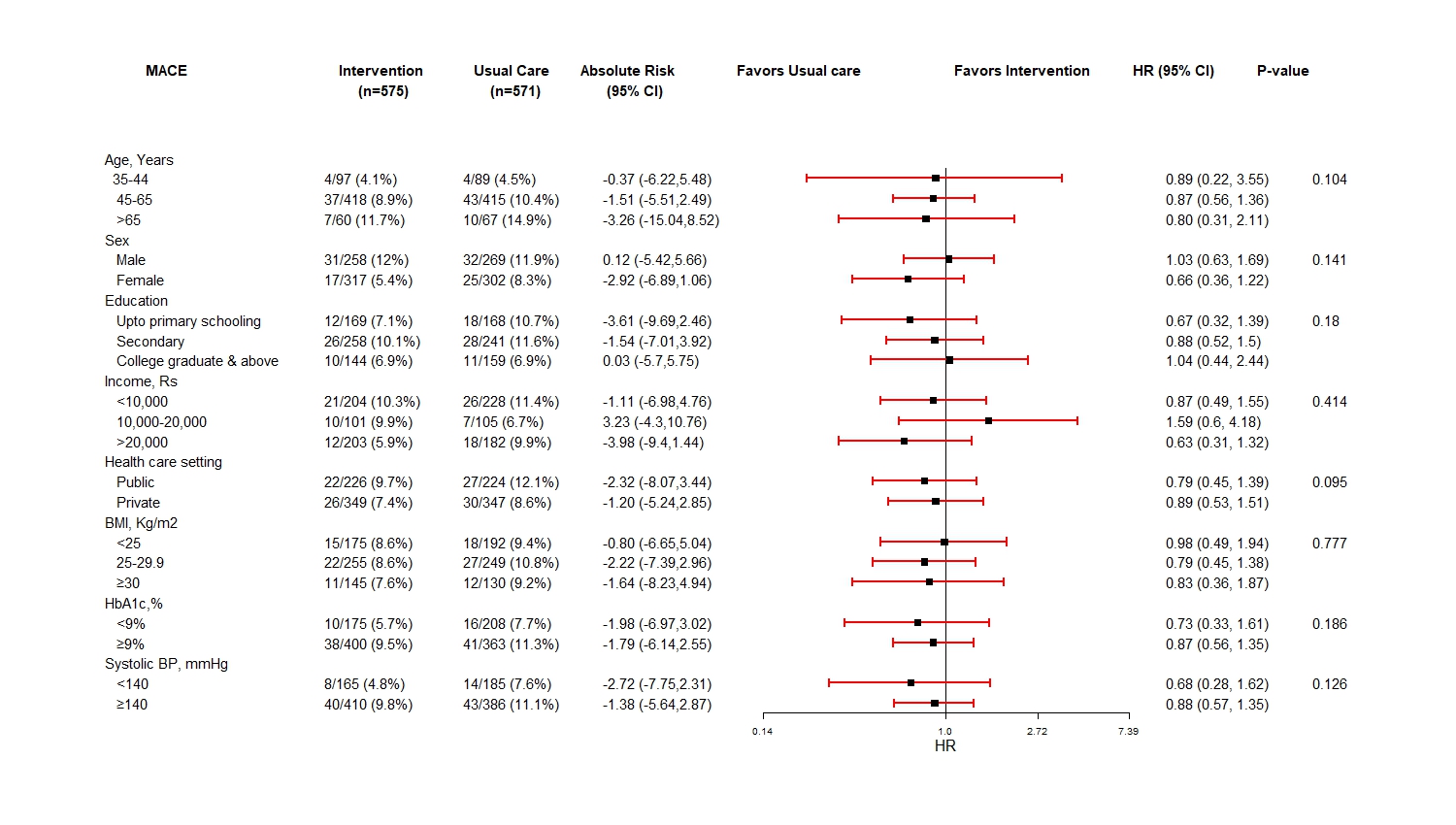


*Abbreviations: HR, hazards ratio; Rs, Indian Rupees; BMI, body mass index; HbA1c, glycated hemoglobin; LDLc, low-density lipoprotein cholesterol; BP, blood pressure*

*Hazards ratios estimated with a generalized estimating equation approach to account for correlation of observations within patients over time. Models adjusted for treatment group, time, treatment-by-time and treatment-by-subgroup interactions and site*

*p-values from treatment-by-subgroup interaction*

**Table E**: Serious Adverse Events by Treatment Assignment

|  | Intervention | Usual care | Overall |
| --- | --- | --- | --- |
| Arrythmia | 2 | 1 | 3 |
| Gastro Intestinal bleeding | 3 | 1 | 4 |
| Infection | 45 | 27 | 72 |
| Other surgical procedures (excluding CVD revascularisation) | 115 | 100 | 187 |
| Total Hospitalization | 141 | 113 | 254 |
| Acute glycemia-related hospitalizations* | 13 | 5 | 18 |
| Total | 319 | 247 | 566 |

*includes hospitalization due to Severe hypoglycemia and acute hyperglycemia (e.g. Diabetic Keto Acidosis

**Table F**: Baseline demographic and clinical characteristics of those who did versus did not complete at least one assessment visit

| Characteristic | Baseline | |  |
| --- | --- | --- | --- |
|  | ≥1 follow-up visit | No follow-up visits | p-value |
| Participants, n | n=1082 | n=64 |  |
| Age, mean (SD) | 54.2 (9.2) | 52.8 (9.5) | 0.24 |
| Sex, % |  |  |  |
| *Male* | 498 (46.0%) | 29 (45.3%) | 0.91 |
| *Female* | 584 (54.0%) | 35 (54.7%) |  |
| Education, % |  |  |  |
| *Upto primary schooling* | 311 (28.7%) | 26 (40.6%) | 0.09 |
| *Secondary* | 476 (44.0%) | 23 (35.9%) |  |
| *College graduate & above* | 290 (26.8%) | 13 (20.3%) |  |
| *missing* | 5 (0.5%) | 2 (3.1%) |  |
| Household income, % |  |  |  |
| *<10,000* | 406 (37.5%) | 26 (40.6%) | 0.38 |
| *10,000-20,000* | 199 (18.4%) | 7 (10.9%) |  |
| *>20,000* | 365 (33.7%) | 20 (31.3%) |  |
| *unknown* | 112 (10.4%) | 11 (17.2%) |  |
| Current smoker, % | 33 (3.0%) | 1 (1.6%) | 0.50 |
| Comorbidities, % |  |  |  |
| Previous cardiovascular disease, % | 73 (6.7%) | 5 (7.8%) | 0.74 |
| *Myocardial infarction* | 32 (3.0%) | 3 (4.7%) | 0.43 |
| *Coronary heart disease* | 70 (6.5%) | 5 (7.8%) | 0.67 |
| *Stroke* | 21 (1.9%) | 2 (3.1%) | 0.51 |
| Waist circumference, cm | 96.0 (11.4) | 97.8 (11.0) | 0.23 |
| Weight, kg | 69.1 (12.7) | 70.6 (16.5) | 0.36 |
| Body mass index, Kg/m^2^ | 27.4 (5.0) | 28.1 (5.0) | 0.34 |
| Metabolic variables |  |  |  |
| Mean hemoglobin A1c level (SD), % | 9.9 (1.6) | 10.2 (1.5) | 0.21 |
| Mean Fasting Blood glucose(SD) |  |  |  |
| *mmol/L* | 9.81 (3.52) | 10.71 (4.11) |  |
| *mg/dL* | 176.7 (63.4) | 193.0 (74.0) | 0.05 |
| Mean low-density lipoprotein cholesterol level (SD) |  |  |  |
| *mmol/L* | 3.16 (0.96) | 3.23 (0.95) |  |
| *mg/dL* | 122.2 (36.9) | 124.9 (36.8) | 0.57 |
| Mean high-density lipoprotein cholesterol level (SD) |  |  |  |
| *mmol/L* | 1.14 (2.31) | 1.14 (0.23) |  |
| *mg/dL* | 44.0 (8.9) | 44.2 (8.8) | 0.85 |
| Median triglyceride level (IQR) |  |  |  |
| *mmol/L* | 1.57 (1.19, 2.16) | 1.84 (1.30, 2.38) |  |
| *mg/dL* | 139.0 (105.0, 191.0) | 163.0 (115.0, 211.0) | 0.09 |
| Mean total cholesterol level (SD) |  |  |  |
| *mmol/L* | 5.05 (1.15) | 5.15 (1.27) |  |
| *mg/dL* | 195.0 (44.5) | 198.7 (49.0) | 0.52 |
| Mean systolic blood pressure (SD), mm Hg | 143.1 (19.5) | 146.0 (17.3) | 0.26 |
| Mean diastolic blood pressure (SD), mm Hg | 81.5 (10.9) | 84.9 (9.6) | 0.01 |

**Table G**: Baseline demographic and clinical characteristics of patients at sites that continued versus discontinued the intervention

| Characteristic | Baseline | |  |
| --- | --- | --- | --- |
|  | Continued Intervention | Discontinued Intervention | p value |
| Participants, n | n=735 | n=411 |  |
| Age, mean (SD) | 54.5 (9.1) | 53.5 (9.3) | 0.09 |
| Sex, % |  |  |  |
| *Male* | 341 (46.4%) | 186 (45.3%) | 0.71 |
| *Female* | 394 (53.6%) | 225 (54.7%) |  |
| Education, % |  |  |  |
| *Upto primary schooling* | 200 (27.2%) | 137 (33.3%) | 0.02 |
| *Secondary* | 342 (46.5%) | 157 (38.2%) |  |
| *College graduate & above* | 187 (25.4%) | 116 (28.2%) |  |
| *missing* | 6 (0.8%) | 1 (0.2%) |  |
| Household income, % |  |  |  |
| *<10,000* | 274 (37.3%) | 158 (38.4%) | 0.002 |
| *10,000-20,000* | 152 (20.7%) | 54 (13.1%) |  |
| *>20,000* | 229 (31.2%) | 156 (38.0%) |  |
| *unknown* | 80 (10.9%) | 43 (10.5%) |  |
| Current smoker, % | 27 (3.7%) | 7 (1.7%) | 0.06 |
| Comorbidities, % |  |  |  |
| Previous cardiovascular disease, % | 51 (6.9%) | 27 (6.6%) | 0.81 |
| *Myocardial infarction* | 22 (3.0%) | 13 (3.2%) | 0.87 |
| *Coronary heart disease* | 48 (6.5%) | 27 (6.6%) | 0.98 |
| *Stroke* | 11 (1.5%) | 12 (2.9%) | 0.10 |
| Waist circumference, cm | 97.0 (11.9) | 94.5 (10.2) | <0.001 |
| Weight, kg | 68.8 (12.9) | 69.7 (13.1) | 0.28 |
| Body mass index, Kg/m^2^ | 27.3 (5.1) | 27.7 (4.9) | 0.30 |
| Metabolic variables |  |  |  |
| Mean hemoglobin A1c level (SD), % | 10.0 (1.6) | 9.9 (1.6) | 0.39 |
| Mean Fasting Blood glucose(SD) |  |  |  |
| *mmol/L* | 10.0 (3.57) | 9.61 (3.53) |  |
| *mg/dL* | 180.2 (64.3) | 173.1 (63.6) | 0.07 |
| Mean low-density lipoprotein cholesterol level (SD) |  |  |  |
| *mmol/L* | 3.17 (0.95) | 3.17 (0.96) |  |
| *mg/dL* | 122.3 (36.8) | 122.5 (37.1) | 0.94 |
| Mean high-density lipoprotein cholesterol level (SD) |  |  |  |
| *mmol/L* | 1.17 (0.25) | 1.09 (0.17) |  |
| *mg/dL* | 45.1 (9.8) | 42.1 (6.6) | <0.001 |
| Median triglyceride level (IQR) |  |  |  |
| *mmol/L* | 1.55 (1.16, 2.14) | 1.60 (1.22, 2.27) |  |
| *mg/dL* | 137.0 (103.0, 189.0) | 142.0 (108.0, 201.0) | 0.04 |
| Mean total cholesterol level (SD) |  |  |  |
| *mmol/L* | 5.06 (1.20) | 5.06 (1.09) |  |
| *mg/dL* | 195.2 (46.2) | 195.2 (42.2) | 1.00 |
| Mean systolic blood pressure (SD), mm Hg | 142.8 (19.6) | 144.2 (19.2) | 0.24 |
| Mean diastolic blood pressure (SD), mm Hg | 81.0 (11.0) | 82.8 (10.6) | 0.01 |

**Table H**: Sensitivity Analysis examining Multiple and Single Risk Factor Control using Alternative Statistical Approaches

|  | Relative risk (95% CI) | | | |
| --- | --- | --- | --- | --- |
|  | GEE* | GEE^#^ | IPW* | IPW^#^ |
| Multiple risk factor control | 1.76 (1.43, 2.16) | 1.79 (1.45, 2.2) | 1.34 (1.12, 1.61) | 1.35 (1.12, 1.62) |
| HbA1c <7% | 1.58 (1.32, 1.91) | 1.60 (1.32, 1.94) | 1.20 (1.02, 1.40) | 1.21 (1.03, 1.41) |
| BP <130/80  mm Hg | 1.21 (1.11, 1.31) | 1.20 (1.11, 1.31) | 1.10 (0.99, 1.22) | 1.10 (0.99, 1.21) |
| LDLc <100 mg/dL (<70 with history of CVD) | 1.16 (1.08, 1.24) | 1.16 (1.08, 1.24) | 1.18 (1.07, 1.31) | 1.19 (1.08, 1.31) |

Multiple risk factor control defined as HbA1c <8% & either BP <130/80 mmHg and/or LDLc <100 mg/dL [<70 mg/dL with history of CVD]

* Models adjusted for site and respective baseline values

# Models adjusted for age, sex, site and respective baseline values

Relative risk calculated using generalised estimating equation via log-binomial regression or Poisson regression

# **Table I**: Sensitivity Analysis examining Multiple Risk Factor Control using Controlled Imputation to Account for Non-random Missing Data

| Table: Sensitivity analysis results |  |  |  |
| --- | --- | --- | --- |
|  | **Treatment effect** | **Standard error** | **p value** |
| Primary analysis^1^ | 1.77 (1.45, 2.17) | 0.1824 | <0.001 |
| Primary analysis^2^ | 1.77 (1.45, 2.17) | 0.1824 | <0.00 |
|  |  |  |  |
| Sensitivity analysis |  |  |  |
| Jump to Usual care^1^ | 1.83 (1.48, 2.26) | 0.1067 | <0.001 |
| Jump to Usual care^2^ | 1.83 (1.48, 2.25) | 0.1064 | <0.001 |
| ^1^covariates included in the model treatment group (Intervention/Usual care), time, and site (fixed effect) | | | |
| ^2^ covariates included in the model treatment group (Intervention/Usual care), time, age (continuous), sex (Male/Female) and site (fixed effect) | | | |

# **Table J**: Sensitivity Analysis examining Multiple and Single Risk Factor Control Adjusting for Clustering by Site Care Coordinator

|  | **GEE*** | **GEE^#^** | **Site co-ordinators as cluster^** |
| --- | --- | --- | --- |
| Multiple risk factor control | 1.76 (1.43, 2.16) | 1.79 (1.45, 2.20) | 1.78 (1.47, 2.15) |
| HbA1c <7% | 1.58 (1.32, 1.91) | 1.60 (1.32, 1.94) | 1.58 (1.34, 1.86) |
| BP <130/80  mm Hg | 1.21 (1.11, 1.31) | 1.20 (1.11, 1.31) | 1.17 (1.09, 1.26) |
| LDLc <100 mg/dL (<70 mg/dL with a history of CVD) | 1.16 (1.08, 1.24) | 1.16 (1.08, 1.24) | 1.16 (1.09, 1.24) |

Multiple risk factor control defined as HbA1c <8% & either BP <130/80 mmHg and/or LDLc <100 mg/dL [<70 mg/dL with history of CVD]

* Models adjusted for site and respective baseline values

# Models adjusted for age, sex, site and respective baseline values

^ with patients clustered by care coordinators and sites (Models adjusted for age, sex, site and respective baseline values)
