# Supplementary material for: Effect of a multicomponent quality improvement strategy on sustained achievement of diabetes care goals and macrovascular and microvascular complications in South Asia at 6.5 years follow-up: Post hoc analyses of the CARRS randomized clinical trial
Source: PLoS Med. 2024 Jun 3;21(6):e1004335. doi: 10.1371/journal.pmed.1004335 (PMC11198027; doi:10.1371/journal.pmed.1004335)
Supplement: Attachment — Submitted filename: AuthorRESPONSES_2024-03-19.docx [file pmed.1004335.s005.docx]

*PLoS Medicine* Manuscript PMEDICINE-D-23-03727R1

“Effect of a Multicomponent Quality Improvement Strategy on Sustained Achievement of Diabetes Care Goals and Macrovascular and Microvascular Complications in South Asia at 6.5 years: the CARRS Randomized Clinical Trial"

**Comments and Author Responses**

The authors greatly appreciate the Editors’ and Reviewers’ efforts in reviewing our revised manuscript critically and for this opportunity to respond to the queries and comments. We believe these edits have improved our manuscript substantially.

We have addressed the comments below and have attached the tracked change and clean version of the manuscript for your perusal. References to locations where revisions can be found are referring to the document with tracked changes.

**Editorial Comments**

1) We think that your manuscript has the potential to offer a valuable contribution, but we are concerned about the vast number of discrepancies between the study protocol and the manuscript. Accurate study reporting is vitally important and currently there is a significant lack of clarity regarding a number of points.

Thank you for the careful review of the manuscript and the suggestions below. We have revised the manuscript using these critiques and hope it is even clearer and a good addition to the literature.

2) Due to the discrepancies between the protocol and the manuscript we found it rather difficult to discern whether this study is an entirely post-hoc analysis/follow-up study or whether it is pre-specified as part of the primary trial. This point requires definitive clarification throughout, including in the title and the abstract as well as the main methods section of your manuscript.
Thank you for the opportunity to clarify this. We appreciate the importance of this and detail here the events that led to this discordance, as well as our recommendation for how to address this:

a. The original trial was funded and powered to evaluate between-group differences in multiple risk factor control at ten clinic sites (n=1,120), as detailed in Protocol version 2.6 dated May 2011. We recruited and randomized 1,146. This component was completed and the findings were published in the *Annals of Internal Medicine* 2016 (<https://doi.org/10.7326/M15-2807>)
b. We subsequently sought funds for continuation of the trial to establish whether there were differences in microvascular and macrovascular complications of diabetes. When we secured modest funds for trial continuation, we amended the protocol (Protocol version 3.0 dated Sept 2016) to focus on a co-primary outcome of:
   i. Major adverse cardiovascular events (MACE), for which power calculations are shown in section 9.2 of Protocol version 3 (Sept 2016)
   ii. Sustained risk factor control differences, based on the prior power calculation that was in Protocol version 1
c. However, as we sought to implement the new protocol, four sites decided (due to administrative and financial reasons) to discontinue implementation of the intervention. These same sites permitted access to assess risk factors and events in their participants, as originally randomized. At that time, the study team did not update the protocol and this caused the discrepancies noted by Editors and Reviewers.
d. We considered the path forward. Due to the discrepancy in detailing the co-primary outcomes and because we likely had lower power at the six remaining sites than was needed to evaluate sustained risk factor control at ten sites, the only sensible path will be to describe the report as a post-hoc analysis where we treated sustained risk factor control and MACE as post hoc co-primary outcomes. We have made these adjustments in the **Title**, the **Abstract**, **Methods**, and **Discussion**.
e. We welcome additional suggestions from the Editors and Reviewers

Either way this study should be reported in line with CONSORT and the CONSORT checklist included as supporting information when you re-submit your manuscript. If this is a post-hoc analysis/follow-up study we suggest reporting in line with CONSORT explicitly stating this is a follow-on study and ensuring that the abstract details the main trial items in 2-3 sentences, including the study population, dates, intervention and primary outcome. The majority of the abstract should then describe the complete details of this study.
As recommended, we have included the CONSORT checklist in the document that includes Supplementary Appendices (**Supplement 1**). Apologies that this was not uploaded on the original submission.

3) You state that randomization began in 2011 but the protocol included here is dated 2016. Please include the original protocol for the primary trial that was submitted as part of the trial approval process and please clarify/justify any amendments between the original protocol and the 2016 version included here.
We appreciate the opportunity to clarify this and have included both the 2011 and 2016 protocols in the submission (please see **Supplement 1** documents). In addition, in response to query #2 above, we have detailed why the differences exist.

**Comments from Reviewers**

**Reviewer #1**: The manuscript 'Effect of a Multicomponent Quality Improvement Strategy on Sustained Achievement of Diabetes Care…' is a welcome addition to the initial results of the 2.5 year follow up that were published.

Not only has the work been meticulously conceived and performed, careful attention has been given to identifying and clarifying some of the doubts that may arise: eg any difference(s) in the subjects from centers which dropped out from the second leg (there were none), reason for the centers dropping out ('administrative reasons'), handling missing data ('sensitivity analysis of outcomes using inverse probability weighting')

Thank you for the careful review of the manuscript and the suggestions below. We have revised the manuscript using these critiques and hope it is even clearer and a good addition to the literature.

Specific comments:
(1)   The outcomes of patients from centers which dropped out in the second phase were assessed by telephonic interview, which is soft evidence

Thank you for this comment. Unfortunately, for some participants that moved from the original clinic location or declined to come in for a visit, we optimized retention by phoning them for information about events and mortality, and complemented this by confirming their health status with clinic records of those participants’ medical history. We have noted that this was a limitation **(*Page 14, lines 504-507*)**.

(2)   Were any other measures studied other than the primary end points? The authors mention 'behaviour characteristics' in their results. What were they? In case they were assessed separately, were there any differences? Because hard endpoints (macrovascular and microvascular disease) are difficult to modify with the kind of interventions that were possible in the study. If any factors such as stress, distress and depressive symptoms were studied, they could have been improved with the current interventions without improvements of vascular complications. They may cite results of other work in which some of the authors were involved (INDEPENDENT study)

We appreciate the opportunity to clarify this. We did not report changes in behaviors or psychosocial health in this report which was focused on macrovascular and microvascular diseases. We have noted, in the discussion, that it is possible that changes in behaviors and psychological health may have played a role; we have also cited the INDEPENDENT study, as recommended **(*Page 14, lines 483-486*)**.

(3)   The authors may mention the diverse health care centers where the interventions occurred (public and private settings) and the soft end-points as the basis for diagnosis.

Thank you - we have made this clearer **(*Page 6, line 203*)**.

(4)   Despite diverse source of fundings, careful oversight and employment of dedicated care coordinators, the improvement in metabolic parameters was modest. Therefore, the authors may comment that attention should also be paid to primordial or primary preventive measures (maternal nutrition during pregnancy, dietary regulation, sleep, physical exercise, built environment and approaches to alleviating stress)

We appreciate this insightful point and indeed, we note that the effect on metabolic parameters was marked in the first phase of the study and wanes with time, unless the intervention is continued intensively or patients make substantial changes to their lifestyles. We have highlighted this further in the discussion **(*Page 14, lines 484-488*)**.

(5)   What is meant by 'IPW"?

To clarify, IPW stands for inverse probability weighting, which is a method of addressing missing data and serves as a robustness check. We have detailed this in the methods🡪 statistical analysis section **(*Page 11, line 373*)**

(6)   Minor issues:
a.    Reference 18 is written as a superscript; consistency in citation is required
b.    ‘6 sites..’; may be written as a word (‘six’)
c.    Replace ‘to’ with ‘and’ {At 2.5 years (replace ‘to’ with ‘and’) annually…
Thank you for these specific points – we have addressed each as recommended.

**Reviewer #2**: Thank you for the opportunity to review this paper. Although several RCT on multifactorial care in people with type 2 diabetes have been published and showed benefits on metabolic target attainment and clinical outcome, the majority were conducted in high-income areas. A key strength of the present study is the demonstration of feasibility and effects of a QI strategy in a low-resource area, and support recommendation to apply similar strategies in India. I commend the authors for undertaking this multi-center study which must have been a mammoth task!
We appreciate the positive sentiments, attentive review, and recommendations to improve the manuscript.

My main concern about this paper is that there appears to be a number of important discrepancies between the protocol (attached in the supplementary) and what are stated in the paper, with respect to definition of primary and secondary endpoints, planned vs actual intervention duration, and analysis approach (intention to treat or per protocol or both).

Thank you for this comment. Please see responses to the Editors’ comments above where we outline the discrepancies and how we have re-aligned:

1. We have clarified that this is a post hoc analysis as the changes in sites continuing versus discontinuing resulted in lower-than-expected power to assess cardiometabolic risk factor changes and vascular endpoints
2. We uploaded both the first and last protocol versions to our resubmission
3. We clarified, throughout the manuscript, the methods we use to test robustness of our estimates

-       Abstract – Please state which is the primary endpoint and which are the secondary endpoints in the abstract
-       Abstract – “Participants at sites that continued versus discontinued were no different” this sentence seems incomplete, no different in what?

Thank you for the opportunity to clarify these points. We have revised the wording in the abstract methods and results **(*Page 4, lines 139-144*)**.

-       Within each participating clinic, some patients would be assigned QI care and others usual care, but would they be seen by the same set or different set of physicians? If they were seen by the same physicians, how likely were they to provide different level of care to their patients of different treatment arm? It is mentioned in the methods that a web-based DS-EHR was available with care prompts to guide clinical decision for patients in the QI arm. Overtime, physicians became better trained in best clinical practice and it is highly likely that they would gradually apply similar level of care to their patients in usual care arm, potentially causing under-estimation of between-group differences in primary endpoint.

The reviewer has correctly interpreted this. We note in the methods and discussion that control arm participants may have benefited from physicians improving care delivery and as such, our estimates are likely conservative. We have further expounded on this in the methods **(*Page 7, lines 229-230*)** and discussion **(*Page 15, line 517-521*)**.

-       Please specify clearly the primary and secondary outcomes of the study in the text. From the protocol in the supplementary, Section 1.1 "primary objective" and Section 3 "study design" indicated that the primary outcome was between-group difference in proportions achieving multiple risk factor control targets. However, Section 9.1 states a co-primary outcome of "achieving multiple risk factor control targets and reduction in cumulative incidence of MACE", and in Section 9.2 "sample size estimation", the power calculation was based on incident MACE over 6.5 years follow-up. So, is "reduction in cumulative incidence of MACE" a primary outcome, a secondary outcome or a post-hoc clinical outcome? The description in methods and the analysis approach in the paper should align with that stated in the protocol.

Thank you for the opportunity to clarify this. As described above in response #2 to the Editor’s comments, we outline the discrepancies and how we have revised the manuscript:

1. We have clarified that all outcomes are post hoc analyses due to the changes in some sites continuing versus discontinuing resulting in lower-than-expected power to assess either cardiometabolic risk factor changes and vascular endpoints
2. We uploaded both the first and last protocol versions to our resubmission
3. We clarified, throughout the manuscript, the methods we use to test the robustness of our estimates

-       Also, how is the definition of MACE differed from "macrovascular complication"? I cannot find this information in the protocol.

Thank you for the opportunity to clarify – MACE and macrovascular complications was considered the same. We have made this clearer by removing the reference to MACE and instead using macrovascular endpoints throughout the manuscript.

-       What is the planned intervention period for the study? Is it 2.5 years or 6.5 years? From the protocol, under Section 3 "study design", it states that the mean follow-up period is 6.5 years, in Section 6.1 "schedule of evaluations", the right-most column read "2.5 year follow up for primary outcome", in Section 6.2.4 "3-monthly and intermediate visits", participants in intervention arm would have 3-monthly visit up to 39 months post randomisation, and in section 9.2 "sample size estimation", the study was powered to detect between group difference in cumulative incidence of MACE over 6.5 years. So, at the time of planning the study including sample size calculation, was the intention to intervene patients for 2.5 years and then follow-up without further intervention for another 4 years until 6.5 years, or to intervene patients for 6.5 years? I feel that this is important to clarify upfront to justify your statistical approach where you combined the results of participants from sites that continued active intervention and those from sites that discontinued in their analysis, as the period of intervention differed between these two groups of participants. Let's say the investigators have planned to continue intervention for 6.5 years at the outset (at least this is my impression based on reading the protocol), then based on intention to treat principal, one should analyse the data of all randomised participants including those that discontinued intervention at 2.5 years. However, in the per protocol analysis, those who discontinued at 2.5 years should probably not be included. All of these need to be clarified in the paper and present accordingly.

Thank you for the opportunity to clarify this. As described above in response #2 to the Editor’s comments, the original trial that was powered to evaluate between-group achievement of multiple risk factor control was designed to be 2.5 years in duration. We were able to secure funding to continue the intervention and monitoring of patients up until 6.5 years and had discontinuation of participation of some sites for this latter four year period. If participants had decided, of their own accord to continue or discontinue the intervention, then the reviewer’s point about ITT vs per protocol would be appropriate. In our instance, the decision to remain part of the study (and allow participants to continue in the arm they were originally randomized to) was not left to the discretion of the participants, but at the clinic leadership level. As a result, we have 3 groups: 1/ those never exposed to the intervention (control group throughout); 2/ those exposed to the intervention for 2.5 years and then not for years 2.5-6.5years (intervention participants at 4 sites that discontinued); and 3/ those exposed to the intervention for all 6.5 years (intervention participants at 6 sites that continued the intervention. Recognizing this variation, we conducted a number of analyses stratifying by site continuation vs. discontinuation (**Figures S2 and S3**; **Tables S3 and S4**) which show, conclusively, the differences in outcomes, retaining an ITT approach.

-       How many participants (and proportions) completed the intervention and how many lost to follow-up at 2.5 years? Among sites that continued the active intervention, how many participants (and proportions) completed the intervention and how many lost to follow-up at 6.5 years? Among sites that did not continue with active intervention due to administrative reasons, how many participants (and proportion) continued to provide annual data and how many were lost to follow-up at 6.5 years? I note Consort Diagram in Figure 1 but it did not seem to differentiate between sites that continued and sites that discontinued active intervention.

We appreciate the need for clarity and have modified the CONSORT diagram (**Figure 1**) with additional details. Among 1146 randomized at baseline, we had 2.5 year assessment data for 1027 participants (516 intervention and 511 usual care; and there were 26 deaths, 46 refused further participation, and 47 were unable to be contacted). At 6.5 years, assessment data were available for 854 participants (417 (including 51 deaths) intervention and 437 (including 45 deaths)) usual care; 61 refused further participation, and 112 were unable to be contacted). Of these, 288 intervention and 305 usual care participants were at sites that continued implementing the quality improvement approach and 129 intervention and 132 usual care participants were at sites that discontinued the quality improvement approach.

-       Results, first paragraph, please provide SD for means and IQR for median as well as p-values for all the comparisons

Aligned with this request, we have added the SD, IQR, and p-value estimates for comparisons in the results **(*Page 11, lines 380-390*)**.

-       Results, fifth paragraph, suggest to present data for microvascular and macrovascular complications separately as well as combined.

Noting this recommendation, we now present the data for microvascular and macrovascular endpoints **(*Page 12, lines 410-420*)** in addition to the combined estimates **(*Page 12, lines 422-428*)**.

-       Contact time with healthcare professionals is one of the determinants of goal attainment. Can the authors provide information on contact time or frequency of clinic attendance between the two arms

Thank you for the recommendation – unfortunately, we do not have data on contact time, but have reported the frequency of clinic visits between the arms. We removed the study assessment visits and focused on reporting only visits that were clinician- or patient-driven. We found that the intervention group had a median of 3 clinic visits per year (range: 0-27) and the usual care group had a median of 1 visit per year (range: 1-20). This has been added to the text **(*Page 11, lines 392-393*).**

-       Results, first paragraph, last sentence on medication use at 6.5 years, would be better shifted to the third paragraph on results at 6.5 years of follow-up.

Thank you for the recommendation – we have revised accordingly **(*Page 12, line 404-407*)**.

-       Results, fourth paragraph, for MACE, it appears that both absolute risk reduction and HR were not statistically significant and should be stated as such.

Aligned with this point, we have clarified this **(*Page 12, lines 411-413*)**.

-       Results, fourth paragraph, "…these results were compatible with as much as a 42% reduction or a 25% increased risk.", presumably, the authors were referring to the 95% CI but to me, this is irrelevant because the HR was not statistically significant. Suggest to delete.

To clarify, we had stated the results in this manner as we were trying to comply with the recent American Statistical Association guidance on reporting confidence intervals *(see Wasserstein, Schirm, and Lazar:* <https://www.tandfonline.com/doi/full/10.1080/00031305.2019.1583913>*).* On your recommendation, we have removed this language and simplified the statement **(*Page 12, lines 413-414, 425, and 428*)**.

-       Results, line 340 "there were no differences in multiple risk factor control by social or demographic factors". Better to specify the factors, "there were no differences in multiple risk factor control by age group, sex, BMI, education, income, diabetes duration, BMI, HbA1c and systolic BP at baseline".

In keeping with the Reviewer’s comment, we have edited this **(*Page 12, line 404*)**.

-       Table 1 -
o       Waist circumferences should be presented in men and women separately

Response: We have added the values for waist circumference for men and women separately **(*Table 1)***
o       Present eGFR in addition to creatinine level

Response: The values for eGFR calculated using the 2009 CKD-EPI equation are now included in ***Table 1***

Reference: Levey AS, Stevens LA, Schmid CH, et al. A new equation to estimate glomerular filtration rate. Annals of Internal Medicine. 2009;150(9):604–612. doi:10.7326/0003-4819-150-9-200905050-00006
o       Under comorbidities, proportion with diabetic kidney disease, retinopathy and neuropathy at baseline would be useful

Response: We have now included this information in ***Table 1***
o       What about proportion of patients using RAAS inhibitors?

Response: proportions of patients using RAAS inhibitors has been added to ***Table 1***.

-       Figure 2-4 - are these results from participants from sites that continued intervention, or participants from all the sites?

Response: In the primary analysis, we included data for all participants. Figures 2-4, therefore, represent data for all enrolled participants. Tables S3 and S4 show results obtained from sites that continued intervention (S3) and sites that discontinued (S4) **(*Supplement 2*)**.

-       Table S3 and S4 - the number of participants in the intervention group (n=575) and usual care group (n=571) is the same for both tables but S3 refers to results obtained from sites that continued intervention and S4 refers to results obtained from sites that discontinued

Response: Thank you for noting this error. We have corrected the tables with the appropriate n’s: Table S3 refers to sites that continued (Overall n=735; Intervention n=370; Usual care n=365) and Table S4 refers to sites that discontinued (Overall n=411; Intervention n=205; Usual care n=206) **(*Supplement 2*)**

-       The authors explained that the lack of between-group difference in incidence of MACE was possibly due to a younger age and better CV profile at baseline. Inadequate follow-up length is another potential explanation, given that MACE would take longer to develop.

As advised, we have adjusted the language regarding the incidence of MACE events and the possibility that we may have had inadequate follow-up **(*Page 12, line 468*)**.

-       Line 408, I would have reservation using the term "legacy effect", which is more reserved for when discussing effects on incident clinical events, when describing persistent changes in metabolic indices after intervention ended.
To clarify, we were using legacy effect to imply that changes during the 2.5 year period may have had impacts on longer-term event rates, but this is a fair point and we have changed the description and avoided “legacy effect” **(*Lines 359 and 484*)**.

**Reviewer #3**: In this clinical trial study, the authors aimed to assess the long-term efficacy of a multicomponent quality improvement (QI) strategy in managing cardiovascular risk factors in South Asian individuals with poorly controlled type 2 diabetes. Conducted at ten outpatient diabetes clinics in India and Pakistan from 2011 to 2019, 1146 participants were randomized to receive either the QI strategy, involving a non-physician care coordinator and a clinical decision support system, or usual care. After a median follow-up of 6.5 years, the study found that patients in the QI strategy group were significantly more likely to achieve and sustain control of multiple risk factors compared to the usual care group. This improvement was linked to a notable reduction in both microvascular and macrovascular complications. The benefits were specifically pronounced in clinics that continued the QI strategy throughout the study period. The findings underscore the importance of maintaining practice changes in clinical settings to improve health outcomes for people with diabetes. Overall, the study appears to be methodically robust and well-executed, with findings that could be valuable in shaping clinical practice and informing future policy making. Below are my specific comments on the manuscript:

1.      Title: I think you would like to say "…at 6.5 years follow-up"?
Thank you for this comment. We have updated the title accordingly.

2.      Introduction, Line 163-165: The introduction mentions the high prevalence of diabetes complications and mortality in South Asia, but it does not provide specific prevalence rates or sources for these statements. Including recent statistics and referencing authoritative sources would strengthen the claims.
We appreciate this point. However, there are no nationally representative data on diabetes complications nationally for India or other South Asian countries. As such, we have used the available data from the International Diabetes Federation Atlas.

3.      Introduction, Line 172-177: The introduction could benefit from specifying the nature of past research efforts in high-income countries and their outcomes, rather than just stating "a number of strategies" have been implemented.

In keeping with this recommendation, we have added some examples of quality improvement initiatives and strategies used in high-income countries **(*Page 6, lines 180-181*)**.

4.      Method, Line 240: What is DS-HER? Please provide the full name for the first time.

Thank you for this comment – clarifying here that DS-EHR is spelt out on ***line 210*** for the first time.

5.      Method, Line 250: suggested revision "patients were enrolled between March 10, 2011 and December 15, 2012."

We appreciate the suggestion and have revised accordingly **(*Page 8, line 265*)**.

6.      Method, Line 290-304: I would highly recommend the authors to include the mathematical formulas of the models employed for estimating treatment effects. A clear depiction of the models, with specific details on how covariates like age are incorporated (as continuous or categorical variables), as well as whether sites are treated as fixed or random effects, would enhance the reader's comprehension of the analytical approach.
We made a number of revisions in response **(*Pages 9-10, lines 310-364*).** We specify that we used the GEE equation approach to account for the correlation of observations within participants over time. For the multiple risk factor control, a log-binomial regression model was used. A Poisson regression model was used in case of convergence issues (Reference: Guangyong Zou, A Modified Poisson Regression Approach to Prospective Studies with Binary Data, American Journal of Epidemiology, Volume 159, Issue 7, 1 April 2004, Pages 702–706, <https://doi.org/10.1093/aje/kwh090>). The covariates included in the model were treatment group (Intervention/Usual care), time (categorical), site and baseline values. We checked for interactions between time and treatment group. As interaction was not significant, we did not include interaction terms in the model. The site was considered as a fixed effect in the model. In the adjusted model in addition to the above variables, we included age (continuous) and sex (male/ female) to report the adjusted risk difference and relative risk with 95% CI. The exchangeable correlation matrix was used.

$\mathrm{Log}\left( E\left[ \mathrm{MRF}_{ij} | X \right] \right)= \beta_{0}+ \beta_{1}I+ \beta_{2}T_{ij}+\beta_{3}S+\beta_{4}B$

Where

Subscript i indexes individuals

Subscript j indexes time point

MRF=achievement of multiple risk factor control (0, 1)

I=randomly assigned intervention group at baseline

T=time specified as a categorical variable

S=indicator for study site

B=Biomarkers at baseline for respective outcome (hba1c, SBP, DBP, LDL-c)

The equation for the adjusted model is as below:

$\mathrm{Log}\left( E\left[ \mathrm{MRF}_{ij} | X \right] \right)= \beta_{0}+ \beta_{1}I+ \beta_{2}T_{ij}+\beta_{3}S +\beta_{4}B +\beta_{5}D$

Where

Subscript i indexes individuals

Subscript j indexes time point

MRF=achievement of multiple risk factor control (0, 1)

I=randomly assigned intervention group at baseline

T=time specified as a categorical variable

S=indicator for study site

B=Biomarkers at baseline for respective outcome(hba1c, SBP, DBP, LDL-c)D= vector of baseline covariates (age [continuous], sex [male/female])

7.      Method, Line 290-304: I recommend that the authors provide a comprehensive description of the baseline characteristics included in the model. The Methods section should contain a subsection dedicated to the selection of covariates, outlining the rationale and criteria for their inclusion.
Thanks for the opportunity to clarify this. In the primary model, we included treatment group, time, site and respective baseline values of the outcome i.e. hab1c, SBP, DBP and LDLc. In adjusted models, in addition to variables listed above, we included age (continuous) and sex (male/female) as covariates **(*Page 9; lines 317-323*)**.

**Reviewer #4**: Thanks for the opportunity to read your manuscript. My role is statistical reviewer, so I have focused on the design, data, and analysis that are presented. I have put general comments first, followed by questions relevant to a specific section of the manuscript (with a page/line reference).

This manuscript presents a parallel RCT of multi-component intervention for patients with poorly controlled diabetes in India and Pakistan. The original trial followed participants for 2.5 years, this study was continued in 6/10 original sites for another 4 years. Participants were randomised individually to the intervention or usual care, without blinding of patients or clinical staff. Vascular events were coded by a blinded adjudication committee. The primary event of interest was achieved of risk factor control at the last follow-up, and several renal/CV events were considered post-hoc. Analysis was ITT, main analyses of the primary outcome were completed using log-binomial GEE to account for repeated measurements. For the CV/renal endpoints, these were analysed as time-to-event, including site ID and participant information. A secondary analysis examining if treatment was sustained was completed by comparing treatment effects in sites that did continue the intervention vs. those that did not. The effect of data missing-at-random (conditional on observed data) was assessed in analysis that used inverse probability weights. A range of sub-group analyses for the main and secondary outcomes were considered. In the IPW sensitivity analyses the treatment effect was shrunk back to the null - but still with evidence of a treatment effect overall.

This may have been addressed in the previous reviews, but I notice that in the attached protocol that CVD events are listed as a co-primary outcome. Was this just intended for the study using data up to 2.5 years?

Thank you for the opportunity to clarify this. As described above in response #2 to the Editor’s comments, the original trial that was powered to evaluate between-group achievement of multiple risk factor control and was designed to be 2.5 years in duration. We were able to secure funding to continue the intervention and monitoring of patients up until 6.5 years for vascular endpoints. As a result, the vascular endpoints were only considered outcomes at the 6.5 year follow-up timepoint.

Data that is missing-at-random is considered but given that that the treatment effect shrank back towards the null when using IPW, I think it is important to also undertake a sensitivity analysis of the potential impact of a missing-not-at-random mechanism (e.g. controlled imputations).
Response: We thank the reviewer for this very insightful comment. Based on the suggestion we conducted a reference-based sensitivity analysis to account for non-random attrition via multiple imputation **(*Page 11 lines 374-376, Table S9, and Page 13 lines 445-446*)**. Following *Cro et al 2016* and *Cro et al 2020*, we used the “Jump to Reference” (J2R) method for imputation, which assumes that patient behavior jumped to that of the usual care group after the intervention was discontinued. The joint distribution was treated as Multivariate Normal distribution (MVN) with mean vector from the patient's randomized arm up to their last observation time, post-deviation the mean vector follows that observed for the usual care group. The covariance matched the randomized arm for pre-deviation measurements and the usual care arm for the conditional components of post-given pre-deviation measurements*.* We generated 50 imputed datasets.

For the multiple risk factor control outcome at 6.5 years which was defined as HbA1c <7% & either BP <130/80 mmHg and/or LDLc <100 mg/dl [<70 mg/dl with a history of CVD], we imputed the dataset for each continuous variable i.e.(HbA1c, SBP, DBP and LDL-c) separately and then derived the multiple risk factor control variable from imputed continuous data. In each of the imputation models, we included all the variables which were included in the analysis model *(Austin,2021 et al)*. The covariates included in the imputation model were age, sex, baseline value, treatment group (intervention/usual care) and site. For the multiple and single risk factor control, a log-binomial regression model was used on the imputed dataset. The Poisson regression model was used in case of convergence issues.

The primary analysis suggested that the multiple risk factor control in the intervention group was superior (RR=1.77 [95%CI: 1.45, 2.17]) as compared to the usual care group. Under the jump to usual care sensitivity analysis, the intervention effect was greater (RR=1.83 [95%CI: 1.48, 2.26]). The results were similar for the other single risk factor control outcomes with smaller standard errors.

All the analysis was conducted using Stata version 16.0. The mimix command was used to run the imputation analysis *(ref: Cro et al. 2016)*

| Table: Sensitivity analysis results |  |  |  |
| --- | --- | --- | --- |
|  | **MRF** |  |  |
|  | **Treatment effect** | **Standard error** | **p value** |
| Primary analysis^1^ | 1.77 (1.45, 2.17) | 0.1824 | <0.001 |
| Primary analysis^2^ | 1.77 (1.45, 2.17) | 0.1824 | <0.00 |
|  |  |  |  |
| Sensitivity analysis |  |  |  |
| Jump to Usual care^1^ | 1.83 (1.48, 2.26) | 0.1067 | <0.001 |
| Jump to Usual care^2^ | 1.83 (1.48, 2.25) | 0.1064 | <0.001 |
| ^1^covariates included in the model treatment group (Intervention/Usual care), time, and site (fixed effect) | | | |
| ^2^covariates included in the model treatment group (Intervention/Usual care), time, age (continuous), sex (Male/Female) and site (fixed effect) | | | |

Reference:

Cro S, Morris TP, Kenward MG, Carpenter JR. Sensitivity analysis for clinical trials with missing continuous outcome data using controlled multiple imputation: A practical guide. Stat Med. 2020;39(21):2815-2842. doi:10.1002/sim.8569

Cro S, Morris TP, Kenward MG, Carpenter JR. Reference-based sensitivity analysis via multiple imputation for longitudinal trials with protocol deviation. Stata J. 2016 Apr;16(2):443-463. PMID: 29398978; PMCID: PMC5796638.

Austin PC, White IR, Lee DS, van Buuren S. Missing Data in Clinical Research: A Tutorial on Multiple Imputation. Can J Cardiol. 2021 Sep;37(9):1322-1331. doi: 10.1016/j.cjca.2020.11.010. Epub 2020 Dec 1. PMID: 33276049; PMCID: PMC8499698.

L292. Which correlation structure was used?
The Exchangeable correlation matrix was used in the model. Now we have added these details in the analysis section **(*Page 9, line 323*)**.

L301. What check was made for proportional hazards? There are some outcomes where this does not seem to hold, e.g. in Major clinical macro/microvascular events in Figure 3, there is no apparent difference between study arms for about 1 year. A time-dependent treatment effect might better describe this outcome than a single estimated hazard ratio (and is actually quite interesting).
We appreciate the opportunity to clarify: the proportional hazard assumption was checked based on Schoenfeld residuals after fitting a Cox proportional hazards model. The final Cox proportional hazards model included covariates age (continuous), sex (male/female) and site (fixed effect). The p-value was calculated for testing the null hypothesis that the PH assumption holds for all variables (global), and the test for each covariate. The p-value for global and for each covariate was not significant showing that the proportional hazard assumption was met.

L302. To adjust for site, was this included as covariate or as a random intercept?
Site identifiers were included as a covariate in the model assuming a fixed effect for sites **(*Page 9, line 321*)**.

L315. What variables were used to create the IP weights? Was the distribution of weights reasonable? i.e. any extreme values?
The inverse probability weights were generated for each timepoint and the variables included in the model were age, sex, education (categories: up to primary; secondary; college graduate and above), household income (Rs <10,000; Rs 10,001-20000; Rs >20,000), and treatment group (Intervention; Usual care) in the logistic regression model. There were some extreme weights, and we did the sensitivity analysis removing the extreme weights and the results were similar.

L306. This analysis is described as stratified but from the results it looks as though sub-group specific treatment effects were estimated with an interaction between subgroup and treatment allocation and subsequent marginal estimates of treatment effect. This is the best approach, so I would update this section to describe more accurately as it is not uncommon to see completely stratified analyses for sub-groups (which is not the ideal approach).
We thank the reviewer for pointing out this. The sub-group-specific treatment effects were estimated with an interaction between subgroup and treatment allocation and estimated the subsequent marginal estimates of treatment effect. We have now updated this section **(*Page 10, lines 363-365*).**

*“The sub-group specific treatment effects were estimated with an interaction between subgroup and treatment allocation and subsequent marginal estimates of treatment effect were estimated for multiple risk factor control and MACE outcomes by baseline age (20-44 vs. 45-65 vs. >65 years), sex, education (up to primary schooling, secondary schooling, or college graduate), monthly income (all US dollars: <$200, $200-400, vs. >$400), public vs. private healthcare setting, duration of diabetes (<7 vs. ≥ 7 years), BMI (<25 vs. 25-29.9 vs. ≥30 kg/m^2^), HbA1c (<9.0% vs. ≥9.0%), and systolic BP (<140 vs. ≥140 mmHg).”*

Figure 2 (and other figures). Is it possible to also change the colour of the line and dot so that each treatment group is a different colour?
Response: As suggested, we have now made changes to the line and dot colors in ***Figure 2***.

Figure 3. Can confidence intervals be added to these plots?
We thank the reviewer for the suggestion. We have now included confidence intervals in ***Figure 3***.

**Comments from the Academic Editor**

The study is conducted and reported according to the principle of individual participant level randomization. In practice however, the same care coordinator (CC) has a pivotal role in the delivery of the intervention in the intervention arm at each site. Because, the performance of CC will vary across site, this will translate into a clustering of the outcomes in the intervention arm at each site. Because similar clustering is less likely to occur in the control arm; the likely appropriate design for this study is a 'Randomized controlled trial with clustering in one arm (the intervention arm)'. This should have been accounted for at the design (sample size estimation) stage, but also at the data analysis status. Because the design of the data collection didn't account for this, the trial is likely underpowered. But with the data already collected, the minimum would be to account for the clustering at the data analysis stage (even just in secondary analysis). Adjusting for the site as done by the investigator would not take account of the clustering effects. [https://ind01.safelinks.protection.outlook.com/?url=https%3A%2F%2Fbmcmedresmethodol.biomedcentral.com%2Farticles%2F10.1186%2Fs12874-016-0249-5&data=05%7C02%7C%7C8c187c3fbd984dd8407f08dc2d7ae056%7C84df9e7fe9f640afb435aaaaaaaaaaaa%7C1%7C0%7C638435253007784115%7CUnknown%7CTWFpbGZsb3d8eyJWIjoiMC4wLjAwMDAiLCJQIjoiV2luMzIiLCJBTiI6Ik1haWwiLCJXVCI6Mn0%3D%7C0%7C%7C%7C&sdata=8xDna2jK850rI5VOaN7IssBgm7E8CXcFOTOIfMr58bU%3D&reserved=0](https://nam11.safelinks.protection.outlook.com/?url=https%3A%2F%2Fbmcmedresmethodol.biomedcentral.com%2Farticles%2F10.1186%2Fs12874-016-0249-5&data=05%7C02%7Cmkali%40emory.edu%7C6811b3fa236d41db537408dc2dc3dcd7%7Ce004fb9cb0a4424fbcd0322606d5df38%7C0%7C0%7C638435566472933703%7CUnknown%7CTWFpbGZsb3d8eyJWIjoiMC4wLjAwMDAiLCJQIjoiV2luMzIiLCJBTiI6Ik1haWwiLCJXVCI6Mn0%3D%7C0%7C%7C%7C&sdata=IBkqPg68qs83Mhv2XNwKGQokKODYOWMTxW5mSrmiRgA%3D&reserved=0)

We appreciate the insights of the Editor in this issue. By design, there was only one care coordinator per site, therefore taking into account the site effect is equivalent to accounting for the care coordinator. There were 3 sites where the care coordinator changed over the period. Based on this, we analyzed the sites where there was a change in the Care coordinator.

As suggested, we performed the analysis including patients clustered by site coordinator and site as a cluster variable in the model **(*Page 11 line 374, Table S10, and Page 13 line 446*)**. The results were almost similar with narrow CI when we included patients clustered by site coordinator and site as a cluster variable in the model.

|  | **GEE*** | **GEE^#^** | **Site co-ordinators as cluster^** |
| --- | --- | --- | --- |
| Multiple risk factor control | 1.76 (1.43, 2.16) | 1.79 (1.45, 2.20) | 1.78 (1.47, 2.15) |
| HbA1c <7% | 1.58 (1.32, 1.91) | 1.60 (1.32, 1.94) | 1.58 (1.34, 1.86) |
| BP <130/80 | 1.21 (1.11, 1.31) | 1.20 (1.11, 1.31) | 1.17 (1.09, 1.26) |
| LDLc <100 (<70 with a history of CVD) | 1.16 (1.08, 1.24) | 1.16 (1.08, 1.24) | 1.16 (1.09, 1.24) |

Multiple risk factor control defined as HbA1c <8% & either BP <130/80 mmHg and/or LDLc <100 mg/dl [<70 mg/dl with history of CVD]

* Models adjusted for site and respective baseline values

\# Models adjusted for age, sex, site and respective baseline values

^ with patients clustered by care coordinators and sites (Models adjusted for age, sex, site and respective baseline values)

Linked to the point above, change in CC within sites during the execution of the study will lead to further variation in the outcome within site; and at minimum, the investigators should comment on the stability of the employment of the CC during the study.

Thank you for this insightful point. At three sites, the Care Coordinators left the practice and were replaced by another individual who was trained to follow the protocol. We have added some discourse about this in the text **(*Page 14, lines 480-482*)**.

A minor point would relate to the forest plots: Figure 4 and Figure S4. The authors should specify log scale for the X-axis to achieve a balanced distribution of the confidence intervals about the estimates. They have currently specified the normal scale, which is not appropriate.
Thank you for the recommendation: we have now updated ***Figure 4*** and ***Figure S4*** specifying a log scale for the x-axis.
