# Supplementary material for: Effect of a multicomponent quality improvement strategy on sustained achievement of diabetes care goals and macrovascular and microvascular complications in South Asia at 6.5 years follow-up: Post hoc analyses of the CARRS randomized clinical trial
Source: PLoS Med. 2024 Jun 3;21(6):e1004335. doi: 10.1371/journal.pmed.1004335 (PMC11198027; doi:10.1371/journal.pmed.1004335)
Supplement: Attachment — Submitted filename: AuthorRESPONSES_2024-04-15.docx [file pmed.1004335.s006.docx]

*PLoS Medicine* Manuscript PMEDICINE-D-23-03727R2

“Effect of a Multicomponent Quality Improvement Strategy on Sustained Achievement of Diabetes Care Goals and Macrovascular and Microvascular Complications in South Asia at 6.5 years follow-up: post hoc analyses of the CARRS Randomized Clinical Trial"

**Comments and Author Responses**

**Editorial Comments**

GENERAL
Thank you for your very detailed and considered responses to previous editor and reviewer comments. Please see below for further comments which we require you address prior to publication.

Many of the editorial requests pertain to formatting and specific content requirements. Some may have already been incorporated into the manuscript and some may not apply but please review the complete list of items and ensure that all are included as relevant.

**FINANCIAL DISCLOSURE**
The funding statement should include: specific grant numbers, initials of authors who received each award, URLs to sponsors’ websites. Also, please state whether any sponsors or funders (other than the named authors) played any role in study design, data collection and analysis, the decision to publish, or preparation of the manuscript. If they had no role in the research, include this sentence: “The funders had no role in study design, data collection and analysis, decision to publish, or preparation of the manuscript.”
Thank you for the comments. We have added initials of authors who received each award, URLs to sponsors website and stated the role of funders in study design, data collection, analysis and the decision to publish or preparation of the manuscript.

**DATA AVAILABILITY STATEMENT**
PLOS Medicine requires that the de-identified data underlying the specific results in a published article be made available, without restrictions on access, in a public repository or as Supporting Information at the time of article publication, provided it is legal and ethical to do so. Please see the policy at
[https://ind01.safelinks.protection.outlook.com/?url=http%3A%2F%2Fjournals.plos.org%2Fplosmedicine%2Fs%2Fdata-availability&data=05%7C02%7C%7C850dd9a001a349d1100708dc5a4f7968%7C84df9e7fe9f640afb435aaaaaaaaaaaa%7C1%7C0%7C638484544595289896%7CUnknown%7CTWFpbGZsb3d8eyJWIjoiMC4wLjAwMDAiLCJQIjoiV2luMzIiLCJBTiI6Ik1haWwiLCJXVCI6Mn0%3D%7C0%7C%7C%7C&sdata=0cSkM9KnDHewGt8ZByYJwNbdchtpRtU36N1lBZYngjo%3D&reserved=0](https://apc01.safelinks.protection.outlook.com/?url=http%3A%2F%2Fjournals.plos.org%2Fplosmedicine%2Fs%2Fdata-availability&data=05%7C02%7Ckavita%40ccdcindia.org%7C7dbc0fbbcd6040fbe9ff08dc5a890bd6%7Cbc95d6f5d5b847a7a8f6886cd31a6f5b%7C0%7C0%7C638484791900461401%7CUnknown%7CTWFpbGZsb3d8eyJWIjoiMC4wLjAwMDAiLCJQIjoiV2luMzIiLCJBTiI6Ik1haWwiLCJXVCI6Mn0%3D%7C0%7C%7C%7C&sdata=7syJzFQv184S5WvKPG02PLj6xLso7e53ZhQTUySKArQ%3D&reserved=0)
and FAQs at 
[https://ind01.safelinks.protection.outlook.com/?url=http%3A%2F%2Fjournals.plos.org%2Fplosmedicine%2Fs%2Fdata-availability%23loc-faqs-for-data-policy&data=05%7C02%7C%7C850dd9a001a349d1100708dc5a4f7968%7C84df9e7fe9f640afb435aaaaaaaaaaaa%7C1%7C0%7C638484544595293197%7CUnknown%7CTWFpbGZsb3d8eyJWIjoiMC4wLjAwMDAiLCJQIjoiV2luMzIiLCJBTiI6Ik1haWwiLCJXVCI6Mn0%3D%7C0%7C%7C%7C&sdata=sdAzfxZ3i5rGvqfqx28hwUGjFdmN9BHJDDYZ33DUz9w%3D&reserved=0](https://apc01.safelinks.protection.outlook.com/?url=http%3A%2F%2Fjournals.plos.org%2Fplosmedicine%2Fs%2Fdata-availability%23loc-faqs-for-data-policy&data=05%7C02%7Ckavita%40ccdcindia.org%7C7dbc0fbbcd6040fbe9ff08dc5a890bd6%7Cbc95d6f5d5b847a7a8f6886cd31a6f5b%7C0%7C0%7C638484791900466955%7CUnknown%7CTWFpbGZsb3d8eyJWIjoiMC4wLjAwMDAiLCJQIjoiV2luMzIiLCJBTiI6Ik1haWwiLCJXVCI6Mn0%3D%7C0%7C%7C%7C&sdata=UdNAPR3I9hiF17YnKprGJlEBVote3GmTYBfZZuh2GgE%3D&reserved=0)  
PLOS defines the “minimal data set” to consist of the data set used to reach the conclusions drawn in the manuscript with related metadata and methods, and any additional data required to replicate the reported study findings in their entirety. Authors do not need to submit their entire data set, or the raw data collected during an investigation. Please submit the following data:
The values behind the means, standard deviations and other measures reported;
The values used to build graphs;
The points extracted from images for analysis.

Thank you for agreeing in principle to make all of your data available upon request. The Data Availability Statement (DAS) requires revision. For each data source used in your study: 
a) If the data are freely or publicly available, note this and state the location of the data: within the paper, in Supporting Information files, or in a public repository (include the DOI or accession number).
b) If the data are owned by a third party but freely available upon request, please note this and state the owner of the data set and contact information for data requests (web or email address). Note that a study author cannot be the contact person for the data.
c) If the data are not freely available, please describe briefly the ethical, legal, or contractual restriction that prevents you from sharing it. Please also include an appropriate contact (web or email address) for inquiries (again, this cannot be a study author).

Thank you for sharing the data availability statement guidelines.

Data for the NHLBI funded part of the trial is available through the NIH website, the rest of the trial data will be made available by reasonable request to the corresponding author or an independent person at CCDC central data repository, contact details provided in the online submission form.

**COMPETING INTERESTS**
All authors must declare their relevant competing interests per the PLOS policy, which can be seen here:
[https://ind01.safelinks.protection.outlook.com/?url=https%3A%2F%2Fjournals.plos.org%2Fplosmedicine%2Fs%2Fcompeting-interests&data=05%7C02%7C%7C850dd9a001a349d1100708dc5a4f7968%7C84df9e7fe9f640afb435aaaaaaaaaaaa%7C1%7C0%7C638484544595296512%7CUnknown%7CTWFpbGZsb3d8eyJWIjoiMC4wLjAwMDAiLCJQIjoiV2luMzIiLCJBTiI6Ik1haWwiLCJXVCI6Mn0%3D%7C0%7C%7C%7C&sdata=JV1%2BWxTEDkHjKBeQ4lUXttIPsq%2FE7v7JtQzNWRDM%2BFo%3D&reserved=0](https://apc01.safelinks.protection.outlook.com/?url=https%3A%2F%2Fjournals.plos.org%2Fplosmedicine%2Fs%2Fcompeting-interests&data=05%7C02%7Ckavita%40ccdcindia.org%7C7dbc0fbbcd6040fbe9ff08dc5a890bd6%7Cbc95d6f5d5b847a7a8f6886cd31a6f5b%7C0%7C0%7C638484791900472556%7CUnknown%7CTWFpbGZsb3d8eyJWIjoiMC4wLjAwMDAiLCJQIjoiV2luMzIiLCJBTiI6Ik1haWwiLCJXVCI6Mn0%3D%7C0%7C%7C%7C&sdata=t4go0%2B921FyxCZTulwUqxhe5i1j72C87Inuy7ad79U8%3D&reserved=0)
For authors with ties to industry, please indicate whether any of the interests has a financial stake in the results of the current study.

Thank you for your suggestions. We have added relevant competing interests as applicable.

LANGUAGE
We suggest avoiding use of the term, ‘Intervention patients’ (see abstract line 150) and suggest instead, ‘patients in the intervention arm’ or similar. Please check and amend throughout all sub-sections of the manuscript and supporting files as relevant.
Thank you for your suggestions. We have made necessary changes in the manuscript and revised the text throughout the manuscript and supporting files to say “patients in the intervention arm” instead of intervention patients.

STATISTICAL REPORTING
Please quantify the main results with 95% CIs and p values.
When reporting p values please report as <0.001 and where higher as p=0.002, for example. When reporting 95% CIs please separate upper and lower bounds with commas instead of hyphens as the latter can be confused with reporting of negative values.
Please include the actual amounts and/or absolute risk(s) of relevant outcomes (including NNT or NNH where appropriate), not just relative risks or correlation coefficients. (example for absolute risks: PMID: 28399126).
Thank you for the comments. We have checked all estimates and reported 95% CI, p-values, NNT as per your guidance.

ABSTRACT
Please structure your abstract using the PLOS Medicine headings (Background, Methods and Findings, Conclusions).
Please combine the Methods and Findings sections into one section, “Methods and findings”.
Abstract Background: Please provide context of why the study is important. The final sentence should clearly state the study question.
Abstract Methods and Findings:
Please ensure that all numbers presented in the abstract are present and identical to numbers presented in the main manuscript text.
Please include the study design, population and setting, number of participants, years during which the study took place, length of follow up, and main outcome measures.
Please quantify the main results with 95% CIs and p values. When reporting p values please report as <0.001 and where higher as p=0.002, for example. When reporting 95% CIs please separate upper and lower bounds with commas instead of hyphens as the latter can be confused with reporting of negative values.
Please include the important dependent variables that are adjusted for in the analyses.
Please include the actual amounts and/or absolute risk(s) of relevant outcomes (including NNT or NNH where appropriate), not just relative risks or correlation coefficients. (example for absolute risks: PMID: 28399126). 
Please include a summary of adverse events if these were assessed in the study.
In the last sentence of the Abstract Methods and Findings section, please describe the main limitation(s) of the study's methodology.
Abstract Conclusions:
Please address the study implications without overreaching what can be concluded from the data; the phrase "In this study, we observed ..." may be useful.
Please interpret the study based on the results presented in the abstract, emphasizing what is new without overstating your conclusions.
Please avoid vague statements such as "these results have major implications for policy/clinical care".  Mention only specific implications substantiated by the results.
Please avoid assertions of primacy ("We report for the first time....")
Thank you for the comments. We have now structured the abstract as per the journal guidelines and added both 95% CIs and p-values.

**AUTHOR SUMMARY**
At this stage, we ask that you include a short, non-technical Author Summary of your research to make findings accessible to a wide audience that includes both scientists and non-scientists. The authors summary should consist of 2-3 succinct bullet points under each of the following headings:
• Why Was This Study Done? Authors should reflect on what was known about the topic before the research was published and why the research was needed.
• What Did the Researchers Do and Find? Authors should briefly describe the study design that was used and the study’s major findings. Do include the headline numbers from the study, such as the sample size and key findings.    
• What Do These Findings Mean? Authors should reflect on the new knowledge generated by the research and the implications for practice, research, policy, or public health. Authors should also consider how the interpretation of the study’s findings may be affected by the study limitations. In the final bullet point of ‘What Do These Findings Mean?’, please describe the main limitations of the study in non-technical language.
The Author Summary should immediately follow the Abstract in your revised manuscript. This text is subject to editorial change and should be distinct from the scientific abstract. Please see our author guidelines for more information: [https://ind01.safelinks.protection.outlook.com/?url=https%3A%2F%2Fjournals.plos.org%2Fplosmedicine%2Fs%2Frevising-your-manuscript%23loc-author-summary&data=05%7C02%7C%7C850dd9a001a349d1100708dc5a4f7968%7C84df9e7fe9f640afb435aaaaaaaaaaaa%7C1%7C0%7C638484544595299741%7CUnknown%7CTWFpbGZsb3d8eyJWIjoiMC4wLjAwMDAiLCJQIjoiV2luMzIiLCJBTiI6Ik1haWwiLCJXVCI6Mn0%3D%7C0%7C%7C%7C&sdata=hwNxyEgyqYbZ2b2Kh2aFiOamMDR55x8MLpfM59qy6Jo%3D&reserved=0](https://apc01.safelinks.protection.outlook.com/?url=https%3A%2F%2Fjournals.plos.org%2Fplosmedicine%2Fs%2Frevising-your-manuscript%23loc-author-summary&data=05%7C02%7Ckavita%40ccdcindia.org%7C7dbc0fbbcd6040fbe9ff08dc5a890bd6%7Cbc95d6f5d5b847a7a8f6886cd31a6f5b%7C0%7C0%7C638484791900478845%7CUnknown%7CTWFpbGZsb3d8eyJWIjoiMC4wLjAwMDAiLCJQIjoiV2luMzIiLCJBTiI6Ik1haWwiLCJXVCI6Mn0%3D%7C0%7C%7C%7C&sdata=qNhsXMyqKUKB5nCjwHIHDxIsJ9EZBsHyCJhKwLhZWws%3D&reserved=0)

Thank you for sharing the guidelines on Authors Summary. We have now added a plain language non-technical Authors Summary at the end of the Abstract. (lines 185-215).

INTRODUCTION
Please ensure that you address past research and explain the need for and potential importance of your study. Indicate whether your study is novel and how you determined that. If there has been a systematic review of the evidence related to your study (or you have conducted one), please refer to and reference that review and indicate whether it supports the need for your study.
Thank you for the comments. We have reviewed the introduction and it’s aligned as per your guidelines.

METHODS and RESULTS
Line 237 – please specify whether informed consent was written or oral.
Line 242 – was the usual care also in line with local/regional guidance? We presume it would be which might be helpful to clarify for the reader to appreciate the potential variability (or not) in the usual care delivered.
Line 269 – not sure that ‘imminently’ is the best word to use here. Are you referring to the frequency of adverse event reporting (i.e., monthly, annually) or the immediacy (i.e., adverse events are reported immediately that they are documented)? Please revise for clarity.
Line 276 – suggest, ‘…defined through laboratory tests of blood and urine…’
Line 365 onwards – when reporting the p value you could probably just detail p (i.e., removing the word ‘value’ to make things a little more concise and accessible but we leave it to your discretion.
Thank you for the comments. We have made the following changes in the manuscript

- Participants informed consent was written
- Yes, usual care practices were in line with local hospital norms
- We changed the word “imminently” to the reporting timelines adhered during the trial for SAE notification within 24 hours.
- Accepted the suggestion “laboratory tests of blood urine….”
- P-values are reported along with 95% CIs.

TABLES
Throughout (including the supporting files) please ensure that each table is affiliated to a title and caption that clearly describes the table content without the need to refer to the text. Please ensure that all abbreviations, including those used in statistical reporting, are defined in the caption or a footnote.
Please indicate whether analyses are adjusted or unadjusted. Where adjusted analyses are presented, please detail in the caption/footnote the factors which are adjusted for. To help facilitate transparent data reporting, please present the unadjusted analyses for comparison. 
Please ensure that all abbreviations, including those used in statistical reporting, are clearly defined in the caption or a footnote.
Thank you for the comments. We have verified the formatting of Tables as per your guidelines.

FIGURES
Please see here for guidelines on submitting and citing figures [https://ind01.safelinks.protection.outlook.com/?url=https%3A%2F%2Fjournals.plos.org%2Fplosmedicine%2Fs%2Ffigures%23loc-how-to-submit-figures-and-captions&data=05%7C02%7C%7C850dd9a001a349d1100708dc5a4f7968%7C84df9e7fe9f640afb435aaaaaaaaaaaa%7C1%7C0%7C638484544595303723%7CUnknown%7CTWFpbGZsb3d8eyJWIjoiMC4wLjAwMDAiLCJQIjoiV2luMzIiLCJBTiI6Ik1haWwiLCJXVCI6Mn0%3D%7C0%7C%7C%7C&sdata=y%2BoX%2Bs20zxkNUxOXoD60unbH3gDCAQfveeTs53Jvs%2BY%3D&reserved=0](https://apc01.safelinks.protection.outlook.com/?url=https%3A%2F%2Fjournals.plos.org%2Fplosmedicine%2Fs%2Ffigures%23loc-how-to-submit-figures-and-captions&data=05%7C02%7Ckavita%40ccdcindia.org%7C7dbc0fbbcd6040fbe9ff08dc5a890bd6%7Cbc95d6f5d5b847a7a8f6886cd31a6f5b%7C0%7C0%7C638484791900486282%7CUnknown%7CTWFpbGZsb3d8eyJWIjoiMC4wLjAwMDAiLCJQIjoiV2luMzIiLCJBTiI6Ik1haWwiLCJXVCI6Mn0%3D%7C0%7C%7C%7C&sdata=%2BcaeMfA%2F8J8OBOsx6EKkVzD0oNO2i9MbRMbWrNISwLs%3D&reserved=0)
Throughout (including the supporting files) please ensure that each figure is affiliated to a title and caption that clearly describes the figure content without the need to refer to the text. 
Throughout (including the supporting files) please consider avoiding the use of red and green in order to make your figures more accessible to those with colour blindness.
Please ensure that all abbreviations, including those used in statistical reporting, are defined in the caption or a footnote.
Please indicate whether analyses are adjusted or unadjusted. Where adjusted analyses are presented, please detail in the caption/footnote the factors which are adjusted for. To help facilitate transparent data reporting, please present the unadjusted analyses for comparison. 
Where 95% CIs are presented, please also present p values and report as detailed above under ‘statistical reporting’. 
Please ensure that you define the meaning of all dots and lines for the reader in the caption/footnote.
Figure 2 – as you report survival as percentage of the population, it would be helpful to the reader to detail the actual numbers for a reference point. As above, all figures should be interpretable without the need to refer to the text.
Figure 3 – please define the meaning of the blue and red shaded areas in the caption/footnote.
Thank you for the comments. All Figures are updated/formatted as per your instructions. We have added actual numbers for a reference point in all figures.

DISCUSSION
Line 429 – please replace ‘Conclusions’ with ‘Discussion’. Done
Please ensure that you present and organize the Discussion as follows: a short, clear summary of the article's findings; what the study adds to existing research and where and why the results may differ from previous research; strengths and limitations of the study; implications and next steps for research, clinical practice, and/or public policy; one-paragraph conclusion. Please avoid the use of sub-headings such that the discussion reads as continuous prose.
Line 433 – suggest, ‘…receiving usual care for diabetes’ as the word diabetes currently isn’t mentioned in the opening paragraph and probably should be. Done
Line 436 – when reporting risk reduction, as detailed above under ‘statistical reporting’ please report the actual and not just the relative risk as the latter can overstate the true risk. Done
Line 457 – sentence ending, ‘…: for example, .’ seems incomplete please revise/amend. In the discussion section, inclusion of “for example” was a typo, we have removed it.
Line 475 – as above regarding language, suggest, ‘…in patients assigned to receive the QI intervention compared to usual care.’ Or similar. Done.
Line 494 – suggest, ‘… could vary.’ Done.
Line 518 – please move the ethics statement to an appropriate part of the methods section. Please remove the funding statement and include these details only in the manuscript submission form when you re-submit your manuscript. Done
Line 591 onwards – please remove this statement and include in the manuscript submission form when your resubmit the manuscript.
Line 614 – please remove this statement and revise in line with guidance above under ‘data availability’. Please include the revised statement only in the manuscript form when you resubmit the manuscript.
Thank you for the comments. Data availability statement is added to the manuscript form.

REFERENCES
For in-text reference callouts please place citations in square parentheses separate by commas without spaces and preceding punctuation. For example, [1,3,6] or [1-3]. Please check and amend throughout all sub-sections of the manuscript and supporting files.
In the bibliography please ensure that you list up to but no more than 6 author names followed by et al.
For all web references please ensure you include an, ‘Accessed [date].’ Please replace ‘Cited’ with ‘Accessed’.
Journal name abbreviations should be those listed in the National Center for Biotechnology Information (NCBI) databases.
Thank you for the suggestions. References are aligned as per your instructions.

SUPPORTING INFORMATION
Please cite your Supporting Information as outlined here: [https://ind01.safelinks.protection.outlook.com/?url=https%3A%2F%2Fjournals.plos.org%2Fplosmedicine%2Fs%2Fsupporting-information&data=05%7C02%7C%7C850dd9a001a349d1100708dc5a4f7968%7C84df9e7fe9f640afb435aaaaaaaaaaaa%7C1%7C0%7C638484544595307303%7CUnknown%7CTWFpbGZsb3d8eyJWIjoiMC4wLjAwMDAiLCJQIjoiV2luMzIiLCJBTiI6Ik1haWwiLCJXVCI6Mn0%3D%7C0%7C%7C%7C&sdata=5HlGi3i5cV%2FUeGe4%2FVxmvKUT%2BA6gJu7o4uCSrDszc1I%3D&reserved=0](https://apc01.safelinks.protection.outlook.com/?url=https%3A%2F%2Fjournals.plos.org%2Fplosmedicine%2Fs%2Fsupporting-information&data=05%7C02%7Ckavita%40ccdcindia.org%7C7dbc0fbbcd6040fbe9ff08dc5a890bd6%7Cbc95d6f5d5b847a7a8f6886cd31a6f5b%7C0%7C0%7C638484791900492234%7CUnknown%7CTWFpbGZsb3d8eyJWIjoiMC4wLjAwMDAiLCJQIjoiV2luMzIiLCJBTiI6Ik1haWwiLCJXVCI6Mn0%3D%7C0%7C%7C%7C&sdata=r68c23FyvYGtNvKFDG4bWTE%2FZgoBoZhFBA6c1zBGZzg%3D&reserved=0)
Please apply all guidance detailed above to all supporting files.
In the published article, supporting information files are accessed only through a hyperlink attached to the captions. For this reason, you must list captions at the end of your manuscript file. You may include a caption within the supporting information file itself, as long as that caption is also provided in the manuscript file. Do not submit a separate caption file.
Trial protocol (s)  – you may wish to revise the contact details of the investigators prior to publication as individual email addresses are detailed here.
CONSORT Checklist – thank you for including this. Please amend to refer to section and paragraph numbers as opposed to page (or line) numbers as the latter often change at the time of publication.
Thank you for the comments. We have revised the CONSORT checklist as per your recommendations.

SOCIAL MEDIA
To help us extend the reach of your research, please detail any X (formerly Twitter) handles you wish to be included when we tweet this paper (including your own, your coauthors’, your institution, funder, or lab) in the manuscript submission form when you re-submit the manuscript.

Thank you for these suggestions. We have added social media handles of co-authors and our institutions.

**Comments from Reviewers**

**Reviewer #1**: In their revised manuscript, the authors have adequately addressed all the queries and suggestions made by the reviewers

Thank you for your feedback.

**Reviewer #2**: I thank the authors for addressing my comments.

The revised manuscript is significantly improved in its clarity.

My two minor comments:
Third paragraph (starting at Line 269) under "Assessments" explains how outcomes were defined and is better placed in the section "Outcomes". Done.

In Discussion section, the paragraph starting at Line 457 seems incomplete.

Apologies for the oversight. Inclusion of “for example” was a typo and we have now corrected that.

Otherwise, I am happy with the revision.!
We appreciate the positive sentiments, attentive review, and recommendations to improve the manuscript.

**Reviewer #3**:

**Reviewer #4**: Thanks for the revised manuscript and responses to my original review. The additions to the methods make the manuscript much clearer, thank you. I agree that the sensitivity analyses (assuming shift to usual care) show results consistent with the main analysis. Just a few small points remaining below.

L149. A minor point, but 'groups were similar' is more accurate. Done.

L153. Should be 'hazard ratio' Done

L155. I would probably use 'no evidence of between group first macrovascular events' instead of 'statistical differences' here.. Done

Thank you for your additional comments/feedback. We have incorporated these changes in the revised manuscript.
